# *N*-Oxide S–O chalcogen bonding in conjugated materials†

**DOI:** 10.1039/d0sc06583h

**Published:** 2021-01-07

**Authors:** Geoffrey S. Sinclair, Robert C. M. Claridge, Andrew J. Kukor, W. Scott Hopkins, Derek J. Schipper

**Affiliations:** Department of Chemistry, University of Waterloo Waterloo Canada derek.schipper@uwaterloo.ca; Waterloo Institute for Nanotechnology Waterloo Canada; Institute for Polymer Research Waterloo Canada

## Abstract

Non-covalent bonding interactions, such as chalcogen bonding, can have a substantial effect on the electronic and physical properties of conjugated polymers and is largely dependent on the strength of interaction. Functional groups that are traditionally used to instill chalcogen bonding such as alkoxy or fluorine substituents can demand challenging synthetic effort, as well as have drastic effects on the electronics of a π-system. The incorporation of a *N*-oxide functionality into bithiazole-containing materials, a synthetically simple transformation, has been entirely overlooked until now. A systematic analysis of the effects of *N*-oxidation on the electronic and physical properties of bithiazole-containing materials has been undertaken. *N*-Oxidation has been found to affect the electronic band gap through increase of the HOMO and lowering of the LUMO. Furthermore, exceptionally strong intramolecular S–O chalcogen bonding interactions in the bithiazole core contribute to rigidification of the conjugated system. Computational analysis of this system has shown this *N*-oxide chalcogen bonding interaction to be significantly stronger than other chalcogen bonding interactions commonly exploited in conjugated materials.

## Abstract 2

*N*-Oxide incorporation into thiazole-containing conjugated materials can have substantial effects on both the electronic and physical properties. A notably strong non-covalent S–O interaction is observed between the *N*-oxide and neighbouring sulfur.

## Graphical abstract

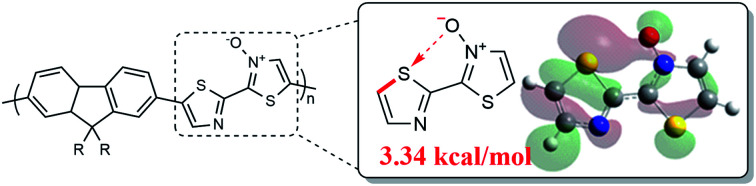

## Introduction

Non-covalent interactions have been explored in π-conjugated polymers and small molecules as a unique method for influencing the electronic and physical properties of materials.^1^ Most notably, non-covalent interactions have been used as conformational locks through the induction of planarity between adjacent arene or alkene units. This induced planarity increases the p-orbital overlap, therefore extending the effective conjugation of a π-system and reducing a material's electronic band gap.^2^ Historically, this conformational locking of conjugated systems has been achieved through the tethering of adjacent aromatic units with covalent alkyl bridges; however, it is often synthetically less complex to exploit non-covalent interactions for this purpose.^3–5^ The introduction of planarity between conjugated units is one of a handful of common strategies employed as a method of band gap tuning, alongside the incorporation of alternating donor–acceptor motifs, stabilizing the quinoidal state, and promoting interchain interactions.^6–8^ The deployment of these design strategies in conjugated small molecules and polymers has been imperative for the fine-tuning of optoelectronic properties as the demand for high-performance materials has continued to progress. This has enabled the development of next-generation organic electronic applications such as photovoltaics (OPVs),^9–11^ light-emitting diodes (OLEDs),^12–14^ and field-effect transistors (OFETs),^15,16^ which provide numerous advantages over state-of-the-art silicon-based electronics, such as easy processability, flexibility, and tunability.^17–19^

Among the non-covalent interactions commonly exploited in conjugated organic materials, the most heavily studied ones are those of S–O chalcogen bonding.^20,21^ These interactions are often observed between thiophene sulfur and adjacent alkoxy groups on neighbouring aromatic units. Chalcogen bonding increases planarization of a conjugated system by counteracting the associated negative steric interactions.^22–26^ While these alkoxy substituents can positively affect the p-orbital overlap, their highly electron-donating nature can have a destabilizing electronic effect on a conjugated molecule by significantly increasing the HOMO energy level.^27^ Similar planarization effects are observed in conjugated materials through S–N chalcogen bonding interactions. These interactions can be highly beneficial as heterocycles containing these atoms are prevalent in conjugated small molecules and polymers. Further rigidification effects have also been observed by introducing S–F bonding between thiophene sulfur and installed fluorine groups.^28–32^ Fluorine can be an ideal substituent for inducing chalcogen bonding interactions as it does not have a major electronic or steric effect on the conjugated system. However, incorporation of these atoms at specific locations to allow for S–F chalcogen bonding interactions can add significantly to the synthetic complexity of the conjugated material. Finally, in some cases, non-traditional hydrogen bonding interactions (O–HC_sp^2^_ and N–HC_sp^2^_) have also been observed as conformational locks in organic semiconductors. They are most notably studied in materials based on the diketopyrrolopyrrole (DPP) functionality.^33^

Though thiophene-based polymers, such as poly(3-hexylthiophene), dominate the list of suitable conjugated polymers for organic electronics, our research group has been interested in investigating thiazole-based conjugated small molecules and polymers. Thiazole has been highlighted in a number of reports as a unique alternative heterocycle to thiophene in conjugated materials.^34–38^ These heteroarenes can instill a higher level of planarity in a conjugated system due to the lack of steric hindrance at the nitrogen 3-position, which allows for both increased p-orbital overlap and strong interchain stacking. Thiazoles are also more ideal than thiophenes for use in N-type semiconductors due to their relative electron deficiency and low-lying HOMO, which provides a higher degree of air stability.^39^ As a result of these factors, thiazoles have become increasingly studied as prominent motifs for incorporation in conjugated organic materials.

**Fig. 1:**
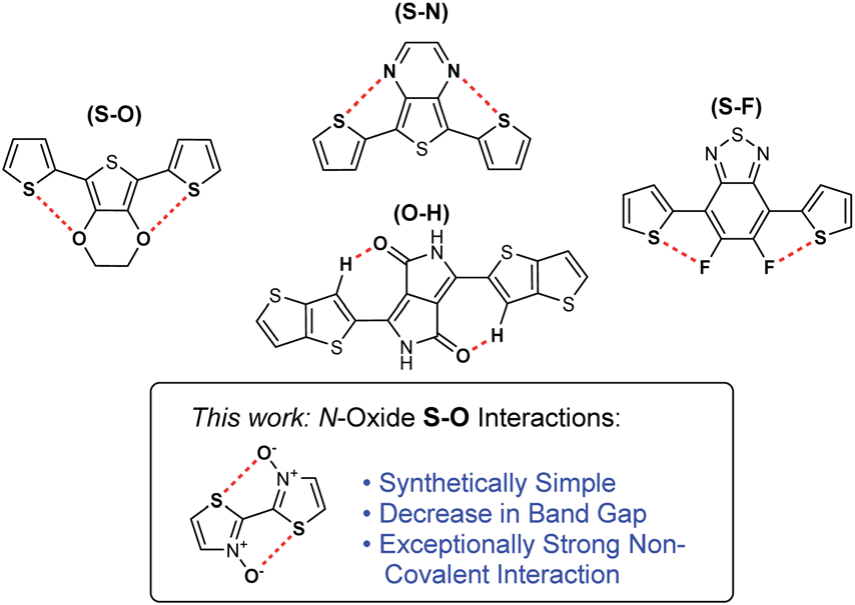
Non-covalent interactions employed in conjugated materials.

Recently, we reported novel approaches towards the synthesis of bithiazole-containing conjugated polymers through both transition-metal-free dehydrative polymerization, and *ipso*-arylative polymerization, of thiazole *N*-oxides.^40,41^ While these types of transition-metal-free approaches to the synthesis of polyheterocyclic conjugated polymers are rare, bithiazole *N*-oxide-containing polymers produced in this manner represent a new class of unexplored polymers with the potential for unique physical and electronic properties. Herein, we describe the investigation of conjugated small molecules and polymers possessing bithiazole *N*-oxide and *N*,*N*′-dioxide moieties, and the effect that the *N*-oxide functionality has on both the electronic and physical properties of the materials (Fig. 1). A systematic study, both experimental and computational, is employed to reveal a chalcogen S–O interaction with a far greater strength than other chalcogen bonding interactions traditionally employed in the design of conjugated materials.

## Results and discussion

### Optical and electrochemical properties

**Fig. 2:**
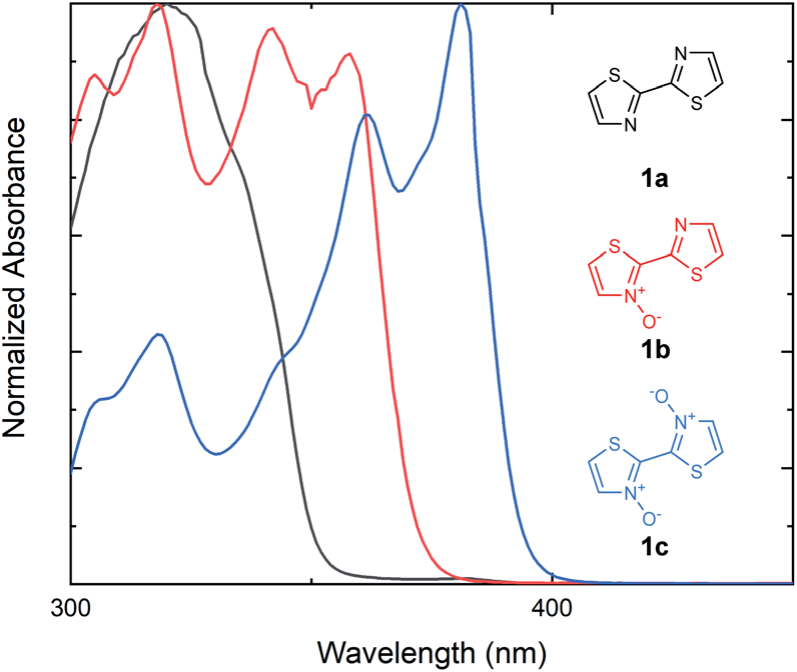
UV-vis absorption spectra for the series of bithiazoles (**1a**, **1b** and **1c**) with increasing oxidation. Absorption spectra measured in CHCl_3_.

Our investigations into the effects of *N*-oxide incorporation on bithiazole-containing conjugated systems began with the analysis of bithiazole optical properties through UV-vis absorption and fluorescence. In general, non-covalent interactions used to impart planarity manifest as a bathochromic shift and a decrease in the optical band gap of the material. We began by studying bithiazole series **1**: including 2,2′-bithiazole (**1a**), as well as its singly (**1b**) and doubly (**1c**) oxidized forms. 2,2′-Bithiazole **1a** was synthesized through the oxidative homocoupling of thiazole using copper acetate.^42^ Bithiazole *N*-oxide **1b** was achieved through our previously reported *ipso*-arylative condensation, while further oxidation of this product with *m*-CPBA yielded **1c**.^41^ The UV-vis absorption spectra of bithiazoles **1a**, **1b** and **1c** were recorded in CHCl_3_ to determine the optical band gaps of the small molecules (Fig. 2). Upon increasing the level of *N*-oxidation, a bathochromic (red) shift is observed in the onset of absorption from 352 nm (**1a**), to 374 nm (**1b**), and to 398 nm (**1c**) corresponding to a decrease in the optical band gap of approximately 0.2 eV per each successive *N*-oxidation. We hypothesized that this bathochromic shift could possibly be due to increased planarity of the conjugated system; however, it could also be simply due to electronic effects of oxygen incorporation. Since bithiazole **1a** is known to be a relatively planar substrate, increased orbital overlap could potentially be caused by rigidification of the molecular structure from chalcogen bonding between the *N*-oxide and adjacent thiazole sulfur. Alternatively, the decrease in the optical band gap could be due to the *N*-oxide incorporation resulting in a push–pull electron effect, increasing and lowering the HOMO and LUMO respectively. Notably, upon introduction of the *N*-oxide functionalities, there is an appearance of vibrational fine structures in the absorption band of bithiazoles **1b** and **1c**. In conjugated systems, the appearance of these fine structures in absorption spectra is frequently attributed to molecular rigidity.^43–45^

**Fig. 3:**
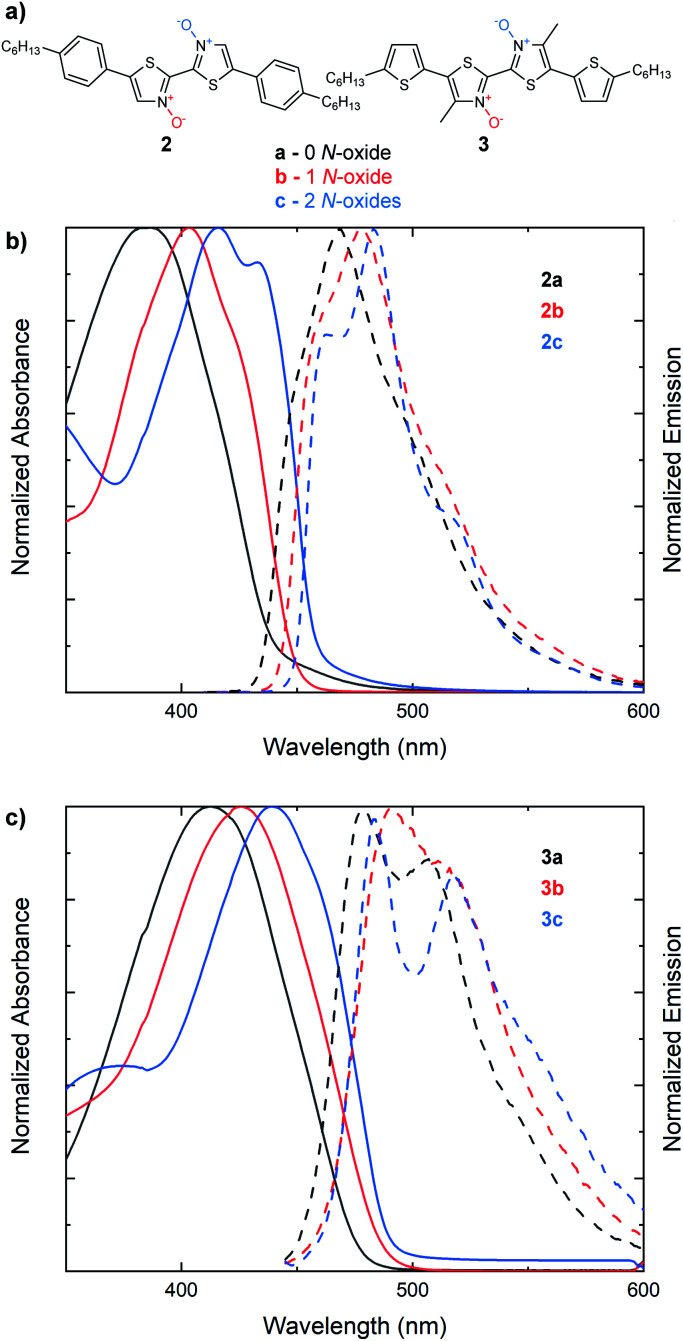
(a) Series of extended π-conjugated bithiazoles possessing 0 (black, a), 1 (red, b) or 2 (blue, c) *N*-oxides. (b) UV-vis absorption spectra (solid) and steady-state fluorescence spectra (dashed) of **2a**, **2b** and **2c**. (c) UV-vis absorption spectra (solid) and steady-state fluorescence spectra (dashed) of **3a**, **3b** and **3c**. Absorption spectra measured in CHCl_3_. Emission spectra measured in CH_2_Cl_2_.

We next sought to understand if the same trend in the optical band gap found with increasing *N*-oxidation in 2,2′-bithiazoles **1a–1c** would be observed in larger bithiazole-containing small molecules (Fig. 3a). With extended π-conjugated systems, the frontier molecular orbitals would be less dependent on the bithiazole itself, and therefore, the effect may not be visible in the absorption spectra. The bis(4-hexylphenyl) derivative (**2a**) and its *N*-oxide (**2b**) and *N*,*N*′-dioxide (**2c**) variants were synthesized and characterized by UV-vis absorption spectroscopy. Like what has been previously observed for series **1**, a bathochromic shift was detected in the absorption spectra upon increasing the number of *N*-oxides present (Fig. 3b). This red shift corresponded to a minimal decrease in the optical band gap of 0.05 eV from each successive *N*-oxidation. Likewise, bis(5-hexylthiophenyl) small molecule **3a** and its *N*-oxide (**3b**) and *N*,*N*′-dioxide (**3c**) variants were synthesized. Accordingly, a total decrease of 0.08 eV was observed in the optical band gap of this series upon oxidation to the *N*,*N*′-dioxide (Fig. 3c). Characterizing the emission spectra of series **2** and **3** and increasing the level of *N*-oxidation also resulted in a decreased Stokes shift, another notable indicator for increased molecular rigidification.^46^

**Fig. 4:**
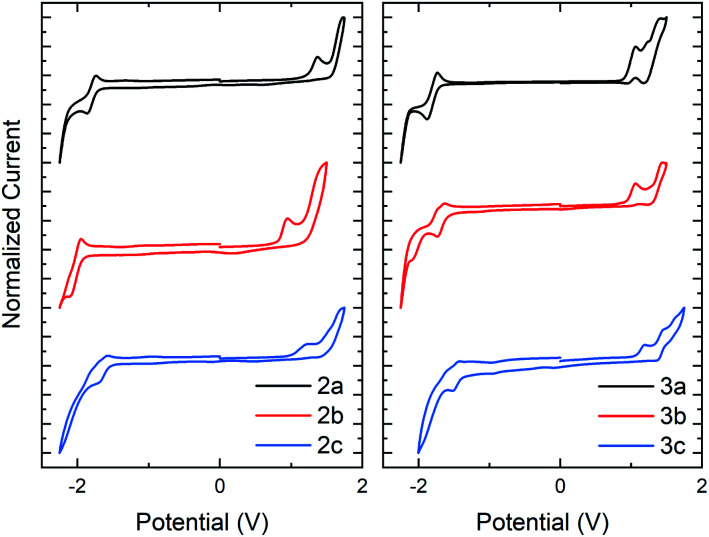
Cyclic voltammetry traces in CH_2_Cl_2_ with a Bu_4_NPF_6_ electrolyte for series **2** and series **3**. HOMO and LUMO calculated with respect to the ferrocene oxidation peak.

The electrochemical (EC) band gap of series **2** and **3** was next determined by cyclic voltammetry (CV). Voltammetry measurements of series **2** showed a decrease in the band gap of approximately 0.16 eV per *N*-oxide functionality (Fig. 4a). This band gap decrease was due to an average increase of the HOMO by 0.07 eV and decrease of the LUMO by 0.09 eV upon *N*-oxide installation. While the lowering of the LUMO energy level upon increasing *N*-oxidation of the system was expected, it was interesting to discover than conversion of thiazole to thiazole-*N*-oxide also resulted in an increase in the HOMO, indicating that the molecules become easier to oxidize. This is supportive of the hypothesis that the decrease in the HOMO–LUMO gap is due to an electronic push–pull effect of the bithiazole *N*-oxide functionality, which would increase and lower the HOMO and LUMO as observed. Electrochemical reversibility of the reduction peak visible for **2a**, remained observable in the CV trace of **2b**, and to a smaller extent for **2c** (though solubility may be a factor in peak visibility). Voltammetry measurements of series **3** displayed a similar trend of a decrease of approximately 0.1 eV in the band gap upon *N*-oxidation (Fig. 4b). For these thiophene small molecules, however, the change in the HOMO energy level was minimal compared to that for series **2**. This is likely due to the thiophenes having a larger contribution towards the HOMO than the phenyl substituents. The optical and EC measurements for series **2** and **3** are summarized in Table 1.

**Table 1:** Optical and electrochemical properties of extended π-conjugated bithiazole series **2** and **3**

Entry	*E*_g[EC]_ (eV)	HOMO (eV)	LUMO (eV)	*E*_g[opt]_ (eV)	Abs_Max_ (nm)	Em_Max_ (nm)	Stokes (eV)
**2a**	2.95	−5.82	−2.87	2.80	387	468	0.55
**2b**	2.78	−5.74	−2.96	2.75	403	479	0.49
**2c**	2.62	−5.68	−3.06	2.70	416	483	0.41
**3a**	2.66	−5.49	−2.82	2.60	413	480	0.42
**3b**	2.56	−5.48	−2.91	2.54	426	491	0.39
**3c**	2.48	−5.50	−3.02	2.52	439	483	0.26

**Fig. 5:**
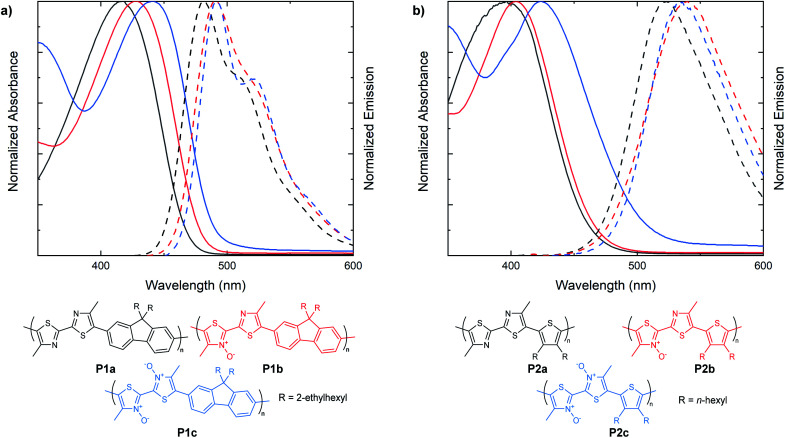
UV-vis absorption/steady-state fluorescence spectra of **P1a**, **P1b** and **P1c**. (c) UV-vis absorption/steady state fluorescence spectra of **P2a**, **P2b** and **P2c**. Absorption spectra measured in CHCl_3_. Emission spectra measured in CH_2_Cl_2_.

Following the examination of *N*-oxide effects on the extended π-conjugated small molecules, we next endeavored to synthesize bithiazole-containing conjugated polymers with various levels of thiazole *N*-oxidation. Bithiazole polymers incorporating a fluorene spacer with solubilizing chains (**P1**), were prepared *via* direct arylation polymerization (**P1a**, **P1c**)^47–49^ as well as dehydration polymerization (**P1b**) (Fig. 5a).^40^ A second series of polymers, possessing 3,4-dihexylthiophene spacers (**P2**), was prepared with varying *N*-oxidation levels in the same manner (Fig. 5b). Each bithiazole polymer was characterized by gel permeation chromatography (GPC) against a polystyrene standard in THF in order to obtain molecular weight (*M*_N_, *M*_W_) and poly-dispersity index (PDI) parameters. Optical properties were determined through UV-vis absorption and emission spectroscopy in CH_2_Cl_2_, and are compiled in Table 2. From the absorbance and emission data of the polymers we discovered that the same trends previously observed in the bithiazole small molecules (**1–3**) continue to hold true for series **P1** and **P2**. For fluorene-polymer series **P1**, a bathochromic shift was observed upon increased *N*-oxidation, with onsets of absorbance shifting from 475 nm, to 495 nm and 520 nm for **P1a**, **P1b** and **P1c**, respectively. This corresponds to an average decrease of 0.1 eV in the optical band gap upon each successive *N*-oxidation. This bathochromic trend is also observed in the absorption spectra of thiophene-polymer series **P2**. Interestingly, the initial *N*-oxidation from **P2a** to **P2b** resulted in a decrease of only 0.05 eV, while incorporation of a second *N*-oxide in polymer **P2c** resulted in a larger decrease of 0.15 eV. Overall, the apparent effect of *N*-oxide incorporation on the optical band gap of bithiazole-containing conjugated small molecules and polymers remains small but notable. Both polymer series **P1** and **P2** also display an overall decrease in the Stokes shift from the unoxidized polymers to the *N*,*N*′-dioxides.

**Table 2:** Optical, electrochemical and physical properties of polymer series **P1** and **P2**

Entry	Mn (kDa)	PDI	HOMO (eV)	LUMO (eV)	*E*_g[EC]_ (eV)	*E*_g[opt]_ (eV)	Abs_Max_ (nm)	Em_Max_ (nm)	Stokes shift (eV)
**P1a**	18.2	2.3	−5.75	−2.80	2.95	2.61	417	482	0.41
**P1b**	20.4	2.2	−5.69	−2.86	2.83	2.51	427	491	0.38
**P1c**	14.0	2.3	−5.65	−2.95	2.70	2.41	441	491	0.29
**P2a**	20.6	2.2	−5.67	−2.71	2.96	2.61	396	523	0.76
**P2b**	17.0	2.7	−5.57	−2.96	2.61	2.56	404	540	0.77
**P2c**	6.0	2.0	−5.53	−3.02	2.51	2.41	423	533	0.61

The electrochemical band gaps for polymer series **P1** and **P2** were determined by linear sweep voltammetry (LSV). Voltammetry measurements of the bithiazole-containing conjugated polymers were performed in the solid state after drop casting a solution of the polymer onto a platinum working electrode. Correlating with what had been previously observed for the small molecules, LSV measurements displayed a reduction in the band gap of the polymers upon increasing the number of thiazole *N*-oxides. Again, this decrease in the electrochemical band gap upon *N*-oxidation occurs through both the lowering of the LUMO and increase of the HOMO. In the polymer series **P1**, the voltammetry determined HOMO was increased roughly by 0.05 eV with each successive oxidation, and was accompanied by a lowering of the LUMO by 0.06 and 0.09 eV with each respective oxidation.

The effect of *N*-oxidation on the HOMO and LUMO energy levels is relatively small compared to the addition of a single alkoxy (–OR) substituent to polythiophene, which shows a reduction in the band gap, solely through increase of the HOMO, by around 0.3 eV.^27^ The same joint lowering of the LUMO and increase of the HOMO were observed for series **P2**, although the effect of *N*-oxidation on the LUMO between the **P2a** and **P2b** was much more drastic at 0.25 eV.

### Solubility and thermal properties

Throughout our analysis of the materials' optoelectronic properties, a substantial decrease in solubility was noted for both the small molecules (**1–3**) and polymers (**P1**, **P2**) upon incorporation of each *N*-oxide functionality. The poor solubility of *N*,*N*′-dioxide polymers (**P1c** and **P2c**) is speculated to have been the cause for the lower extent of polymerization obtained compared to that of the bithiazole polymers also synthesized *via* direct arylation polymerization (**P1a** and **P2a**). This decrease in solubility is also a notable indicator of conjugated backbone rigidification and increased π-stacking that had previously been hypothesized from the Stokes shift and vibrational fine structures observed in the absorption spectra.

**Fig. 6:**
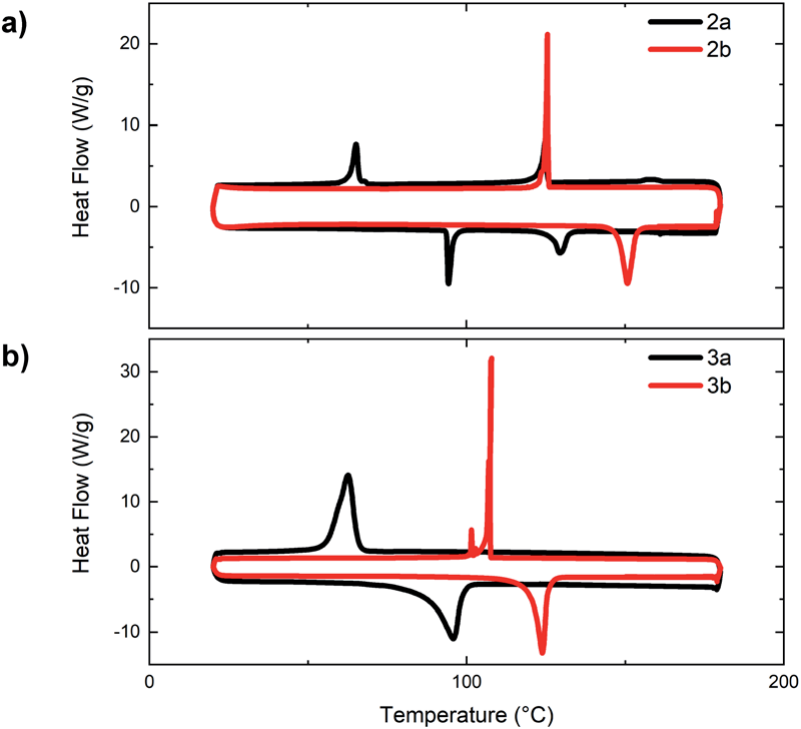
Differential scanning calorimetry traces of bithiazoles: (a) bis(hexylphenyl) **2a** and **2b** and (b) bis(hexylthiophenyl) **3a** and **3b**.

To further investigate the effects of *N*-oxidation on the physical properties of the bithiazole small molecules **2** and **3**, we sought to evaluate the thermal transitions of the materials by differential scanning calorimetry (DSC) (Fig. 6). Notably, upon increasing the level of bithiazole *N*-oxidation from **2a** to **2b** in the 4-hexylphenyl series, an increase is observed in both the melting temperature and crystallization temperature of 57 °C and 61 °C, respectively. Likewise, in the DSC traces of series **3**, possessing 5-hexylthiophenes, an increase in both thermal transitions of 28 °C (melting) and 45 °C (crystallization) is observed for **3a** and **3b**. No thermal transitions were observed for *N*,*N*′-dioxide molecules **2c** and **3c** prior to decomposition of the material occurring at approximately 180 °C (determined by thermogravimetric analysis in ESI, Fig. S2†). This observed increase in thermal stability can be indicative of the *N*-oxide-containing molecules (**2b** and **3b**) having more/stronger intermolecular interactions between the conjugated π-systems.^50^ Interestingly, it was observed in the DSC of 4-hexylphenyl small molecule **2a** (Fig. 6a, black) that the material proceeds through two exothermic and endothermic transitions, likely representing the formation of a liquid crystalline phase. This was not completely unexpected due to the structural similarity of **2a** with known liquid crystals.^51^

### Single-crystal XRD

**Fig. 7:**
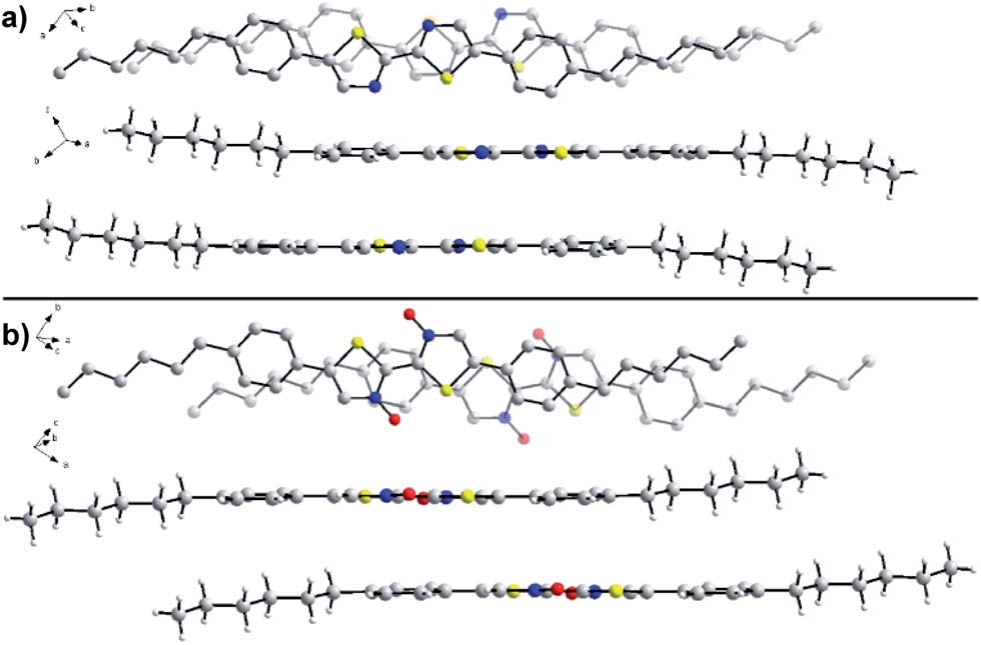
Single-crystal X-ray diffraction structures of (a) **2a** and (b) **2b** from a top-down and side-on view. Hydrogen atoms have been omitted from the top-down view for clarity. Atoms: carbon, gray; nitrogen, blue; oxygen, red; sulfur, yellow; hydrogen, white. Oxygen atoms in the structure of **2b** possess 50% occupancy.

To determine the effects of *N*-oxidation on molecular organization, we attempted to determine the solid-state packing of series **2** through analysis by single crystal X-ray diffraction (Fig. 7). Crystals of unoxidized **2a** and single *N*-oxide **2b** were obtained through slow evaporation of chloroform at room temperature. X-ray quality crystals were unable to be obtained for *N*,*N*′-dioxide **2c** due to its poor solubility and aggregation in solution that has been previously noted. While both molecules adopt the expected planar geometry, there are some noticeable differences in organization that may explain the observed changes to the material properties. In the crystal structure of **2a**, the N–C–C–N dihedral is slightly off-planar at 178.5° while the same dihedral is completely planar at 180.0° in the structure of **2b**. The intermolecular π–π stacking distance between bithiazole cores was found to be similar in the two molecules; 3.63 Å in the crystal structure of **2a** and 3.59 Å in the structure of **2b**. However, differences in the packing make a direct comparison difficult. *N*-Oxide **2b** packs with a greater degree of slip-stacking (5.7 Å from co-facial arrangement) compared to the slip-stacking of **2a** (3.3 Å). This is suspected to be due to the increased presence of short contact interactions between *N*-oxide oxygen and neighbouring molecules in **2b**. It is important to note that the *N*-oxide oxygens shown in the crystal structure of **2b** have an occupancy of 50% (only one oxygen per molecule). A notably short contact is present between the *N*-oxide oxygen and thiazole sulfur (2.58 Å) in the crystal of **2b**, indicative of the suspected intramolecular S–O chalcogen bonding interaction.

### Computational study

**Fig. 8:**
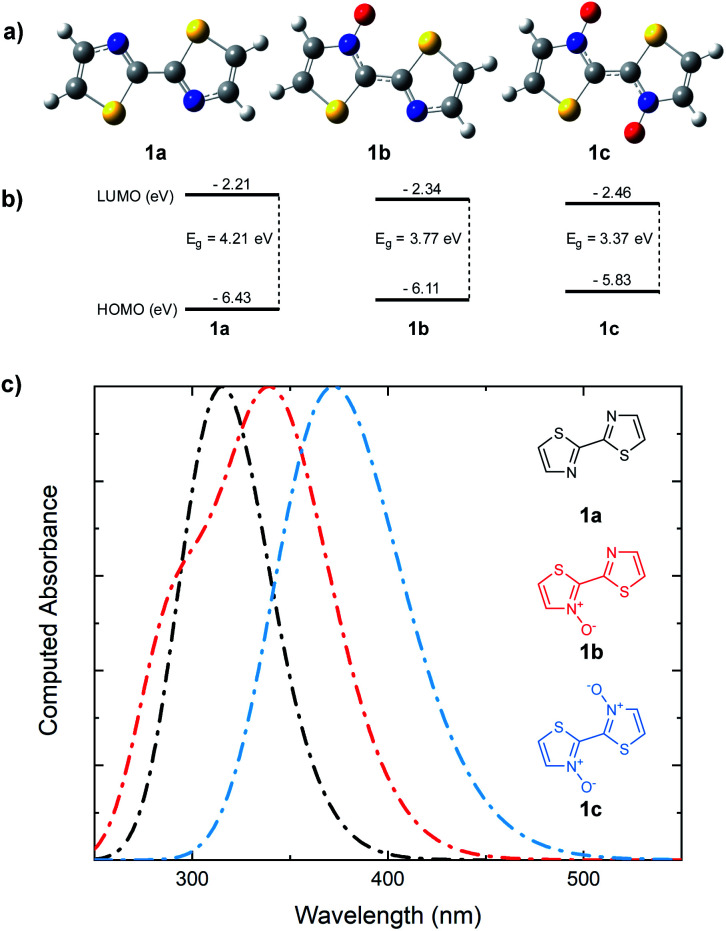
(a) DFT optimized structures of bithiazoles **1a**, **1b**, and **1c**. (b) DFT (B3LYP/6-311++g(d,p)) computed HOMO and LUMO energies. (c) TD-DFT computed UV-vis absorbance spectra.

Whether the cause for decrease in the band gap of the bithiazole-*N*-oxide small molecules and polymers was due to the electron donating/withdrawing effects of *N*-oxide incorporation, or through rigidification of the π-system's planarity, remained unclear. A computational study was thus performed in order to obtain a thorough understanding of what had been observed experimentally. The optimized structures of bithiazoles **1a–1c** were obtained using density functional theory (DFT) (B3LYP:6-311++g(d,p)). As expected, the optimal geometry of each bithiazole **1a–1c** possessed a fully planar molecular structure and N–C–C–N dihedral angle of 180°, even in **1a** in which no *N*-oxide is present (Fig. 8a). Examining the frontier energy levels of these optimized structures revealed that reduction in the HOMO–LUMO gap with *N*-oxidation is due to both a successive lowering of the LUMO and increase of the HOMO, corroborating what was observed in the polymer voltammetry measurements (Fig. 8b).

Time-dependent (TD)-DFT excited state calculations performed on the optimal structures were used to generate theoretical absorption spectra and optical band gaps (Fig. 8c). The simulated absorption spectra showcased a trend similar to that observed experimentally in Fig. 2, wherein a decrease in the optical band gap of 0.3 eV was observed upon each successive *N*-oxidation. As the optimal geometries for bithiazole **1a–1c** are all planar along the N–C–C–N dihedral, this was further evidence that the decrease in the band gap did not appear to be due to increased p-orbital overlap from planarization. For comparison, TD-DFT calculations were also used to compute the optical band gap of a model 2,2′-bioxazole system and its *N*-oxide and *N*,*N*′-dioxide equivalents (see ESI, Fig. S5†). Bioxazole was chosen to represent a similar electronic system to bithiazole, while removing the potential for S–O chalcogen bonding. Despite this inability, an average reduction of 0.53 eV per *N*-oxidation is observed in the calculated optical gaps for these model 2,2′-bioxazole compounds. This appears to affirm that the electronic effect of the *N*-oxide incorporation results in the bathochromic shift, and not increased planarity due to chalcogen bonding. Increased rigidity of the π-conjugated system from S–O interactions could, however, still be responsible for the observed properties related to molecular rigidification, including: the decrease in the Stokes shift, appearance of vibrational fine structures in the absorption spectra, the poor solubility and change in thermal transitions observed for the *N*-oxide-containing materials.

**Fig. 9:**
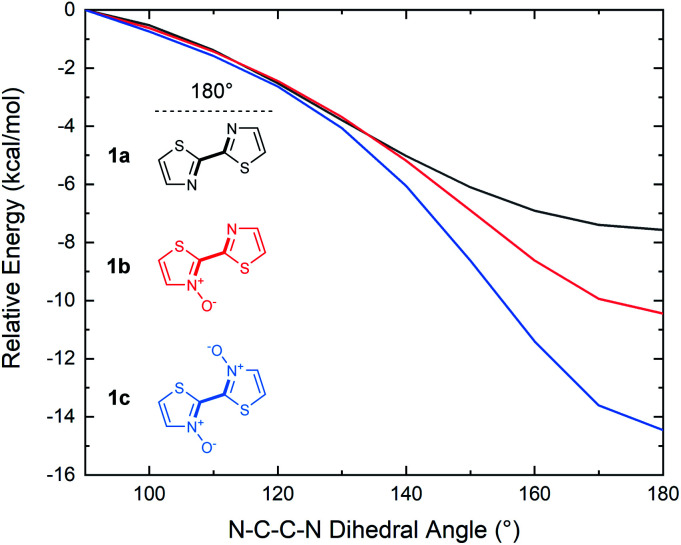
Relative energies of **1a**, **1b** and **1c** for conformations from 90–180° (N–C–C–N bond) in 10° intervals (dihedral angle denoted in bold). DFT optimization performed with B3LYP/6-311++g(d,p). CCSD(T) energy calculations performed with 6-311++g(d,p).

Although 2,2′-bithiazoles adopt a planar geometry, a higher barrier of rotation could assist in locking the molecular configuration, leading to strong intermolecular interactions such as π-stacking. The torsional barrier for several conjugated materials has been previously reported in an extensive computational study by Ratner and co-workers to be on the order of several kcal mol^−1^.^52^ These barriers were determined for model conjugated molecules by calculating single point energies at 10° intervals along the rotation of the dihedral angle from 0°–180°. The same analysis was, therefore, performed to determine the energy of rotation about the N–C–C–N dihedral angle of our bithiazoles (**1a–1c**). Coupled cluster theory (CCSD(T)) single point energy calculations were executed on the DFT optimized structures of compounds **1a–1c**, with the dihedral angle in question scanned from 90° < *θ* < 180° in 10° intervals, with 180° being the low energy planar conformation (Fig. 9). From this torsional barrier analysis we were able to determine that the energy barrier to break planarity (that is to rotate from a N–C–C–N dihedral angle of 180° to 90°) increased with successive *N*-oxidation from 7.6 to 10.4 to 14.5 kcal mol^−1^ for **1a**, **1b** and **1c**, respectively. The dihedral angles from 0° to 90° were omitted due to the high steric barrier caused by the oxygen atoms eclipsing in *N*,*N*′-dioxide **1c** (see ESI, Fig. S7†). The computed rotational barrier of compound **1c** from 180° to 90° is notably high when compared to the chalcogen (S–O) bonding energies reported by Ratner and coworkers, in spite of the limited steric bulk contributing to planarization in bithiazole series **1**.^52^ A high torsional barrier indicates that *N*-oxidation indeed has a rigidifying effect on the bithiazole bond rotation, likely leading to the observed changes in physical properties. For comparison, the same torsional study was performed on 2,2′-bioxazole, its *N*-oxide, and *N*,*N*′-dioxide, wherein a decrease in the torsional barrier from the planar to 90° conformation was observed upon successive oxidation. This is likely due to *N*-oxidation only contributing to the steric bulk while lacking any stabilizing interactions (see ESI, Fig. S6†).

**Fig. 10:**
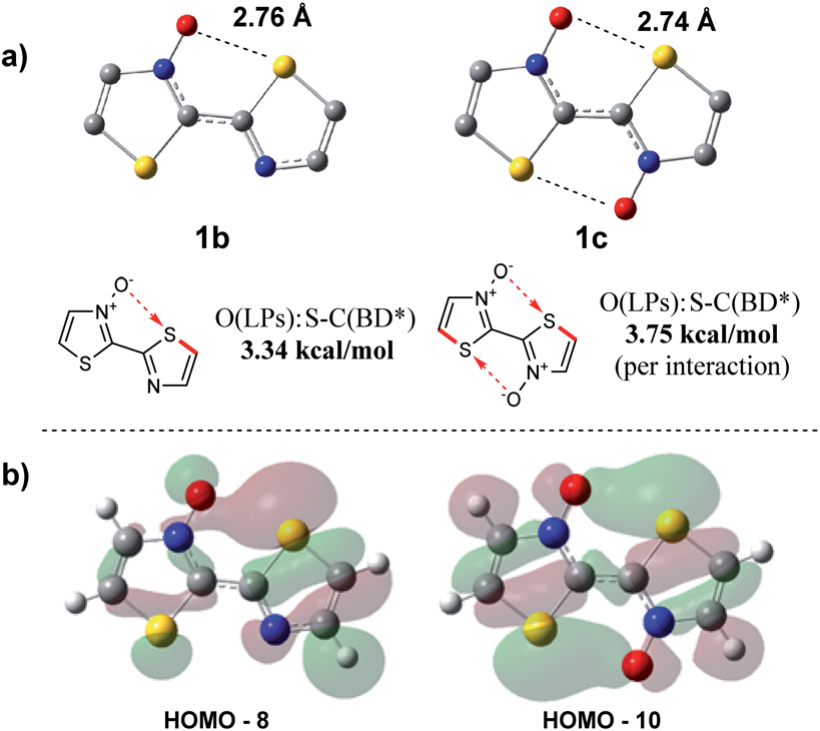
(a) S–O stabilization interactions determined through natural bonding orbital (NBO) on DFT optimized structures, and (b) molecular orbitals showing donation from the oxygen lone pair to the S–C antibonding orbital.

As the presence of the *N*-oxide functionality was undoubtedly influencing the bithiazole rigidity, we sought to computationally evaluate the strength of any chalcogen bonding interaction occurring. A computational tool used to determine the interactions that contribute to geometric preferences is natural bonding orbital (NBO) analysis.^53^ NBO calculations were performed on the DFT optimized structures of bithiazoles **1b** and **1c** in order to examine the contribution of electron density donation from the *N*-oxide oxygen to the adjacent thiazole sulfur. When analysing the second order perturbation energies between the oxygen lone pairs (donor) and sulfur–carbon antibonding orbital (acceptor) in **1b** it was calculated that there is a 3.34 kcal mol^−1^ stabilization energy provided by this interaction. Furthermore, the NBO calculations of **1c** showed that there was a total 7.49 kcal mol^−1^ increase in stabilization energy from the two S–O interactions (Fig. 10a). These values correspond well to the stabilization energy predicted from comparing the relative energies of **1a**, **1b** and **1c** at an N–C–C–N dihedral of 180°. The computed bond distance between the thiazole sulfur and *N*-oxide oxygen in the optimized structures of **1b** and **1c** is ∼2.75 Å, well less than the combined van der Waals radii of the two atoms (3.25 Å) and consistent with previously calculated S–O chalcogen bond distances in conjugated molecules.^26^ These bond distances are also consistent with the S–O bond distance of our previously reported crystal structure for 4,4′,5,5′-tetramethyl-[2,2′-bithiazole] 3-oxide (2.74 Å).^40^

Further support for the strength of *N*-oxide chalcogen bonding was provided by quantum theory of atoms in molecules (AIM). These AIM analyses revealed bond critical points (BCPs) between the *N*-oxide oxygen and thiazole sulfur in both **1b** and **1c** (AIMALL generated figures available in ESI, Table S8†).^54,55^ The charge densities (*ρ*) of 0.0199 and 0.0210 a.u., for **1b** and **1c** respectively, indicate incipient chemical bonding in the range of that expected between hydrogen-bonding (*ρ* ≈ 10^−3^ to 10^−2^ a.u.) and covalent bonding (*ρ* > 10^−1^ a.u.). The positive Laplacians (*∇*^2^*ρ*) of +0.0622 (**1b**) and +0.0642 a.u. (**1c**) are also indicative of electron density donation, similar to that found in hydrogen-bonding, and consistent with what is known about S–O interactions.^56^ This chalcogen bonding interaction contributes to some of the populated, molecular orbitals observable in Fig. 10b.

**Fig. 11:**
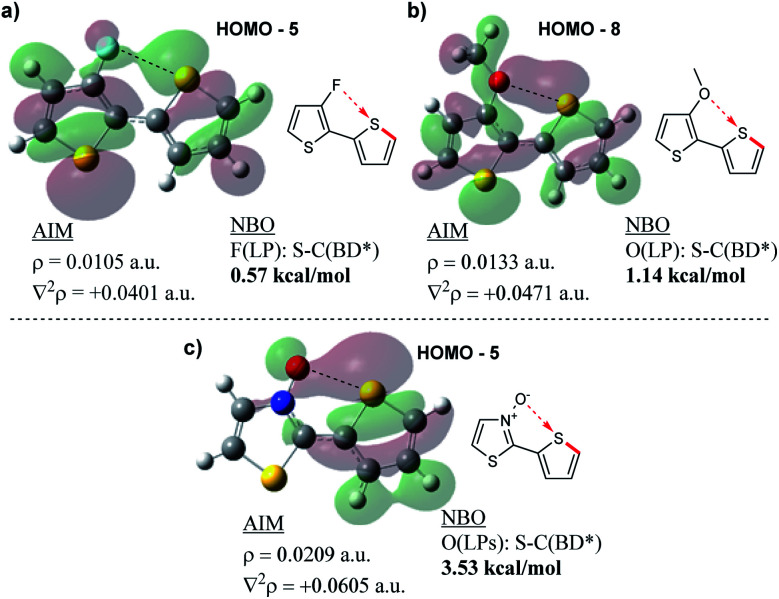
Results of AIM and NBO analyses showing (a) F–S, (b) O–S and (c) *N*-oxide O–S non-covalent interactions.

Following the results of this study on the bithiazole *N*-oxide S–O interaction in **1a–1c**, we were interested in quantifying the obtained values with other chalcogen bonding interactions generally exploited in conjugated systems. We therefore performed NBO and AIM analyses on a model bithiophene compound possessing a 3-fluorine substituent (Fig. 11a) and a bithiophene possessing a 3-methoxy substituent (Fig. 11b) in order to investigate the chalcogen bond strength of the corresponding S–F and S–O interactions. For consistency, analysis was also performed on 2-(thiophen-2-yl)thiazole *N*-oxide (Fig. 11c). The AIM analyses for all model compounds showed BCPs between the F/O donor and S acceptor, and as expected, possessed positive Laplacian values. The charge densities of the F- (*ρ* = 0.0105 a.u.) and MeO-substituted (*ρ* = 0.0133 a.u.) compounds were significantly lower than that of the thiazole *N*-oxide-containing compound (*ρ* = 0.0209 a.u.) indicating a weaker interaction between the electron density donor and sulfur, more along the lines of a hydrogen bond. Additionally, while the NBO analyses in each case showed some stabilization in the second order perturbation energies from donation into the S–C antibonding orbital, the stabilization energy provided by this interaction in the F- and MeO-substituted thiophenes equated to only 0.57 kcal mol^−1^ and 1.14 kcal mol^−1^, respectively, while the *N*-oxide contributed 3.53 kcal mol^−1^ (in line with what was observed for **1b** and **1c**). Thus, computationally it appears (by NBO and AIM) that the chalcogen bonding found in the bithiazole *N*-oxides is a far stronger non-covalent interaction than other examples of chalcogen bonding typically employed to induce conformational locking in conjugated materials.

## Conclusions

Developing new strategies to modify the electronic and physical properties of conjugated polymers remains an imperative task for their widescale deployment in next-gen electronics. Exploiting phenomena such as chalcogen bonding and other non-covalent interactions to induce planarity or affect the intramolecular interactions of conjugated small molecules and polymers has become commonplace. We have identified a new type of chalcogen bonding in conjugated small molecules and polymers possessing bithiazole-*N*-oxide and *N*,*N*′-dioxide motifs. While *N*-oxidation does instill what we believe is an electronic effect on the conjugated system, electrochemical analysis of the band gaps of model compounds has shown this effect to be due to increase of the HOMO and lowering of the LUMO. The presence of vibronic fine structures in the absorption spectra, decrease in the Stokes shift upon bithiazole *N*-oxidation, a decrease in solubility and increase in thermal stability are indicative of an increase in the molecular rigidity of the system. This rigidity has been attributed to strong chalcogen bonding S–O interactions occurring between the *N*-oxide and adjacent thiazole sulfur. An extensive computational investigation carried out on these systems has revealed an increase in the torsional barrier about the bithiazole bond by an average of 3–4 kcal mol^−1^ per oxygen installed, resulting in a more rigid conjugated system. Furthermore, NBO and AIM analysis was used to evaluate the strength of the *N*-oxide–sulfur interaction, which was found to be far greater in bond strength than that of other commonly employed S–O or S–F chalcogen bonding interactions. The ease of oxygen transfer for the formation of thiazole *N*-oxides compared to the synthetic difficulty of installing alkoxy or fluorine substituents makes this an attractive method to induce rigidity in conjugated materials for organic electronic applications.

## Conflicts of interest

There are no conflicts to declare.

## Supplementary Material

SC-012-D0SC06583H-s001

SC-012-D0SC06583H-s002
