# Supplementary material for: N-Oxide S–O chalcogen bonding in conjugated materials
Source: Chem Sci. 2021 Jan 7;12(6):2304–12. doi: 10.1039/d0sc06583h (PMC8179281; doi:10.1039/d0sc06583h)
Supplement: SC-012-D0SC06583H-s001 [file SC-012-D0SC06583H-s001.pdf]

Electronic Supplementary Material (ESI) for Chemical Science.
This journal is © The Royal Society of Chemistry 2020

N-Oxide S-O Chalcogen Bonding in Conjugated Materials

Geoffrey S. Sinclair,[a,] Robert C. M. Claridge,[a] Andrew J. Kukor,[a] W. Scott Hopkins,[a,b] Derek J. Schipper [a,b*]

[a,*] Department of Chemistry, University of Waterloo, Waterloo, Ontario, Canada N2L 3G1

[b] Waterloo Institute for Nanotechnology, University of Waterloo, Waterloo, Ontario, Canada, N2L 3G1

Table of Contents

1. General Experimental Procedures
2. Synthetic Procedures
3. Absorption Spectra
4. Thermal Data
5. Voltammetry
6. Gel Permeation Chromatography
7. X-Ray Crystal Data
8. References
9. Computational Data
10. Spectral Data

1. General Experimental Procedures

Reactions were performed under an air atmosphere unless otherwise specified in the procedure. Reaction solvents used were reagent grade or HPLC grade. Anhydrous tetrahydrofuran was dried and purified through a JC Meyer solvent-purification system (SPS). Chemical reagents were purchased from Millipore-Sigma or Oakwood Chemical. Reactions were monitored using aluminum-backed silica thin-layer chromatography (TLC) plates (Kieselgel 60 F_{254}, Merck). Developed TLC plates were examined under a UV lamp (254 nm/ 365 nm). Flash chromatography was performed using 230–400 mesh silica gel (SiliCycle).

^{1}H-NMR spectra were recorded on a Brüker AVANCE300 (300 MHz) δ or Brüker AC300 (300 MHz) δ NMR spectrometer. ^{13}C-NMR spectra were broad band decoupled and recorded on a Brüker AVANCE300 (75.5 MHz) δ or Brüker AC300 (75.5 MHz) δ NMR spectrometer. Chemical shifts are reported in parts per million (ppm) relative to chloroform at (δ 7.28) for ^{1}H-NMR and (δ 77.0) for ^{13}C-NMR. The following abbreviations are used for NMR peak multiplicities: s, singlet; d, doublet; t, triplet; q, quartet; p, pentet (quintet); dd, doublet of doublets; dt, doublet of triplets; m, multiplet; br, broad. High resolution mass spectra (HRMS) were obtained via

electrospray ionization (ESI) which was measured on a Thermo Scientific Q Exactive™ Plus Hybrid Quadrupole-Orbitrap™ at the UW Mass Spectrometry Facility.

2. Synthetic Procedures

2.1 Synthesis of Bithiazoles

2,2'-bithiazole (1a)

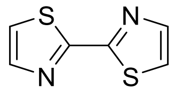

In a sealed, argon-purged pressure vessel, thiazole (710 μL, 10.0 mmol, 1.0 equiv) and $Cu(OAc)_2$ (363 mg, 2.0 mmol, 0.2 equiv) were dissolved in xylenes (33 mL, 0.3 M) and allowed to stir at 140 °C for 16 h in a sealed pressure vial. The reaction was then cooled to room temperature and depressurized in air for 10 min before being resealed and stirred at 140 °C for 16h. The reaction was then concentrated and purified by column chromatography (0 % – 10 % gradient EtOAc in Hexanes) to yield off-white solid **1a** (540 mg, 64%); R_f = 0.38 (EtOAc : Hexanes = 1 : 4); ^{1}H-NMR ($CDCl_3$, 300 MHz) δ 7.92 (d, *J* = 3.2 Hz, 2H), 7.46 (d, *J* = 3.2 Hz, 2H; ^{13}C-NMR ($CDCl_3$, 75 MHz) δ 161.5, 143.8, 121.0; Data consistent with previously reported literature.[1]

[2,2'-bithiazole] 3-oxide (1b)

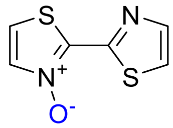

In a sealed, argon-purged microwave reaction vessel, **S2** (283 mg, 1.0 mmol, 1.0 equiv) was dissolved in anhydrous tetrahydrofuran (20 mL, 0.05 M) and allowed to stir at room temperature. *t*-BuOH (1.0 mL, 1 M) and LiO*t*-Bu (1.5 mL, 1 M in THF, 1.5 equiv) were then added and the mixture was immediately heated by microwave irradiation for 1 h at 60 °C. The reaction mixture was then diluted with dichloromethane (30 mL) and washed with water (3 x 20 mL) before being dried with $MgSO_4$ and concentrated. The reaction mixture was then concentrated and isolated by column chromatography (0 % – 10 % gradient MeOH in EtOAc) to yield off-white

solid **1b** (52 mg, 56%); R_f = 0.47 (EtOAc : MeOH = 1 : 9); ^{1}H-NMR ($CDCl_3$, 300 MHz) δ 8.03 (d, *J* = 2.7 Hz, 1H), 7.80 (d, *J* = 3.8 Hz, 1H), 7.56 (d, *J* = 2.8 Hz, 1H), 7.43 (d, *J* = 3.7 Hz, 1H); ^{13}C-NMR ($CDCl_3$, 75 MHz) δ 152.7, 143.4, 137.1, 120.9, 117.4; HRMS calculated for $C_6H_5ON_2S_2$ (M+H): 184.98378; Found: 184.98504 m/z.

[2,2'-bithiazole] 3,3'-dioxide (1c)

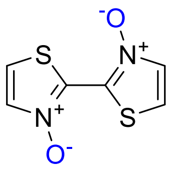

In a round bottom flask, **1b** (41 mg, 0.22 mmol, 1.0 equiv) was dissolved in 1,2-dichloroethane (1 mL, 0.3 M) and allowed to stir at room temperature. *m*-CPBA (188 mg, 0.84 mmol, 3.0 equiv, 77% pure) was then added and the mixture stirred for 6 h. The reaction mixture was then diluted with dichloromethane (3 mL), additional *m*-CPBA (188 mg, 0.84 mmol, 3.0 equiv, 77% pure) was added and the reaction mixture was continued to stir for 6 h. The reaction mixture was then concentrated and isolated by column chromatography (0 % – 15 % gradient MeOH in EtOAc) to yield off-white solid **1c** (22 mg, 50%); R_f = 0.02 (EtOAc); ^{1}H-NMR (DMSO-d_6, 300 MHz) δ 8.15 (d, *J* = 3.9 Hz, 2H), 7.98 (d, *J* = 3.9 Hz, 2H); ^{13}C-NMR (DMSO-d_6, 75 MHz) δ 136.0, 133.3, 120.8; HRMS (APCI) calculated for $C_6H_5O_2N_2S_2$ (M+H): 200.97870; Found: 200.97832 m/z.

4,4'-dimethyl-2,2'-bithiazole (1d)

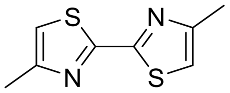

In a round bottom flask charged with 4-methylthiazole (920 µL, 10.1 mmol, 1.0 equiv) and $Cu(OAc)_2$ (405 mg, 2.0 mmol, 0.2 equiv) was added xylenes (33 mL, 0.3 M) and the mixture was stirred at reflux for 14 h. The reaction mixture was then cooled to room temperature, concentrated under vacuum, and purified by column chromatography (0 % – 20 % gradient EtOAc in Hexanes) to yield white solid **1d** (660

mg, 67%); R_f = 0.44 (EtOAc : Hexanes = 1 : 4); ^{1}H-NMR ($CDCl_3$, 300 MHz) δ 6.94 (s, 2H), 2.48 (s, 2H); ^{13}C-NMR ($CDCl_3$, 75 MHz) δ 160.8, 154.1, 115.4, 17.1; Data consistent with previously reported literature.[2]

4,4'-dimethyl-[2,2'-bithiazole] 3-oxide (1e)

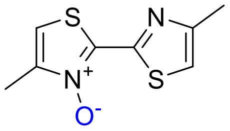

In a sealed, argon-purged microwave reaction vessel, **S3** (12 mg, 0.10 mmol, 1.0 equiv) was dissolved in anhydrous tetrahydrofuran (0.4 mL, 0.25 M) and allowed to stir in an ice bath. LiO*t*-Bu (150 µL, 1 M in THF, 1.5 equiv) was then added dropwise mixture was stirred for 15 min. The reaction mixture was then diluted with dichloromethane (10 mL) and sat. NH_4Cl (10 mL) and the aqueous layer was extracted with dichloromethane (10 mL). The organic layers were then combined, dried with $MgSO_4$ and concentrated to yield off-white solid **1e** (8 mg, 72%); R_f = 0.52 (MeOH : EtOAc = 1 : 9); ^{1}H-NMR ($CDCl_3$, 300 MHz) δ 7.10 (s, 1H), 7.09 (s, 1H), 2.54 (s, 3H), 2.45 (s, 1H); ^{13}C-NMR ($CDCl_3$, 75 MHz) δ 153.6, 152.6, 145.4, 140.1, 115.8, 111.7, 17.2, 12.6; Data consistent with previously reported literature.[3]

4,4'-dimethyl-[2,2'-bithiazole] 3,3'-dioxide (1f)

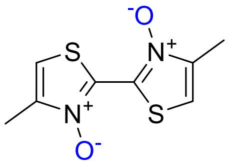

In a sealed, argon-purged microwave reaction vessel, **1d** (50 mg, 0.25 mmol, 1.0 equiv) was dissolved in 1,2-dichloroethane (0.5 mL, 0.5 M) and allowed to stir at room temperature. *m*-CPBA (168 mg, 0.75 mmol, 3.0 equiv, 77% pure) was then added and the mixture was continued stirring at this temperature for 6 h. The reaction mixture was then diluted with dichloromethane (2 mL) until the precipitate was fully dissolved, and a second addition of *m*-CPBA (168 mg, 0.75 mmol, 3.0 equiv, 77% pure) was performed and the mixture was stirred for 6 h. The product was then

filtered, and the filtrate was purified by column chromatography (0 % – 15 % gradient MeOH in EtOAc) to afford pale orange solid **1f** (42 mg, 65%); R_f = 0.36 (EtOAc : MeOH = 9 : 1); ^{1}H-NMR ($CDCl_3$, 300 MHz) δ 7.19 (s, 2H), 2.50 (s, 6H); ^{13}C-NMR ($CDCl_3$, 75 MHz) δ 144.1, 133.7, 30.8, 12.1; HRMS calculated for $C_8H_9O_2N_2S_2$ (M+H): 229.0101; Found: 229.0101 m/z.

2.2 Synthesis of Extended Bithiazoles

5,5'-bis(4-hexylphenyl)-2,2'-bithiazole (2a)

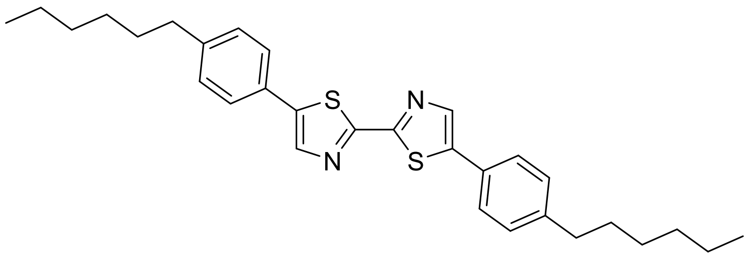

In a round bottom flask charged with **2c** (50 mg, 0.10 mmol, 1.0 equiv) was added tetrahydrofuran (1.5 mL, 0.07 M) and sat. NH_4Cl (1.5 mL, 0.07 M) stirred at room temperature. Zn (63 mg, 0.10 mmol, 10.0 equiv) was then added and the reaction stirred vigorously for 2 h. The reaction mixture extracted with diethyl ether (3 x 10 mL) whereupon the organic layers were collected, dried with $MgSO_4$ and concentrated to yield, without further purification, yellow solid **2a** (40 mg, 80%); R_f = 0.50 (EtOAc : Hexanes = 1 : 9); ^{1}H-NMR ($CDCl_3$, 300 MHz) δ 8.03 (s, 2H), 7.56 (d, J = 7.3 Hz, 4H), 7.27 (d, J = 7.4 Hz, 4H), 2.66 (t, J = 6.9 Hz, 4H), 1.66 (s, 4H), 1.35 – 1.29 (m, 12H), 0.92 (s, 6H); ^{13}C-NMR ($CDCl_3$, 75 MHz) δ 159.8, 144.1, 141.6, 139.0, 129.3, 128.2, 126.7, 35.7, 31.7, 31.3, 29.0, 22.6, 14.1 *two peaks missing due to overlap; HRMS calculated for $C_{30}H_{37}N_2S_2$ (M+H): 489.23927; Found: 489.23905 m/z.

5,5'-bis(4-hexylphenyl)-[2,2'-bithiazole] 3-oxide (2b)

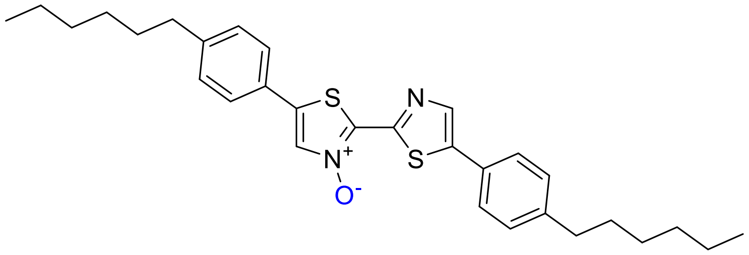

In a sealed, argon-purged microwave reaction vessel, **S5** (177 mg, 0.40 mmol, 1.0 equiv) was dissolved in anhydrous tetrahydrofuran (8 mL, 0.05 M) allowed to stir at room temperature. *t*-BuOH (0.4 mL, 1 M) and LiO*t*-Bu (0.6 mL, 1 M in THF, 1.5 equiv) were then added and the mixture was immediately heated by microwave irradiation for 1 h at 60 °C. The reaction mixture was then diluted with dichloromethane (30 mL) and washed with water (3 x 20 mL) before being dried with $MgSO_4$ and concentrated. The residue was then triturated using Hexanes (250 mL) to yield yellow solid **2b** (78 mg, 88%); R_f = 0.65 (EtOAc : Hexanes = 2 : 3); ^{1}H-NMR ($CDCl_3$, 300 MHz) δ 8.18 (s, 1H), 7.98 (s, 1H), 7.63 (d, *J* = 7.9 Hz, 2H), 7.52 (d, *J* = 8.0 Hz, 2H), 7.33 – 7.26 (m, 4H), 2.71 – 2.64 (m, 4H), 1.68 – 1.64 (m, 4H), 1.35 – 1.28 (m, 12H), 0.92 (t, *J* = 6.3 Hz, 6H); ^{13}C-NMR ($CDCl_3$, 75 MHz) δ 150.9, 146.0, 144.0, 140.8, 138.8, 135.9, 131.5, 129.6, 129.3, 128.4, 126.9, 126.2, 125.9, 35.8, 35.7, 31.7, 31.6, 31.3, 31.2, 29.0, 28.9, 22.6, 22.5, 14.1; HRMS calculated for $C_{30}H_{37}ON_2S_2$ (M+H): 505.23418; Found: 505.23242 m/z.

5,5'-bis(4-hexylphenyl)-[2,2'-bithiazole] 3,3'-dioxide (2c)

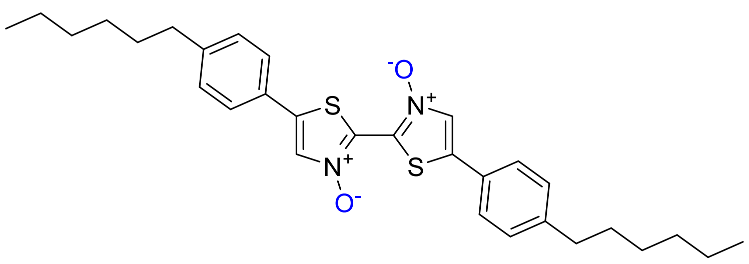

In a sealed, argon-purged microwave reaction vessel charged with **S5** (44 mg, 0.10 mmol, 1.0 equiv), $Cu(OAc)_2$ (9 mg, 0.05 mmol, 0.5 equiv) and K_2CO_3 (21 mg, 0.15 mmol, 1.5 equiv), was added in anhydrous tetrahydrofuran (0.5 mL, 0.1 M) and the

reaction stirred at 120 °C for 2h. The reaction was then allowed to cool, diluted with H_2O (10 mL) and extracted with dichloromethane (3 x 10 mL) before being dried with $MgSO_4$ and concentrated. The residue was then triturated using Hexanes (250 mL) to yield golden-yellow solid **2c** (17 mg, 66%); R_f = 0.20 (EtOAc : Hexanes = 2 : 3); ^{1}H-NMR ($CDCl_3$, 300 MHz) δ 8.06 (s, 2H), 7.56 (d, *J* = 7.8 Hz, 4H), 7.31 (d, *J* = 7.7 Hz, 4H), 2.68 (t, *J* = 7.6 Hz, 4H), 1.68 – 1.64 (m, 4H), 1.34 – 1.28 (m, 12H), 0.91 (t, *J* = 6.4 Hz, 6H); ^{13}C-NMR ($CDCl_3$, 75 MHz) δ 145.7, 136.9, 130.2, 129.6, 129.3, 126.5, 126.1, 35.8, 31.7, 31.2, 28.9, 22.6, 14.1; HRMS (APCI) calculated for $C_{30}H_{37}O_2N_2S_2$ (M+H): 521.22910; Found: 521.23110 m/z.

5,5'-bis(5-hexylthiophen-2-yl)-4,4'-dimethyl-2,2'-bithiazole (3a)

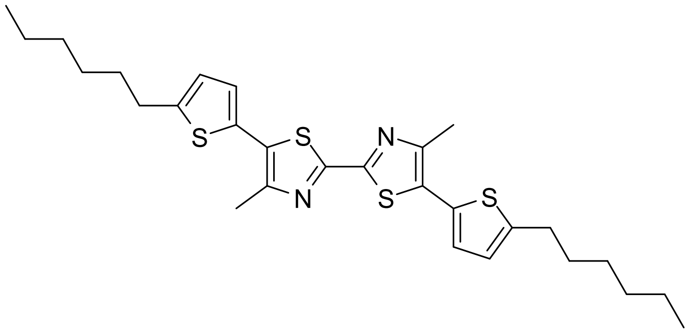

To a round bottom flask charged with $Pd(OAc)_2$ (7 mg, 4 mol %), K_2CO_3 (334 mg, 2.42 mmol, 3.0 equiv), PCy_3HBF_4 (24 mg, 8 mol %), and PivOH (49 mg, 60 mol %), was added DMA (6.5 mL, 0.12 M) followed by **1d** (158 mg, 0.81 mmol, 1.0 equiv) and 5-bromo-2-hexylthiophene (323 µL, 1.61 mmol, 2.0 equiv). The vial was sealed, purged with argon and the mixture stirred at 95 °C for 16 h. The reaction mixture was then diluted in Et_2O (15 mL) and washed with H_2O (3 x 15 mL) and brine (15 mL). The organic layer was then dried with $MgSO_4$ and filtered through Celite®. The filtrate was then concentrated and purified by column chromatography (0 % – 5 % gradient EtOAc in Hexanes) to afford orange solid **3a** (252 mg, 60%); R_f = 0.8 (EtOAc : Hexanes = 1 : 4); ^{1}H-NMR ($CDCl_3$, 300 MHz) δ 7.04 (d, *J* = 3.3 Hz, 2H), 6.78 (d, *J* = 3.5 Hz, 2H), 2.85 (t, *J* = 7.5 Hz, 4H), 2.64 (s, 6H), 1.72 (p, *J* = 7.0 Hz, 4H), 1.44 – 1.34 (m, 12H), 0.92 (t, *J* = 6.1 Hz, 6H); ^{13}C-NMR ($CDCl_3$, 75 MHz) δ 156.5, 148.7, 147.4, 130.4, 128.4, 126.8, 124.7, 31.4, 30.0, 28.6, 22.4, 16.7, 13.9; HRMS calculated for

$C_{28}H_{37}N_2S_4$ (M+H): 529.18341; Found: 529.18330 m/z.

5,5'-bis(5-hexylthiophen-2-yl)-4,4'-dimethyl-[2,2'-bithiazole] 3-oxide (3b)

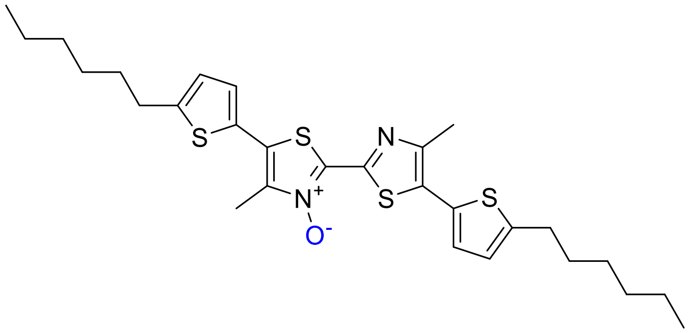

In a sealed, argon-purged microwave reaction vessel, **S7** (28 mg, 0.10 mmol, 1.0 equiv) was dissolved in anhydrous tetrahydrofuran (0.4 mL, 0.25 M) and allowed to stir in an ice bath. LiO*t*-Bu (150 µL, 1 M in THF, 1.5 equiv) was then added dropwise mixture was stirred for 15 min. The reaction mixture was then diluted with dichloromethane (10 mL) and sat. NH_4Cl (10 mL) and the aqueous layer was extracted with dichloromethane (10 mL). The organic layers were then combined, dried with $MgSO_4$ and concentrated to yield orange solid **3b** (15 mg, 56%); R_f = 0.62 (EtOAc : Hexanes = 2 : 3); ^{1}H-NMR ($CDCl_3$, 300 MHz) δ 7.16 (d, J = 3.6 Hz, 1H), 7.10 (d, J = 3.5 Hz, 1H), 6.84 (d, J = 3.5 Hz, 1H), 6.80 (d, J = 3.4 Hz, 1H), 2.88 – 2.84 (m, 4H), 2.68 (s, 3H), 2.64 (s, 3H), 1.73 (p, J = 6.9 Hz, 4H), 1.43 – 1.24 (m, 12H), 0.92 (t, J = 6.2 Hz, 6H); ^{13}C-NMR ($CDCl_3$, 75 MHz) δ 149.4, 148.5, 148.4, 147.7, 139.7, 137.8, 130.9, 129.2, 128.3, 127.4, 126.9, 125.2, 125.0, 123.8, 31.6, 31.5, 30.2, 30.1, 29.7, 28.8, 28.7, 22.6, 17.1, 14.1, 12.0; Data consistent with previously reported literature.[3]

5,5'-bis(5-hexylthiophen-2-yl)-4,4'-dimethyl-[2,2'-bithiazole] 3,3'-dioxide (3c)

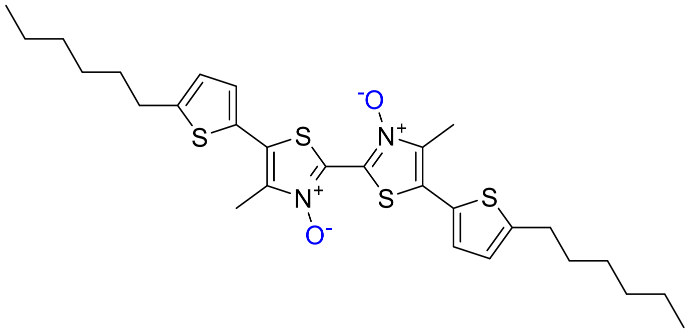

To a round bottom flask charged with $Pd(OAc)_2$ (4 mg, 4 mol %), Cs_2CO_3 (413 mg, 1.30 mmol, 3.0 equiv), tris(*O*-methoxytriphenyl)phosphine (12.4 mg, 8 mol %), and PivOH (45 mg, 0.44 mmol, 1.0 equiv), was added THF (1.5 mL, 0.3 M) followed by **1f** (100 mg, 0.44 mmol, 1.0 equiv) and 5-bromo-2-hexylthiophene (168 μL, 0.88 mmol, 2.0 equiv). The vial was sealed, purged with argon and the mixture stirred at 90 ℃ for 3 h. The reaction mixture was then diluted in EtOAc (15 mL) and washed with H_2O (3 x 15 mL) and brine (15 mL). The organic layer was then dried with $MgSO_4$ and filtered through Celite®. The filtrate was then concentrated and purified by column chromatography (30 % – 70 % gradient EtOAc in Hexanes) to afford dark orange solid **3c** (11 mg, 4%); R_f = 0.5 (EtOAc : Hexanes = 1 : 1); ^{1}H-NMR ($CDCl_3$, 300 MHz) δ 7.20 (d, *J* = 2.6 Hz, 2H), 6.85 (d, *J* = 3.2 Hz, 2H), 2.88 (t, *J* = 7.5 Hz, 4H), 2.66 (s, 6H) 1.74 (p, *J* = 7.1 Hz, 4H), 1.43 – 1.36 (m, 12H), 0.92 (t, *J* = 6.1 Hz, 6H); ^{13}C-NMR ($CDCl_3$, 75 MHz) δ 149.3, 138.8, 131.9, 129.3, 127.2, 125.3, 125.2, 31.4, 30.1, 28.6, 22.4, 13.9, 11.5; HRMS calculated for $C_{28}H_{37}N_2S_4O_2$ (M+H): 561.17324; Found: 561.17338 m/z.

2.3 Synthesis of Bithiazole Polymers

Fluorene Polymer (P1a)

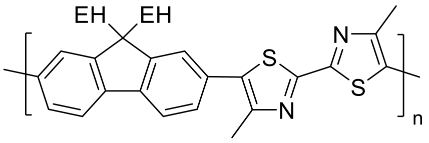

EH = 2-ethylhexyl

To a round bottom flask charged with $Pd(OAc)_2$ (2.3 mg, 4 mol %), Cs_2CO_3 (253 mg, 0.45 mmol, 3.0 equiv), tris(o-methoxyphenyl)phosphine (7 mg, 8 mol %), PivOH (26 mg, 0.25 mmol, 1.0 equiv), 9,9-Di-(2'-ethylhexyl)-2,7-dibromofluorene (125 mg, 0.25 mmol, 1.0 equiv) and **1d** (50 mg, 0.25 mmol, 1.0 equiv) was added anhydrous toluene (1.0 mL, 0.25 M). The vial was sealed, purged with argon and the mixture stirred at 90 ℃ for 48 h. The reaction mixture was then cooled and added dropwise to stirring methanol (20 mL) and the precipitate was collected by suction filtration. No further purification was required to yield polymer **P1a** (139 mg, quant.); ^{1}H-NMR ($CDCl_3$, 300

MHz) δ 7.84 (d, *J* = 7.7 Hz, 2H), 7.56 – 7.54 (m, 4H), 2.66 (s, 6H), 2.07 (s, 4H), 0.90 – 0.82 (m, 16H), 0.62 – 0.58 (m, 14H).

Fluorene Polymer (P1b)

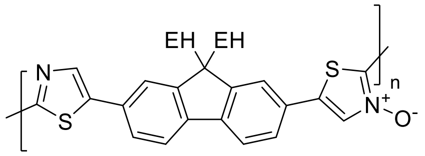

EH = 2-ethylhexyl

In a sealed, argon-purged microwave reaction vessel, **S9** (123 mg, 0.20 mmol, 1.0 equiv) was dissolved in anhydrous tetrahydrofuran (7 mL, 0.03 M) and allowed to stir in an ice bath. LiO*t*-Bu (300 µL, 1 M in THF, 1.5 equiv) was then added dropwise mixture was stirred for 3 h as it warmed to room temperature. The reaction mixture was then added dropwise to stirring methanol (20 mL) and the precipitate was collected by suction filtration as polymer **P1b** (119 mg, quant.); ^{1}H-NMR ($CDCl_3$, 300 MHz) δ7.84 (s, 2H), 7.59 (s, 4H), 2.66 (d, *J* = 12.0 Hz, 6H), 2.02 (s, 4H), 0.87 – 0.78 (m, 16H), 0.62 (s, 6H), 0.56 (s, 8H).

Fluorene Polymer (P1c)

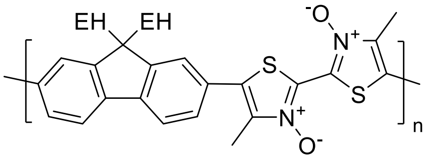

EH = 2-ethylhexyl

To a round bottom flask charged with $Pd(OAc)_2$ (1.0 mg, 4 mol %), Cs_2CO_3 (186 mg, 0.33 mmol, 3.0 equiv), tris(o-methoxyphenyl)phosphine (3 mg, 8 mol %), PivOH (11 mg, 0.11 mmol, 1.0 equiv), 9,9-Di-(2'-ethylhexyl)-2,7-dibromofluorene (55 mg, 0.11 mmol, 1.0 equiv) and **1f** (25 mg, 0.11 mmol, 1.0 equiv) was added anhydrous tetrahydrofuran (0.5 mL, 0.22 M). The vial was sealed, purged with argon and the mixture stirred at 90 ℃ for 48 h. The reaction mixture was then cooled and added dropwise to stirring methanol (20 mL) and the precipitate was collected by suction filtration. A Soxhlet extraction with acetone performed for 16 h yielded polymer **P1c** (25 mg, 39 %); ^{1}H-NMR ($CDCl_3$, 300 MHz) δ 7.93 (d, *J* = 7.3 Hz, 2H), 7.64 – 7.61 (m,

4H), 2.69 (s, 6H), 2.12 (s, 4H), 0.90 – 0.82 (m, 16H), 0.67 – 0.57 (m, 14H).

Thiophene Polymer (P2a)

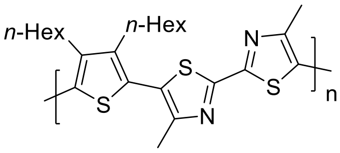

To a round bottom flask charged with $Pd(OAc)_2$ (1.4 mg, 4 mol %), Cs_2CO_3 (150 mg, 0.45 mmol, 3.0 equiv), tris(o-methoxyphenyl)phosphine (4 mg, 8 mol %), PivOH (16 mg, 0.15 mmol, 1.0 equiv) and **1d** (30 mg, 0.15 mmol, 1.0 equiv) was added anhydrous tetrahydrofuran (1.0 mL, 0.15 M) followed by 2,5-dibromo-3,4-dihexylthiophene (48 μL, 0.13 mmol, 1.0 equiv). The vial was sealed, purged with argon and the mixture stirred at 90 ℃ for 24 h. The reaction mixture was then cooled and added dropwise to stirring methanol (20 mL) and the precipitate was collected by suction filtration. No further purification was required to yield polymer **P2a** (63 mg, quant.); ^{1}H-NMR ($CDCl_3$, 300 MHz) δ 2.63 – 2.60 (m, 4H), 2.50 (s, 6H), 1.54 – 1.42 (m, 4H), 1.35 – 1.27 (m, 12H), 0.90 – 0.87 (m, 6H).

Thiophene Polymer (P2b)

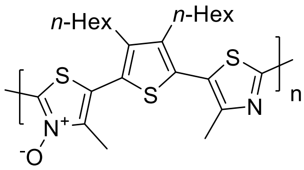

In a sealed, argon-purged microwave reaction vessel, **S11** (87 mg, 0.20 mmol, 1.0 equiv) was dissolved in anhydrous tetrahydrofuran (7 mL, 0.03 M) and allowed to stir in an ice bath. LiO*t*-Bu (300 μL, 1 M in THF, 1.5 equiv) was then added dropwise mixture was stirred for 3 h as it warmed to room temperature. The reaction mixture was then added dropwise to stirring methanol (20 mL) and the precipitate was collected by suction filtration as polymer **P2b** (86 mg, quant.); ^{1}H-NMR ($CDCl_3$, 300 MHz) δ 2.77 (br s, 3H), 2.60 (br s, 3H), 1.79 – 1.72 (m, 4H), 1.39 -1.22 (m, 16H), 0.90 – 0.86 (m, 6H).

Thiophene Polymer (P2c)

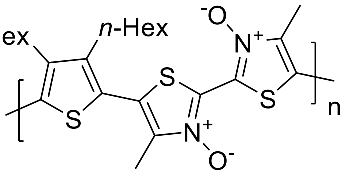

To a round bottom flask charged with $Pd(OAc)_2$ (2.5 mg, 8 mol %), Cs_2CO_3 (128 mg, 0.39 mmol, 3.0 equiv), tris(o-methoxyphenyl)phosphine (7 mg, 16 mol %), PivOH (13 mg, 0.13 mmol, 1.0 equiv) and **1f** (30 mg, 0.13 mmol, 1.0 equiv) was added to anhydrous tetrahydrofuran (1.0 mL, 0.13 M) followed by the addition of 2,5-dibromo-3,4-dihexylthiophene (41 μL, 0.13 mmol, 1.0 equiv). The vial was sealed, purged with argon and the mixture stirred at 90 ℃ for 24 h. A second addition of $Pd(OAc)_2$ (2.5 mg, 8 mol %) and tris(o-methoxyphenyl)phosphine (7 mg, 16 mol %) was performed and the reaction was continued to stir for 42 h at 90 °C. The reaction mixture was then cooled and added dropwise to stirring methanol (20 mL) and the precipitate was collected by suction filtration. The polymer was then purified by Soxhlet extraction with acetone for 16 h to yield polymer **P2c** (45 mg, 74%); ^{1}H-NMR ($CDCl_3$, 300 MHz) δ 2.64 – 2.57 (m, 4H), 2.49 (s, 6H), 1.48 – 1.40 (m, 4H), 1.30 – 1.18 (m, 12H), 0.91 – 0.84 (m, 6H).

2.4 Synthesis of Supplementary Compounds

diphenyl(thiazol-2-yl)methanol (S1)

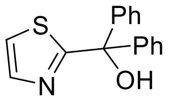

To a round-bottom flask charged with thiazole (1.42 mL, 20.0 mmol, 1.0 equiv), purged with argon and sealed was added THF (200 mL, 0.1 M) and allowed to stir in a -78 °C bath of dry ice and acetone. *n*BuLi (15 mL, 24.0 mmol, 1.6 M, 1.2 equiv) was added and the mixture was stirred at this temperature for 45 min. Benzophenone (5.10 g, 28.0 mmol, 1.4 equiv) was then added, the vessel purged with argon, and continued to stir at this temperature for 10 h. The reaction mixture was then quenched with NH_4Cl (75 mL) and extracted with diethyl ether (3 x 50 mL). The organic layer

was then dried with $MgSO_4$ and concentrated. The crude product was then purified by column chromatography (0 % – 20 % gradient EtOAc in Hexanes) to yield off-white solid **S1** (4.29 g, 79%); R_f = 0.27 (EtOAc : Hexanes = 1 : 4); ^{1}H-NMR ($CDCl_3$, 300 MHz) δ 7.72 (d, *J* = 3.1 Hz, 1H), 7.47 – 7.44 (m, 4H), 7.37 – 7.35 (m, 6H), 7.27 (d, *J* = 3.1 Hz, 1H), 5.06 (br s, 1H); ^{13}C-NMR ($CDCl_3$, 75 MHz) δ 177.7, 145.5, 142.7, 128.2, 128.0, 127.6, 120.0, 80.7; HRMS calculated for $C_{16}H_{14}ONS$ (M+H): 268.07906; Found: 368.07983 m/z.

2-(hydroxydiphenylmethyl)thiazole 3-oxide (S2)

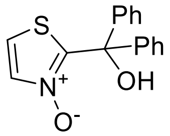

In a round-bottom flask, **S1** (4.2 g, 15.7 mmol, 1.0 equiv) was dissolved in 1,2-dichloroethane (50 mL, 0.3 M) allowed to stir at room temperature. *m*-CPBA (5.3 g, 23.6 mmol, 1.5 equiv, 77% pure) was then added and the mixture was continued stirring at this temperature for 6 h. The reaction mixture was then concentrated under vacuum and purified by column chromatography (10 % – 100 % gradient EtOAc in Hexanes) to afford white solid **S2** (3.87 g, 86%); R_f = 0.33 (EtOAc); ^{1}H-NMR ($CDCl_3$, 300 MHz) δ 8.38 (br s, 1H), 7.58 (d, *J* = 4.0 Hz, 1H), 7.35 – 7.33 (m, 10H), 7.20 (d, *J* = 4.0 Hz, 1H); ^{13}C-NMR ($CDCl_3$, 75 MHz) δ 151.7, 143.2, 137.7, 128.5, 128.4, 127.0, 117.8, 79.0; HRMS calculated for $C_{16}H_{14}O_2NS$ (M+H): 284.07398; Found: 284.07382 m/z.

4-methylthiazole 3-oxide (S3)

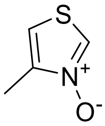

In a round-bottom flask, 4-methylthiazole (250 mg, 2.5 mmol, 1.0 equiv) was dissolved in 1,2-dichloroethane (8.3 mL, 0.3 M) and allowed to stir at room temperature. *m*-CPBA (802 mg, 3.8 mmol, 1.5 equiv, 77% pure) was then added and

the mixture was continued stirring at this temperature for 3 h whereupon precipitate is formed. The reaction mixture was then filtered and the filtrate was purified by column chromatography (0 % – 10 % gradient MeOH in EtOAc) to afford off-white solid **S3** (146 mg, 50%); R_f = 0.05 (EtOAc : Hexanes = 1 : 1); ^{1}H-NMR ($CDCl_3$, 300 MHz) δ 8.18 (d, *J* = 3.1 Hz, 1H), 7.04 (d, *J* = 2.1 Hz, 1H), 2.36 (s, 3H); ^{13}C-NMR ($CDCl_3$, 75 MHz) δ 145.6, 130.0, 113.4, 12.5; Data consistent with previously reported literature.[3]

(5-(4-hexylphenyl)thiazol-2-yl)diphenylmethanol (S4)

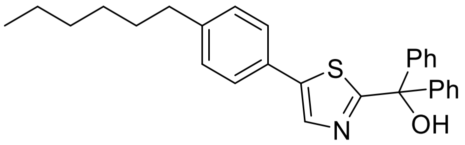

To a round-bottom flask charged with 5-(4-hexylphenyl)thiazole (2.45 g, 10.0 mmol, 1.0 equiv), purged with argon and sealed was added THF (100 mL, 0.1 M) and allowed to stir in a -78 °C bath of dry ice and acetone. *n*BuLi (7.5 mL, 12.0 mmol, 1.6 M, 1.2 equiv) was added and the mixture was stirred at this temperature for 30 min. Benzophenone (2.73 g, 15.0 mmol, 1.5 equiv) was then added, the vessel purged with argon, and continued to stir at this temperature for 4 h. The reaction mixture was then quenched with NH_4Cl (75 mL) and extracted with diethyl ether (3 x 50 mL). The organic layer was then dried with $MgSO_4$ and concentrated. The crude product was then purified by column chromatography (0 % – 10 % gradient EtOAc in Hexanes) to yield yellow oil **S4** (2.74 mg, 64%); R_f = 0.70 (EtOAc : Hexanes = 3 : 7); ^{1}H-NMR ($CDCl_3$, 300 MHz) δ 7.92 (s, 1H), 7.49 – 7.43 (m, 6H), 7.41 – 7.34 (m, 6H), 7.20 (d, *J* = 8.2 Hz, 2H), 4.32 (br s, 1H), 2.64 (t, *J* = 7.8 Hz, 2H), 1.64 (p, *J* = 7.5 Hz, 2H), 1.41 – 1.29 (m, 6H), 0.91 (t, *J* = 6.8 Hz, 3H); ^{13}C-NMR ($CDCl_3$, 75 MHz) δ 175.5, 145.3, 143.5, 140.8, 137.4, 129.1, 128.5, 128.2, 128.0, 127.5, 126.6, 80.7, 35.7, 31.7, 31.3, 28.9 22.6, 14.1; HRMS calculated for $C_{28}H_{30}ONS$ (M+H): 428.20426; Found: 428.20425 m/z.

5-(4-hexylphenyl)-2-(hydroxydiphenylmethyl)thiazole 3-oxide (S5)

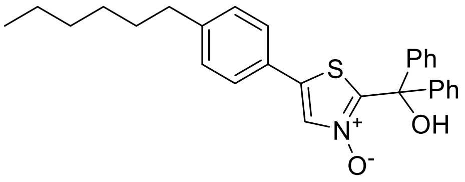

In a round-bottom flask, **S4** (1.28 g, 3.0 mmol, 1.0 equiv) was dissolved in 1,2-dichloroethane (9 mL, M) and allowed to stir at room temperature. *m*-CPBA (1.01 g, 4.5 mmol, 1.5 equiv, 77% pure) was then added and the mixture was continued stirring at this temperature for 6 h. The reaction mixture was then diluted with dichloromethane (30 mL) and then purified by column chromatography (0 % – 40 % gradient EtOAc in Hexanes) to afford white solid **S5** (1.07 g, 80%); R_f = 0.57 (EtOAc : Hexanes = 3 : 7); ^{1}H-NMR ($CDCl_3$, 300 MHz) δ 7.90 (s, 1H), 7.43 – 7.35 (m, 12H), 7.24 (d, *J* = 8.2 Hz, 2H), 2.64 (t, *J* = 7.9 Hz, 2H), 1.62 (p, *J* = 7.6 Hz, 2H), 1.32 – 1.31 (m, 6H), 0.90 (t, *J* = 6.6 Hz, 3H); ^{13}C-NMR ($CDCl_3$, 75 MHz) δ 150.6, 145.6, 143.2, 136.1, 132.2, 129.5, 129.5, 128.5, 128.4, 127.0, 126.0, 125.8, 79.1, 35.7, 31.7, 21.2, 28.9, 22.6, 14.1; HRMS calculated for $C_{28}H_{30}O_2NS$ (M+H): 444.19918; Found: 444.19903 m/z.

5-(5-hexylthiophen-2-yl)-4-methylthiazole (S6)

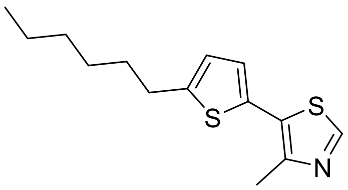

To a round bottom flask charged with $Pd(OAc)_2$ (4.5 mg, 4 mol %), Cs_2CO_3 (492 mg, 1.50 mmol, 3.0 equiv), tris(*O*-methoxytriphenyl)phosphine (14 mg, 8 mol %), and PivOH (52 mg, 0.50 mmol, 1.0 equiv), was added THF (1.8 mL, 0.3 M) followed by 4-methylthiazole (50 µL, 0.55 mmol, 1.1 equiv) and 5-bromo-2-hexylthiophene (101 µL, 0.50 mmol, 1.0 equiv). The vial was sealed, purged with argon and the mixture stirred at 90 ℃ for 3 h. The reaction mixture was then diluted in EtOAc (15 mL) and washed with H_2O (3 x 15 mL) and brine (15 mL). The organic layer was then dried with $MgSO_4$ and filtered through Celite®. The filtrate was then concentrated and

purified by column chromatography (0 % – 20 % gradient EtOAc in Hexanes) to afford amber liquid **S6** (70 mg, 53%); R_f = 0.77 (Hexanes); ^{1}H-NMR ($CDCl_3$, 300 MHz) δ 8.55 (s, 1H), 6.93 (d, *J* = 3.3 Hz, 1H), 6.73 (d, *J* = 2.9 Hz, 1H), 2.80 (t, *J* = 7.6 Hz, 2H), 2.60 (s, 3H), 1.69 (p, *J* = 7.4 Hz, 2H), 1.41 – 1.33 (m, 6H), 0.90 (t, *J* = 6.2 Hz, 3H); ^{13}C-NMR ($CDCl_3$, 75 MHz) δ 149.2, 148.2, 147.0, 130.4, 126.7, 126.0, 124.5, 31.4, 30.0, 28.7, 22.5, 16.4, 14.0 *one peak missing due to overlap; Data consistent with previously reported literature.[3]

5-(5-hexylthiophen-2-yl)-4-methylthiazole 3-oxide (S7)

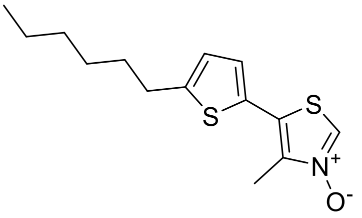

In a round-bottom flask, **S6** (70 mg, 0.28 mmol, 1.0 equiv) was dissolved in 1,2-dichloroethane (3 mL, 0.1 M) was allowed to stir at room temperature. *m*-CPBA (188 mg, 0.84 mmol, 3.0 equiv, 77% pure) was then added and the mixture was continued stirring at this temperature for 6 h. The reaction mixture was then diluted with dichloromethane (15 mL) and then purified by column chromatography (0 % – 10 % gradient EtOAc in Hexanes) to afford white solid **S7** (23 mg, 31%); R_f = 0.51 (EtOAc : Hexanes = 3 : 7); ^{1}H-NMR ($CDCl_3$, 300 MHz) δ 8.23 (s, 1H), 7.06 (d, *J* = 3.6 Hz, 1H), 6.80 (d, *J* = 3.6 Hz, 1H), 2.84 (t, *J* = 7.6 Hz, 2H), 2.53 (s, 3H), 1.70 (p, *J* = 7.5 Hz, 2H), 1.42 – 1.29 (m, 6H), 0.90 (t, *J* = 6.7 Hz, 3H); Data consistent with previously reported literature.[3]

5,5'-(9,9-bis(2-ethylhexyl)-9H-fluorene-2,7-diyl)bis(4-methylthiazole) (S8)

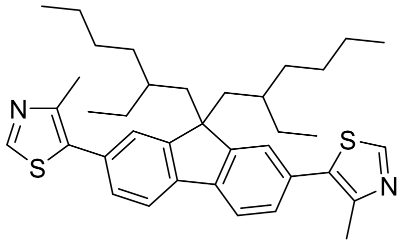

To a round bottom flask charged with $Pd(OAc)_2$ (22 mg, 4 mol %), K_2CO_3 (992 mg,

7.2 mmol, 3.0 equiv), PCy_3HBF_4 (72 mg, 8 mol %), and PivOH (176 mg, 1.7 mmol, 0.7 equiv), was added DMAc (10 mL, 0.25 M) followed by 4-methylthiazole (437 μL, 4.8 mmol, 2.0 equiv) and 9,9-(2-ethylhexyl)-2,7-dibromofluorene (1.32 g, 2.4 mmol, 1.0 equiv). The vial was sealed, purged with argon and the mixture stirred at 100 ℃ for 12 hours. The reaction mixture was then dissolved in H_2O (50 mL) and extracted with Et_2O (3 x 30 mL). The organic layer was then dried with $MgSO_4$ and filtered through Celite®. The filtrate was then concentrated and purified by column chromatography (0 % – 20 % gradient EtOAc in Hexanes) to afford yellow solid **S8** (1.11g, 78%); R_f = 0.39 (EtOAc : Hexanes = 1 : 4); ^{1}H-NMR ($CDCl_3$, 300 MHz) δ 8.68 (s, 2H), 7.74 (d, *J* = 7.7 Hz, 2H), 7.42 – 7.40 (m, 4H), 2.55 (s, 6H), 2.02 – 1.96 (m, 4H), 0.86 – 0.74 (m, 16H), 0.63 – 0.62 (m, 6H), 0.54 – 0.49 (m, 8H); ^{13}C-NMR ($CDCl_3$, 75 MHz) δ 151.2*, 150.2, 148.4, 140.5, 132.6, 130.4, 128.3*, 125.1*, 120.0, 55.3, 44.6, 34.8, 33.9*, 28.3*, 27.0*, 22.7, 16.1*, 13.9, 10.3* (* Splitting appears due to presence of diastereomers); Data consistent with previously reported.[3]

5,5'-(9,9-bis(2-ethylhexyl)-9H-fluorene-2,7-diyl)bis(4-methylthiazole 3-oxide) (S9)

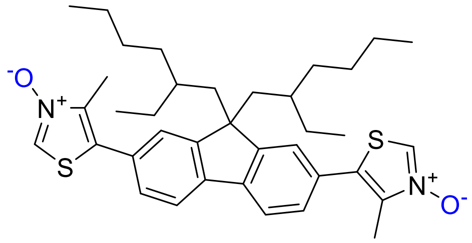

In a round-bottom flask, **S8** (509 mg, 0.87 mmol, 1.0 equiv) was dissolved in 1,2-dichloroethane (4 mL, 0.2 M) allowed to stir at room temperature. *m*-CPBA (584 mg, 2.60 mmol, 3.0 equiv, 77% pure) was then added and the mixture was continued stirring at this temperature for 2 h. An additional portion of *m*-CPBA (584 mg, 2.60 mmol, 3.0 equiv, 77% pure) was then added the reaction continued to stir for 6h. The reaction mixture was then diluted with dichloromethane (30 mL) and then purified by column chromatography (0 % – 10 % gradient MeOH in EtOAc) to afford white solid **S9** (148 mg, 28%); ^{1}H-NMR ($CDCl_3$, 300 MHz) δ 8.28 (s, 2H), 7.83 (d, *J* = 8.2 Hz, 2H), 7.44 (d, *J* = 6.1 Hz, 4H), 2.48 (s, 6H), 2.04 (d, *J* = 5.8 Hz, 4H), 0.84 – 0.74 (m, 16H),

0.63 (s, 6H), 0.53 – 0.50 (m, 8H); ^{13}C-NMR ($CDCl_3$, 75 MHz) δ 151.8*, 141.4, 141.3, 131.2, 129.3*, 129.3, 127.8*, 124.3, 120.8, 55.5, 44.3, 34.8, 33.9*, 28.2, 26.9*, 22.6, 13.8, 11.8*, 10.2* (* Splitting appears due to presence of diastereomers); Data consistent with previously reported literature.[3]

5,5'-(3,4-dihexylthiophene-2,5-diyl)bis(4-methylthiazole) (S10)

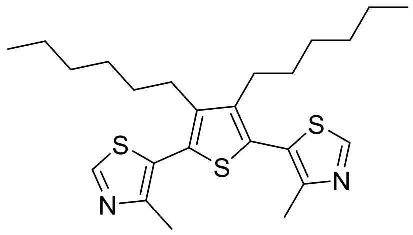

To a round bottom flask charged with $Pd(OAc)_2$ (45 mg, 4 mol %), K_2CO_3 (1.73 g, 12.5 mmol, 2.5 equiv), PCy_3HBF_4 (147 mg, 8 mol %), and PivOH (306 mg, 60 mol %), was added DMAc (15 mL, 0.33 M) followed by 4-methylthiazole (1.36 mL, 15.0 mmol, 3.0 equiv) and 2,5-dibromo-3,4-dihexylthiophene (1.56 mL, 5.0 mmol, 1.0 equiv). The vial was sealed, purged with argon and the mixture stirred at 100 ℃ for 12 hours. The reaction mixture was then dissolved in H_2O (50 mL) and extracted with Et_2O (3 x 30 mL). The organic layer was then dried with $MgSO_4$ and filtered through Celite®. The filtrate was then concentrated and purified by column chromatography (0 % – 20 % gradient EtOAc in Hexanes) to afford orange liquid **S10** (581 mg, 26%); ^{1}H-NMR ($CDCl_3$, 300 MHz) δ 8.80 (s, 2H), 2.52 - 2.45 (m, 10H), 1.45 – 1.24 (m, 16H), 0.87 (t, J = 6.6 Hz, 6H); ^{13}C-NMR ($CDCl_3$, 75 MHz) δ 152.3, 152.1, 143.1, 127.1, 123.4, 31.5, 30.5, 29.4, 28.1, 22.6, 16.1, 14.1; Data consistent with previously reported literature.[3]

5,5'-(3,4-dihexylthiophene-2,5-diyl)bis(4-methylthiazole 3-oxide) (S11)

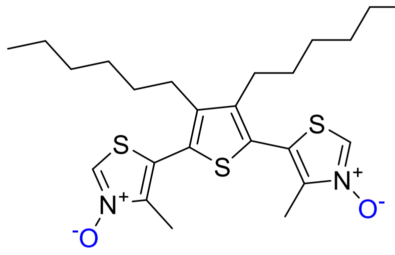

In a round-bottom flask, **S10** (560 mg, 1.25 mmol, 1.0 equiv) was dissolved in 1,2-dichloroethane (13 mL, 0.1 M) allowed to stir at room temperature. *m*-CPBA (840

mg, 3.75 mmol, 3.0 equiv, 77% pure) was then added and the mixture was continued stirring at this temperature for 6 h. The reaction mixture was then diluted with dichloromethane (30 mL) and then purified by column chromatography (0 % – 10 % gradient MeOH in EtOAc) to afford white solid **S10** (63 mg, 10%); ^{1}H-NMR ($CDCl_3$, 300 MHz) δ 8.37 (s, 2H), 2.53 (t, *J* = 8.4 Hz, 4H), 2.37 (s, 6H), 1.45 – 1.27 (m, 16H), 0.89 (t, *J* = 6.2 Hz, 6H); ^{13}C-NMR ($CDCl_3$, 75 MHz) δ144.6, 144.5, 130.1, 126.4, 122.2, 31.3, 30.6, 29.3, 27.9, 22.4, 13.9, 12.0; Data consistent with previously reported literature.[3]

3. Absorption Spectra

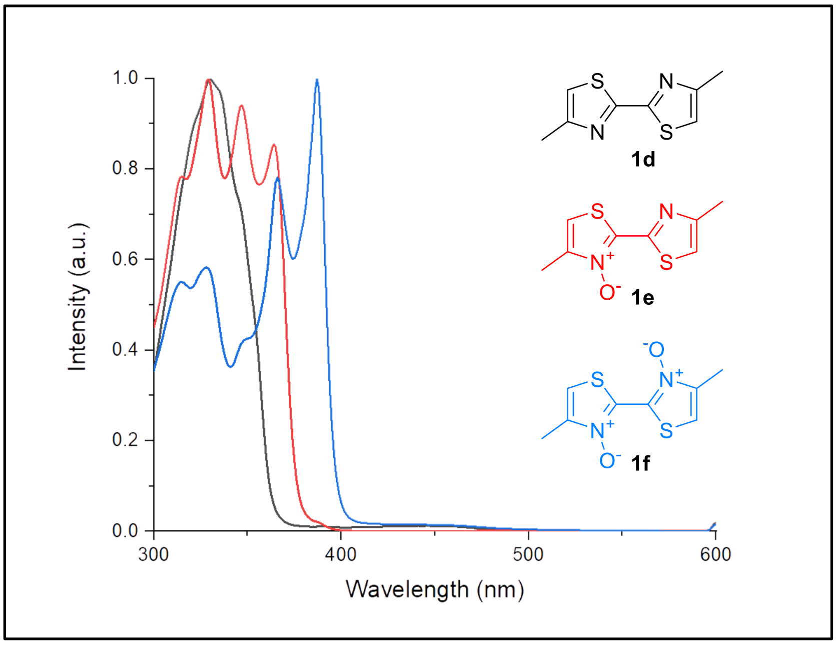

Figure S1 – UV/Vis absorption spectra for 4,4'-dimethyl-2,2'-bithiazole at various oxidation levels (**1d-f**). Absorption spectra measured in $CHCl_3$.

5. Thermal Data

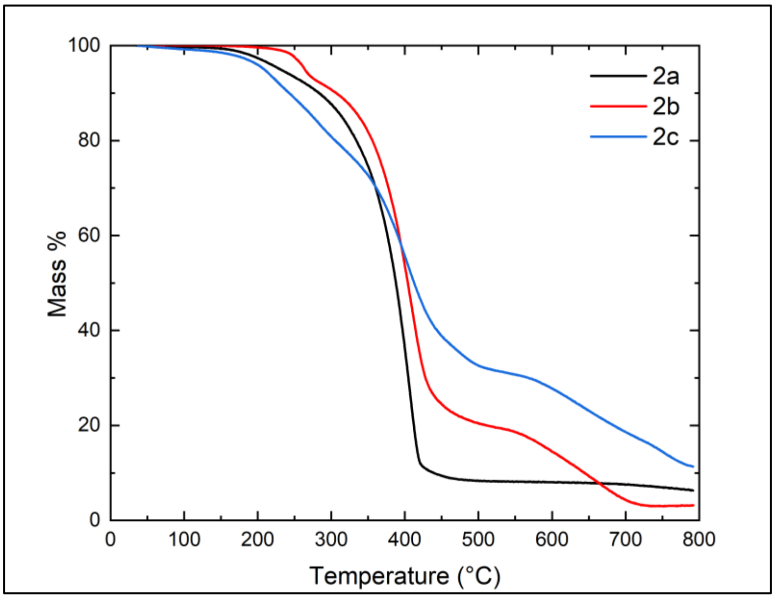

Figure S2 – Thermogravimetric analysis (TGA) of series **2**. Decomposition of compounds began at similar temperatures of 180 – 200 °C which were used as the upper limit in DSC experiments.

5. Voltammetry

Linear-sweep voltammetry was performed by drop casting polymer solution onto Pt button working electrode. Measured in MeCN with 0.1M nBu_4NPF_6 as the supporting electrolyte using a Pt counter electrode, and Pt reference electrode. Fc/Fc^+ used as an internal standard. Electrochemical bandgaps were determined by the difference between HOMO and LUMO levels. HOMO levels were estimated from first oxidation potential. LUMOs estimated form first reduction potential.

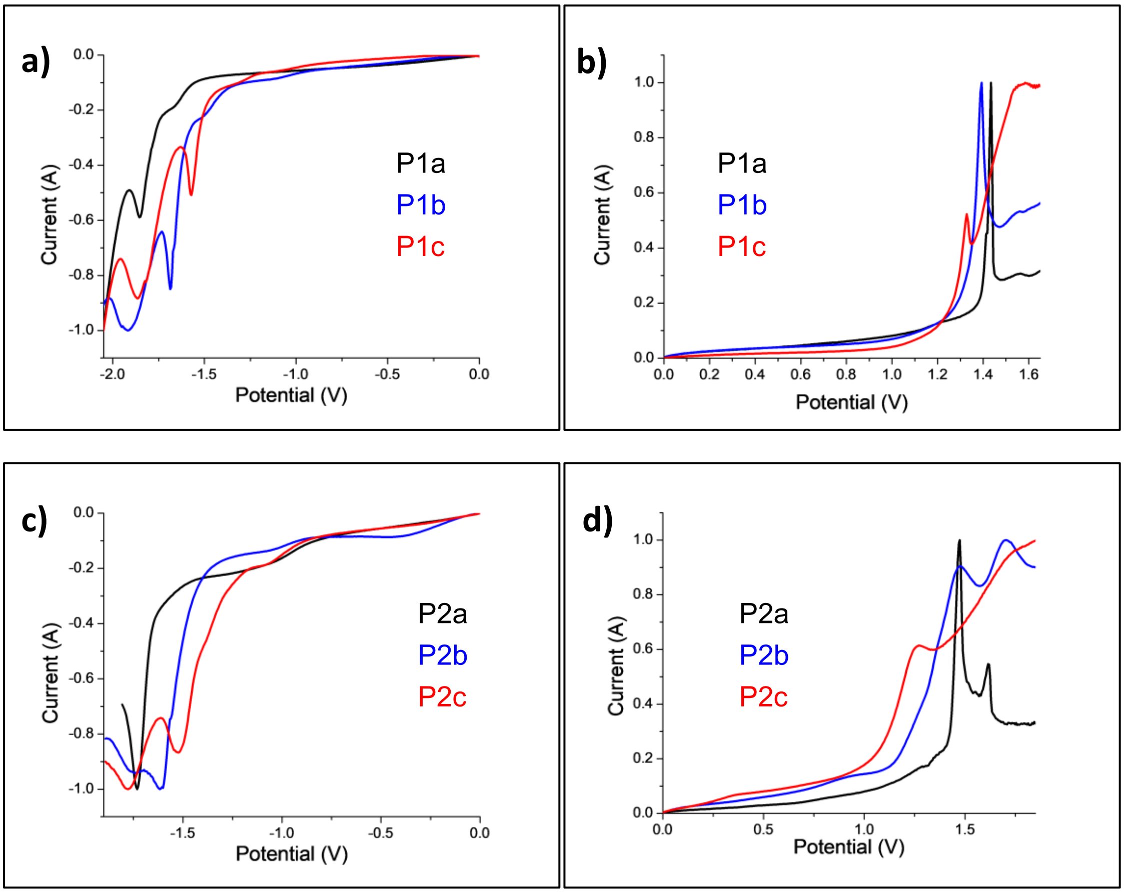

Figure S3 – Linear-sweep Voltammograms a) Series P1 reduction b) Series P1 oxidation c) Series P2 reduction d) Series P2 oxidation.

Table S1 – Summary of voltammetry results.

Entry	HOMO (eV)	LUMO (eV)	$Eg_{[ec]}$ (eV)
**P1a**	-5.75	-2.80	2.95
**P1b**	-5.69	-2.86	2.83
**P1c**	-5.65	-2.95	2.70
**P2a**	-5.67	-2.71	2.86
**P2b**	-5.57	-2.96	2.61
**P2c**	-5.53	-3.02	2.51

6. Gel Permeation Chromatography

Number-average (M_N) and weight-average (M_W) molecular weights were determined by size exclusion chromatography using a Viscotek GPCmax VE2001 at 35 °C equipped with a VE 3580 RI detector and two PAS-104 Styrene-Divinylbenzene gel columns. The flow rate was fixed at 1.0 mL/min using tetrahydrofuran (THF) as the eluent. All molecular weights are relative to a polystyrene calibration curve. All GPC samples were prepared nominally at 2 mg/mL in THF and filtered through a 0.22 μM PTFE filter into a 1 mL chromatography vial.

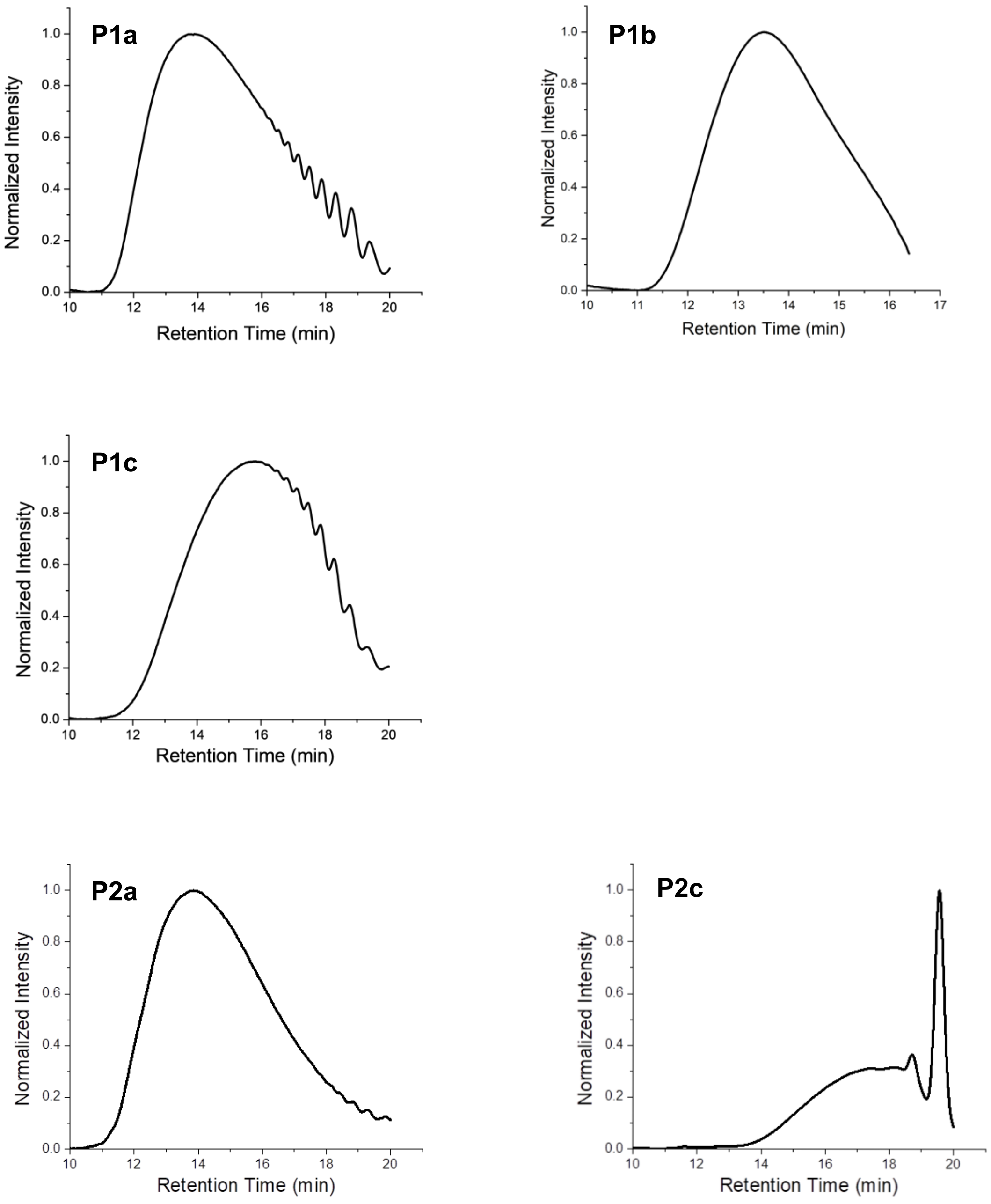

Figure S4 – GPC traces for **P1a-c** and **P2a**/c. Trace for **P2b** unavailable.

7. X-Ray Crystal Data Tables

Figure X1. Compound **2a** with packing and short contacts.

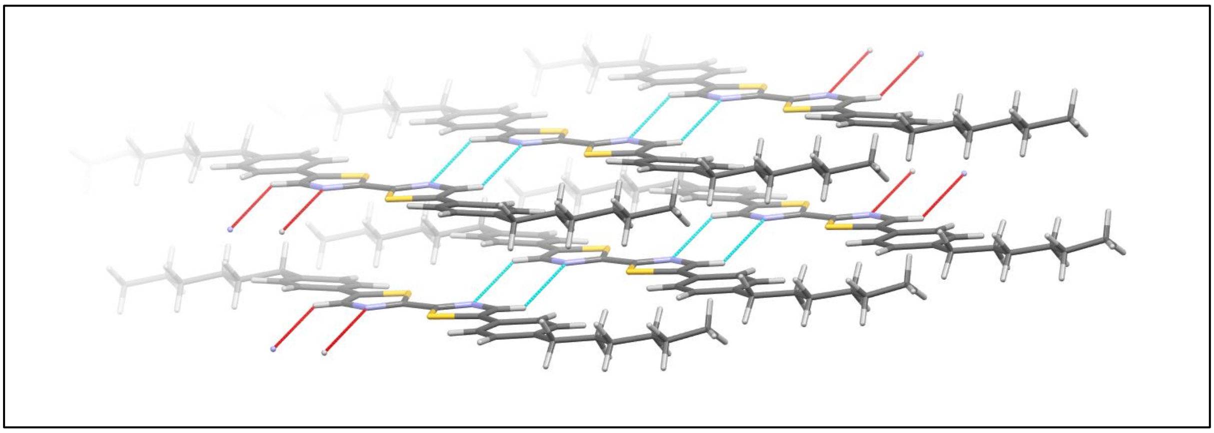

Table X1. Crystal data and structure refinement for (2a) $C_{30}H_{36}N_2S_2$

Empirical formula	$C_{30}H_{36}N_2S_2$
Formula weight	488.73
Temperature	296(2) K
Wavelength	0.71073 Å
Crystal system	Triclinic
Space group	*P*-1
Unit cell dimensions	*a* = 7.0096(4) Å, *b* = 11.6756(6) Å, *c* = 17.1032(9) Å
	a= 96.573(2)°, *b*= 98.931(2)°, *g* = 99.536(2)°.
Volume	1349.48(13) $Å^3$
Z	2
Density (calculated)	1.203 g/cm^3
Absorption coefficient	0.218 mm^{-1}
F(000)	524
Crystal size	0.520 x 0.130 x 0.020 mm^3
Theta range for data collection	1.787 to 27.998°.
Index ranges	-9<=h<=9, -15<=k<=15, -22<=l<=22
Reflections collected	24263
Independent reflections	6520 [R(int) = 0.0784]
Completeness to theta = 25.242°	99.9 %
Absorption correction	Semi-empirical from equivalents
Max. and min. transmission	0.7460 and 0.6663
Refinement method	Full-matrix least-squares on F^2
Data / restraints / parameters	6520 / 0 / 310
Goodness-of-fit on F^2	1.316

Final R indices [I>2sigma(I)]	R1 = 0.0549, wR2 = 0.0847
R indices (all data)	R1 = 0.1351, wR2 = 0.0969
Extinction coefficient	0.0006(3)
Largest diff. peak and hole	0.296 and -0.218 e.Å^{-3}

Table X2. Atomic coordinates (x 10^4) and equivalent isotropic displacement parameters (Å^{2}x 10^3) for $C_{30}H_{36}N_2S_2$

	x	y	z	U(eq)
S(1A)	5791(1)	8649(1)	5652(1)	50(1)
C(2A)	4654(3)	7465(2)	4951(1)	41(1)
N(3A)	2755(3)	7374(2)	4745(1)	53(1)
C(4A)	2160(3)	8284(2)	5154(1)	55(1)
C(5A)	3545(3)	9071(2)	5673(1)	42(1)
C(6A)	3322(3)	10117(2)	6186(1)	42(1)
C(7A)	1498(3)	10402(2)	6207(2)	59(1)
C(8A)	1272(3)	11391(2)	6688(2)	57(1)
C(9A)	2857(3)	12132(2)	7171(1)	46(1)
C(10A)	4689(3)	11850(2)	7141(1)	56(1)
C(11A)	4921(3)	10880(2)	6663(1)	54(1)
C(12A)	2685(3)	13207(2)	7720(1)	56(1)
C(13A)	658(3)	13474(2)	7741(1)	56(1)
C(14A)	702(3)	14549(2)	8337(2)	60(1)
C(15A)	-1274(4)	14802(2)	8456(2)	67(1)
C(16A)	-1184(4)	15865(2)	9053(2)	86(1)
C(17A)	-3102(5)	16055(3)	9270(2)	116(1)
S(1B)	4627(1)	5416(1)	3944(1)	48(1)
C(2B)	5756(3)	6636(2)	4626(1)	41(1)
N(3B)	7647(3)	6747(2)	4823(1)	51(1)
C(4B)	8284(3)	5845(2)	4416(1)	53(1)
C(5B)	6888(3)	5025(2)	3914(1)	42(1)
C(6B)	7155(3)	3985(2)	3403(1)	43(1)
C(7B)	8966(3)	3654(2)	3455(1)	60(1)
C(8B)	9237(4)	2684(2)	2971(2)	62(1)
C(9B)	7731(3)	2010(2)	2417(1)	46(1)
C(10B)	5928(3)	2343(2)	2368(2)	63(1)
C(11B)	5640(3)	3307(2)	2848(2)	59(1)

C(12B)	7946(3)	924(2)	1887(1)	58(1)
C(13B)	9987(4)	766(2)	1795(2)	58(1)
C(14B)	9994(3)	-332(2)	1226(1)	58(1)
C(15B)	11996(3)	-523(2)	1085(1)	62(1)
C(16B)	11943(4)	-1622(2)	520(2)	72(1)
C(17B)	13937(4)	-1836(3)	373(2)	101(1)
H(4A)	847	8360	5079	66
H(7A)	394	9920	5890	71
H(8A)	20	11560	6684	69
H(10A)	5793	12335	7455	67
H(11A)	6179	10726	6656	65
H(12A)	3267	13128	8259	68
H(12B)	3476	13883	7569	68
H(13A)	-165	12804	7883	67
H(13B)	78	13601	7213	67
H(14A)	1423	14452	8850	72
H(14B)	1425	15227	8162	72
H(15A)	-1994	14909	7946	81
H(15B)	-2003	14125	8630	81
H(16A)	-303	15805	9537	103
H(16B)	-612	16552	8841	103
H(17A)	-2917	16799	9603	175
H(17B)	-3594	15440	9556	175
H(17C)	-4030	16047	8793	175
H(4B)	9608	5796	4480	63
H(7B)	10025	4092	3823	71
H(8B)	10476	2483	3023	74
H(10B)	4871	1905	1998	75
H(11B)	4398	3503	2796	71
H(12C)	7331	244	2095	70
H(12D)	7204	922	1359	70
H(13C)	10730	720	2315	69
H(13D)	10636	1446	1598	69
H(14C)	9370	-1008	1435	70
H(14D)	9200	-294	715	70
H(15C)	12622	147	871	75
H(15D)	12796	-563	1594	75
H(16C)	11144	-1578	11	86

H(16D)	11305	-2290	733	86
H(17D)	13777	-2548	9	151
H(17E)	14731	-1903	870	151
H(17F)	14569	-1191	147	151

U(eq) is defined as one third of the trace of the orthogonalized U_{ij} tensor.

Table X3. Bond lengths [Å] and angles [°] for $C_{30}H_{36}N_2S_2$

S(1A)-C(2A)	1.715(2)
S(1A)-C(5A)	1.730(2)
C(2A)-N(3A)	1.306(2)
C(2A)-C(2B)	1.449(3)
N(3A)-C(4A)	1.363(3)
C(4A)-C(5A)	1.351(3)
C(4A)-H(4A)	0.9300
C(5A)-C(6A)	1.465(3)
C(6A)-C(7A)	1.378(3)
C(6A)-C(11A)	1.390(3)
C(7A)-C(8A)	1.385(3)
C(7A)-H(7A)	0.9300
C(8A)-C(9A)	1.377(3)
C(8A)-H(8A)	0.9300
C(9A)-C(10A)	1.384(3)
C(9A)-C(12A)	1.513(3)
C(10A)-C(11A)	1.368(3)
C(10A)-H(10A)	0.9300
C(11A)-H(11A)	0.9300
C(12A)-C(13A)	1.509(3)
C(12A)-H(12A)	0.9700
C(12A)-H(12B)	0.9700
C(13A)-C(14A)	1.516(3)
C(13A)-H(13A)	0.9700
C(13A)-H(13B)	0.9700
C(14A)-C(15A)	1.502(3)
C(14A)-H(14A)	0.9700
C(14A)-H(14B)	0.9700
C(15A)-C(16A)	1.500(3)

C(15A)-H(15A)	0.9700
C(15A)-H(15B)	0.9700
C(16A)-C(17A)	1.494(4)
C(16A)-H(16A)	0.9700
C(16A)-H(16B)	0.9700
C(17A)-H(17A)	0.9600
C(17A)-H(17B)	0.9600
C(17A)-H(17C)	0.9600
S(1B)-C(2B)	1.726(2)
S(1B)-C(5B)	1.727(2)
C(2B)-N(3B)	1.296(3)
N(3B)-C(4B)	1.370(3)
C(4B)-C(5B)	1.360(3)
C(4B)-H(4B)	0.9300
C(5B)-C(6B)	1.469(3)
C(6B)-C(11B)	1.376(3)
C(6B)-C(7B)	1.380(3)
C(7B)-C(8B)	1.382(3)
C(7B)-H(7B)	0.9300
C(8B)-C(9B)	1.370(3)
C(8B)-H(8B)	0.9300
C(9B)-C(10B)	1.376(3)
C(9B)-C(12B)	1.515(3)
C(10B)-C(11B)	1.377(3)
C(10B)-H(10B)	0.9300
C(11B)-H(11B)	0.9300
C(12B)-C(13B)	1.502(3)
C(12B)-H(12C)	0.9700
C(12B)-H(12D)	0.9700
C(13B)-C(14B)	1.520(3)
C(13B)-H(13C)	0.9700
C(13B)-H(13D)	0.9700
C(14B)-C(15B)	1.508(3)
C(14B)-H(14C)	0.9700
C(14B)-H(14D)	0.9700
C(15B)-C(16B)	1.507(3)
C(15B)-H(15C)	0.9700
C(15B)-H(15D)	0.9700

C(16B)-C(17B)	1.513(3)
C(16B)-H(16C)	0.9700
C(16B)-H(16D)	0.9700
C(17B)-H(17D)	0.9600
C(17B)-H(17E)	0.9600
C(17B)-H(17F)	0.9600
C(2A)-S(1A)-C(5A)	89.63(11)
N(3A)-C(2A)-C(2B)	123.65(18)
N(3A)-C(2A)-S(1A)	115.05(17)
C(2B)-C(2A)-S(1A)	121.30(15)
C(2A)-N(3A)-C(4A)	109.55(19)
C(5A)-C(4A)-N(3A)	117.7(2)
C(5A)-C(4A)-H(4A)	121.1
N(3A)-C(4A)-H(4A)	121.1
C(4A)-C(5A)-C(6A)	129.2(2)
C(4A)-C(5A)-S(1A)	108.07(17)
C(6A)-C(5A)-S(1A)	122.75(17)
C(7A)-C(6A)-C(11A)	116.7(2)
C(7A)-C(6A)-C(5A)	121.0(2)
C(11A)-C(6A)-C(5A)	122.3(2)
C(6A)-C(7A)-C(8A)	121.5(2)
C(6A)-C(7A)-H(7A)	119.3
C(8A)-C(7A)-H(7A)	119.3
C(9A)-C(8A)-C(7A)	121.6(2)
C(9A)-C(8A)-H(8A)	119.2
C(7A)-C(8A)-H(8A)	119.2
C(8A)-C(9A)-C(10A)	116.7(2)
C(8A)-C(9A)-C(12A)	123.7(2)
C(10A)-C(9A)-C(12A)	119.6(2)
C(11A)-C(10A)-C(9A)	121.9(2)
C(11A)-C(10A)-H(10A)	119.1
C(9A)-C(10A)-H(10A)	119.1
C(10A)-C(11A)-C(6A)	121.6(2)
C(10A)-C(11A)-H(11A)	119.2
C(6A)-C(11A)-H(11A)	119.2
C(13A)-C(12A)-C(9A)	117.90(19)
C(13A)-C(12A)-H(12A)	107.8

C(9A)-C(12A)-H(12A)	107.8
C(13A)-C(12A)-H(12B)	107.8
C(9A)-C(12A)-H(12B)	107.8
H(12A)-C(12A)-H(12B)	107.2
C(12A)-C(13A)-C(14A)	112.12(19)
C(12A)-C(13A)-H(13A)	109.2
C(14A)-C(13A)-H(13A)	109.2
C(12A)-C(13A)-H(13B)	109.2
C(14A)-C(13A)-H(13B)	109.2
H(13A)-C(13A)-H(13B)	107.9
C(15A)-C(14A)-C(13A)	115.3(2)
C(15A)-C(14A)-H(14A)	108.4
C(13A)-C(14A)-H(14A)	108.4
C(15A)-C(14A)-H(14B)	108.4
C(13A)-C(14A)-H(14B)	108.4
H(14A)-C(14A)-H(14B)	107.5
C(16A)-C(15A)-C(14A)	114.1(2)
C(16A)-C(15A)-H(15A)	108.7
C(14A)-C(15A)-H(15A)	108.7
C(16A)-C(15A)-H(15B)	108.7
C(14A)-C(15A)-H(15B)	108.7
H(15A)-C(15A)-H(15B)	107.6
C(17A)-C(16A)-C(15A)	115.8(3)
C(17A)-C(16A)-H(16A)	108.3
C(15A)-C(16A)-H(16A)	108.3
C(17A)-C(16A)-H(16B)	108.3
C(15A)-C(16A)-H(16B)	108.3
H(16A)-C(16A)-H(16B)	107.4
C(16A)-C(17A)-H(17A)	109.5
C(16A)-C(17A)-H(17B)	109.5
H(17A)-C(17A)-H(17B)	109.5
C(16A)-C(17A)-H(17C)	109.5
H(17A)-C(17A)-H(17C)	109.5
H(17B)-C(17A)-H(17C)	109.5
C(2B)-S(1B)-C(5B)	89.62(11)
N(3B)-C(2B)-C(2A)	123.13(18)
N(3B)-C(2B)-S(1B)	114.93(17)
C(2A)-C(2B)-S(1B)	121.93(15)

C(2B)-N(3B)-C(4B)	110.18(18)
C(5B)-C(4B)-N(3B)	116.9(2)
C(5B)-C(4B)-H(4B)	121.6
N(3B)-C(4B)-H(4B)	121.6
C(4B)-C(5B)-C(6B)	128.3(2)
C(4B)-C(5B)-S(1B)	108.36(16)
C(6B)-C(5B)-S(1B)	123.28(16)
C(11B)-C(6B)-C(7B)	116.9(2)
C(11B)-C(6B)-C(5B)	122.2(2)
C(7B)-C(6B)-C(5B)	120.9(2)
C(6B)-C(7B)-C(8B)	121.3(2)
C(6B)-C(7B)-H(7B)	119.3
C(8B)-C(7B)-H(7B)	119.3
C(9B)-C(8B)-C(7B)	121.7(2)
C(9B)-C(8B)-H(8B)	119.1
C(7B)-C(8B)-H(8B)	119.1
C(8B)-C(9B)-C(10B)	116.7(2)
C(8B)-C(9B)-C(12B)	123.6(2)
C(10B)-C(9B)-C(12B)	119.6(2)
C(9B)-C(10B)-C(11B)	122.0(2)
C(9B)-C(10B)-H(10B)	119.0
C(11B)-C(10B)-H(10B)	119.0
C(6B)-C(11B)-C(10B)	121.2(2)
C(6B)-C(11B)-H(11B)	119.4
C(10B)-C(11B)-H(11B)	119.4
C(13B)-C(12B)-C(9B)	117.76(19)
C(13B)-C(12B)-H(12C)	107.9
C(9B)-C(12B)-H(12C)	107.9
C(13B)-C(12B)-H(12D)	107.9
C(9B)-C(12B)-H(12D)	107.9
H(12C)-C(12B)-H(12D)	107.2
C(12B)-C(13B)-C(14B)	112.5(2)
C(12B)-C(13B)-H(13C)	109.1
C(14B)-C(13B)-H(13C)	109.1
C(12B)-C(13B)-H(13D)	109.1
C(14B)-C(13B)-H(13D)	109.1
H(13C)-C(13B)-H(13D)	107.8
C(15B)-C(14B)-C(13B)	115.2(2)

C(15B)-C(14B)-H(14C)	108.5
C(13B)-C(14B)-H(14C)	108.5
C(15B)-C(14B)-H(14D)	108.5
C(13B)-C(14B)-H(14D)	108.5
H(14C)-C(14B)-H(14D)	107.5
C(16B)-C(15B)-C(14B)	113.7(2)
C(16B)-C(15B)-H(15C)	108.8
C(14B)-C(15B)-H(15C)	108.8
C(16B)-C(15B)-H(15D)	108.8
C(14B)-C(15B)-H(15D)	108.8
H(15C)-C(15B)-H(15D)	107.7
C(15B)-C(16B)-C(17B)	114.6(2)
C(15B)-C(16B)-H(16C)	108.6
C(17B)-C(16B)-H(16C)	108.6
C(15B)-C(16B)-H(16D)	108.6
C(17B)-C(16B)-H(16D)	108.6
H(16C)-C(16B)-H(16D)	107.6
C(16B)-C(17B)-H(17D)	109.5
C(16B)-C(17B)-H(17E)	109.5
H(17D)-C(17B)-H(17E)	109.5
C(16B)-C(17B)-H(17F)	109.5
H(17D)-C(17B)-H(17F)	109.5
H(17E)-C(17B)-H(17F)	109.5

Symmetry transformations used to generate equivalent atoms:

Table X4. Anisotropic displacement parameters ($Å^2$ x 10^3) for $C_{30}H_{36}N_2S_2$

	U_{11}	U_{22}	U_{33}	U_{23}	U_{13}	U_{12}
S(1A)	37(1)	47(1)	61(1)	-6(1)	5(1)	7(1)
C(2A)	38(1)	38(1)	46(1)	2(1)	10(1)	5(1)
N(3A)	36(1)	46(1)	72(1)	-10(1)	7(1)	7(1)
C(4A)	38(1)	49(2)	76(2)	-6(1)	8(1)	9(1)
C(5A)	39(1)	39(1)	50(2)	5(1)	12(1)	7(1)
C(6A)	36(1)	41(1)	49(2)	5(1)	10(1)	6(1)
C(7A)	42(2)	57(2)	69(2)	-11(1)	1(1)	9(1)
C(8A)	41(1)	60(2)	71(2)	-4(1)	11(1)	18(1)

C(9A)	47(2)	42(1)	50(2)	5(1)	14(1)	11(1)
C(10A)	42(1)	49(2)	69(2)	-7(1)	7(1)	2(1)
C(11A)	36(1)	52(2)	71(2)	-5(1)	10(1)	7(1)
C(12A)	58(2)	48(2)	64(2)	0(1)	14(1)	13(1)
C(13A)	59(2)	49(2)	61(2)	1(1)	15(1)	14(1)
C(14A)	66(2)	49(2)	67(2)	-1(1)	20(1)	15(1)
C(15A)	74(2)	60(2)	71(2)	-1(2)	19(2)	22(1)
C(16A)	98(2)	67(2)	97(2)	-7(2)	29(2)	35(2)
C(17A)	114(3)	138(3)	109(3)	-13(2)	31(2)	69(2)
S(1B)	37(1)	46(1)	58(1)	-4(1)	6(1)	6(1)
C(2B)	39(1)	38(1)	45(2)	3(1)	8(1)	3(1)
N(3B)	38(1)	42(1)	67(1)	-8(1)	6(1)	5(1)
C(4B)	34(1)	53(2)	70(2)	-6(1)	10(1)	10(1)
C(5B)	37(1)	40(1)	48(1)	4(1)	9(1)	9(1)
C(6B)	41(1)	41(1)	46(2)	5(1)	9(1)	7(1)
C(7B)	45(2)	65(2)	62(2)	-16(1)	1(1)	16(1)
C(8B)	51(2)	67(2)	65(2)	-13(2)	4(1)	24(1)
C(9B)	50(2)	40(1)	50(2)	2(1)	14(1)	12(1)
C(10B)	47(2)	55(2)	76(2)	-16(1)	3(1)	4(1)
C(11B)	39(1)	53(2)	78(2)	-12(1)	5(1)	12(1)
C(12B)	62(2)	50(2)	61(2)	-1(1)	13(1)	14(1)
C(13B)	65(2)	48(2)	63(2)	0(1)	19(1)	17(1)
C(14B)	68(2)	47(2)	64(2)	2(1)	21(1)	18(1)
C(15B)	67(2)	57(2)	64(2)	-2(1)	11(1)	22(1)
C(16B)	85(2)	59(2)	76(2)	-5(2)	23(2)	28(2)
C(17B)	102(2)	106(2)	109(3)	-4(2)	34(2)	59(2)

The anisotropic displacement factor exponent takes the form: $-2p^2[h^2 a^{*2}U_{11} + ... + 2 h k a^* b^* U_{12}]$

Figure X2. Compound **2b** with packing and short contacts.

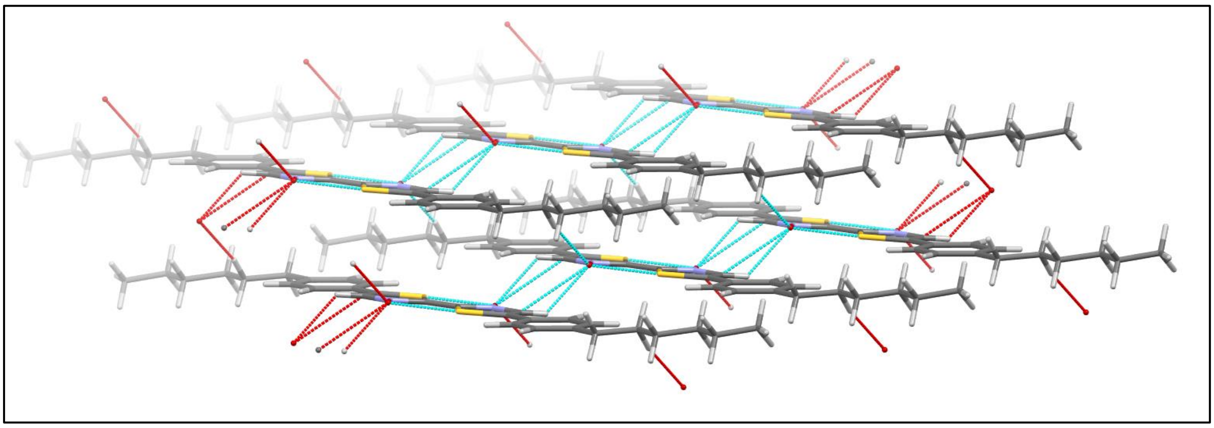

Table X5. Crystal data and structure refinement for (2b) $C_{30}H_{36}N_2OS_2$

Empirical formula	$C_{30}H_{36}N_2OS_2$
Formula weight	504.73
Temperature	296(2) K
Wavelength	0.71073 Å
Crystal system	Triclinic
Space group	*P*-1
Unit cell dimensions	*a* = 6.7333(17) Å, *b* = 7.2344(18) Å, *c* = 14.884(4) Å
	a= 85.396(7)°, *b*= 80.658(7)°, *g* = 74.043(7)°.
Volume	687.3(3) Å^3
Z	1
Density (calculated)	1.219 g/cm^3
Absorption coefficient	0.219 mm^{-1}
F(000)	270
Crystal size	0.310 x 0.250 x 0.010 mm^3
Theta range for data collection	2.776 to 28.000°.
Index ranges	-8<=h<=8, -9<=k<=9, -19<=l<=19
Reflections collected	12464
Independent reflections	3318 [R(int) = 0.0376]
Completeness to theta = 25.242°	100.0 %
Absorption correction	Semi-empirical from equivalents
Max. and min. transmission	0.7460 and 0.6539
Refinement method	Full-matrix least-squares on F^2
Data / restraints / parameters	3318 / 0 / 164
Goodness-of-fit on F^2	1.203
Final R indices [I>2sigma(I)]	R1 = 0.0490, wR2 = 0.0892
R indices (all data)	R1 = 0.0902, wR2 = 0.0998
Extinction coefficient	n/a

Largest diff. peak and hole 0.404 and -0.216 e.Å^{-3}

Table X6. Atomic coordinates (x 10^4) and equivalent isotropic displacement parameters (Å^{2}x 10^3) for $C_{30}H_{36}N_2OS_2$

	x	y	z	U(eq)
S(1)	2344(1)	-442(1)	5926(1)	56(1)
C(2)	3110(3)	1661(3)	5837(1)	50(1)
C(3)	2060(3)	2877(3)	5242(1)	61(1)
N(4)	693(3)	2250(3)	4851(1)	62(1)
C(5)	658(3)	487(3)	5153(1)	52(1)
O(6)	-456(5)	3022(4)	4260(2)	70(1)
C(7)	4670(3)	1935(3)	6368(1)	48(1)
C(8)	5714(3)	486(3)	6910(2)	64(1)
C(9)	7152(3)	780(3)	7408(2)	68(1)
C(10)	7626(3)	2514(3)	7383(1)	52(1)
C(11)	6593(4)	3956(3)	6833(1)	68(1)
C(12)	5131(4)	3682(3)	6335(1)	68(1)
C(13)	9225(3)	2740(3)	7944(1)	63(1)
C(14)	9564(3)	4699(3)	7970(1)	64(1)
C(15)	11218(3)	4695(3)	8559(1)	65(1)
C(16)	11534(4)	6638(3)	8666(2)	72(1)
C(17)	13201(4)	6573(4)	9239(2)	82(1)
C(18)	13506(5)	8506(4)	9392(2)	114(1)
H(3)	2260	4095	5102	73
H(8)	5448	-714	6941	77
H(9)	7822	-228	7773	82
H(11)	6881	5148	6794	81
H(12)	4454	4692	5974	81
H(13A)	8826	2318	8565	75
H(13B)	10555	1865	7717	75
H(14A)	8258	5594	8208	77
H(14B)	9992	5140	7355	77
H(15A)	10831	4157	9159	78
H(15B)	12536	3855	8294	78
H(16A)	10227	7477	8942	86
H(16B)	11907	7189	8067	86

H(17A)	14514	5768	8948	99
H(17B)	12852	5967	9827	99
H(18A)	14601	8335	9756	172
H(18B)	12233	9300	9703	172
H(18C)	13877	9112	8816	172

U(eq) is defined as one third of the trace of the orthogonalized U_{ij} tensor.

Table X7. Bond lengths [Å] and angles [°] for $C_{30}H_{36}N_2OS_2$

S(1)-C(5)	1.7157(19)
S(1)-C(2)	1.725(2)
C(2)-C(3)	1.340(3)
C(2)-C(7)	1.476(2)
C(3)-N(4)	1.355(2)
C(3)-H(3)	0.9300
N(4)-O(6)	1.261(3)
N(4)-C(5)	1.323(2)
C(5)-C(5)#1	1.419(4)
C(7)-C(8)	1.373(3)
C(7)-C(12)	1.377(3)
C(8)-C(9)	1.380(3)
C(8)-H(8)	0.9300
C(9)-C(10)	1.373(3)
C(9)-H(9)	0.9300
C(10)-C(11)	1.374(3)
C(10)-C(13)	1.515(3)
C(11)-C(12)	1.387(3)
C(11)-H(11)	0.9300
C(12)-H(12)	0.9300
C(13)-C(14)	1.501(3)
C(13)-H(13A)	0.9700
C(13)-H(13B)	0.9700
C(14)-C(15)	1.525(3)
C(14)-H(14A)	0.9700
C(14)-H(14B)	0.9700
C(15)-C(16)	1.502(3)
C(15)-H(15A)	0.9700

C(15)-H(15B)	0.9700
C(16)-C(17)	1.505(3)
C(16)-H(16A)	0.9700
C(16)-H(16B)	0.9700
C(17)-C(18)	1.508(3)
C(17)-H(17A)	0.9700
C(17)-H(17B)	0.9700
C(18)-H(18A)	0.9600
C(18)-H(18B)	0.9600
C(18)-H(18C)	0.9600
C(5)-S(1)-C(2)	90.20(10)
C(3)-C(2)-C(7)	129.35(19)
C(3)-C(2)-S(1)	108.75(15)
C(7)-C(2)-S(1)	121.90(15)
C(2)-C(3)-N(4)	116.9(2)
C(2)-C(3)-H(3)	121.5
N(4)-C(3)-H(3)	121.5
O(6)-N(4)-C(5)	117.3(2)
O(6)-N(4)-C(3)	131.7(2)
C(5)-N(4)-C(3)	111.03(17)
N(4)-C(5)-C(5)#1	121.8(2)
N(4)-C(5)-S(1)	113.07(14)
C(5)#1-C(5)-S(1)	125.2(2)
C(8)-C(7)-C(12)	117.40(18)
C(8)-C(7)-C(2)	122.12(18)
C(12)-C(7)-C(2)	120.48(18)
C(7)-C(8)-C(9)	121.2(2)
C(7)-C(8)-H(8)	119.4
C(9)-C(8)-H(8)	119.4
C(10)-C(9)-C(8)	122.1(2)
C(10)-C(9)-H(9)	119.0
C(8)-C(9)-H(9)	119.0
C(9)-C(10)-C(11)	116.65(18)
C(9)-C(10)-C(13)	119.44(18)
C(11)-C(10)-C(13)	123.90(19)
C(10)-C(11)-C(12)	121.8(2)
C(10)-C(11)-H(11)	119.1

C(12)-C(11)-H(11)	119.1
C(7)-C(12)-C(11)	120.9(2)
C(7)-C(12)-H(12)	119.5
C(11)-C(12)-H(12)	119.5
C(14)-C(13)-C(10)	118.16(18)
C(14)-C(13)-H(13A)	107.8
C(10)-C(13)-H(13A)	107.8
C(14)-C(13)-H(13B)	107.8
C(10)-C(13)-H(13B)	107.8
H(13A)-C(13)-H(13B)	107.1
C(13)-C(14)-C(15)	112.00(18)
C(13)-C(14)-H(14A)	109.2
C(15)-C(14)-H(14A)	109.2
C(13)-C(14)-H(14B)	109.2
C(15)-C(14)-H(14B)	109.2
H(14A)-C(14)-H(14B)	107.9
C(16)-C(15)-C(14)	114.79(18)
C(16)-C(15)-H(15A)	108.6
C(14)-C(15)-H(15A)	108.6
C(16)-C(15)-H(15B)	108.6
C(14)-C(15)-H(15B)	108.6
H(15A)-C(15)-H(15B)	107.5
C(15)-C(16)-C(17)	113.27(19)
C(15)-C(16)-H(16A)	108.9
C(17)-C(16)-H(16A)	108.9
C(15)-C(16)-H(16B)	108.9
C(17)-C(16)-H(16B)	108.9
H(16A)-C(16)-H(16B)	107.7
C(16)-C(17)-C(18)	114.8(2)
C(16)-C(17)-H(17A)	108.6
C(18)-C(17)-H(17A)	108.6
C(16)-C(17)-H(17B)	108.6
C(18)-C(17)-H(17B)	108.6
H(17A)-C(17)-H(17B)	107.5
C(17)-C(18)-H(18A)	109.5
C(17)-C(18)-H(18B)	109.5
H(18A)-C(18)-H(18B)	109.5
C(17)-C(18)-H(18C)	109.5

H(18A)-C(18)-H(18C)	109.5
H(18B)-C(18)-H(18C)	109.5

Symmetry transformations used to generate equivalent atoms:

#1 -x,-y,-z+1

Table X8. Anisotropic displacement parameters (Å^{2}x 10^3) for $C_{30}H_{36}N_2OS_2$

	U_{11}	U_{22}	U_{33}	U_{23}	U_{13}	U_{12}
S(1)	52(1)	57(1)	60(1)	-2(1)	-18(1)	-12(1)
C(2)	44(1)	55(1)	52(1)	-10(1)	-6(1)	-11(1)
C(3)	54(1)	65(1)	68(1)	-2(1)	-24(1)	-15(1)
N(4)	57(1)	64(1)	68(1)	-3(1)	-22(1)	-14(1)
C(5)	43(1)	60(1)	49(1)	-8(1)	-9(1)	-5(1)
O(6)	90(2)	46(2)	88(2)	14(1)	-53(2)	-22(2)
C(7)	40(1)	55(1)	48(1)	-7(1)	-6(1)	-10(1)
C(8)	59(1)	57(1)	85(2)	7(1)	-28(1)	-21(1)
C(9)	60(1)	65(2)	83(2)	12(1)	-32(1)	-14(1)
C(10)	44(1)	63(1)	49(1)	-6(1)	-7(1)	-14(1)
C(11)	88(2)	58(1)	71(1)	0(1)	-31(1)	-31(1)
C(12)	83(2)	58(1)	69(1)	5(1)	-36(1)	-18(1)
C(13)	55(1)	76(2)	60(1)	-7(1)	-16(1)	-18(1)
C(14)	54(1)	77(2)	68(1)	-5(1)	-17(1)	-22(1)
C(15)	53(1)	82(2)	65(1)	-8(1)	-16(1)	-21(1)
C(16)	68(2)	83(2)	74(2)	0(1)	-24(1)	-29(1)
C(17)	69(2)	104(2)	87(2)	-10(1)	-26(1)	-35(1)
C(18)	134(3)	125(3)	117(2)	-1(2)	-42(2)	-78(2)

The anisotropic displacement factor exponent takes the form: $-2p^2[h^2 a^{*2} U_{11} + ... + 2 h k a^* b^* U_{12}]$

8. References

1. Truong, T.; Alvarado, J.; Tran, L. D.; Daugulis, O. *Org. Lett.* **2010**, 12, 1200 – 1203.
2. Liang, Z.; Zhao, J.; Zhang, Y. *J. Org. Chem.* **2010**, 75, 170 – 177.
3. Mirabal, R. A.; Vanderzwet, L.; Abuadas, S.; Emmett, M.; Schipper, D. J. *Chem. Eur. J.* **2018**, *24*, 12231-12235.

9. Computational Data

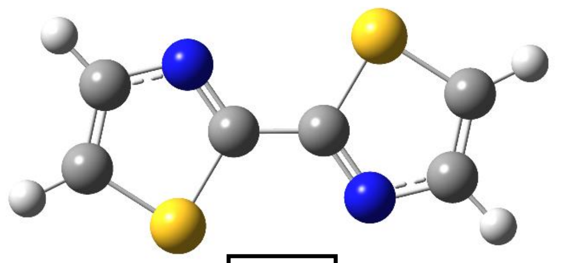

1a

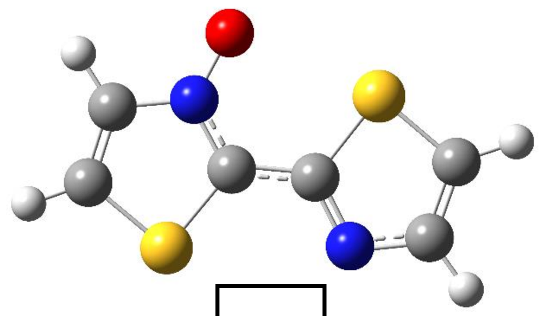

1b

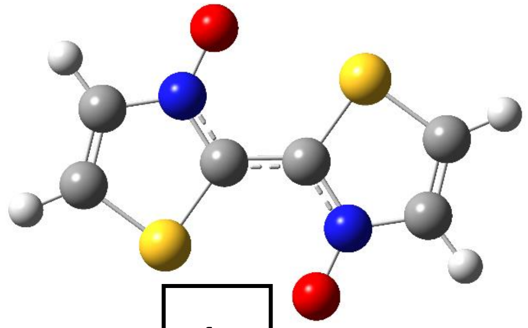

1c

Table S2 – Cartesian coordinates for compounds **1a-c** at **N-C-C-N** 90 – 180° (10° increments) calculated at the DFT B3LYP 6-311G++(d,p) level of theory.

	**1a** 90°			**1a** 100°			**1a** 110°			**1a** 120°			**1a** 130°		
**C**	-0.02428	0.72508	0	-0.02428	0.72508	0	-0.02428	0.72508	0	-0.02428	0.72508	0	-0.02428	0.72508	0
**C**	0.44741	2.81065	-0.54114	0.49249	2.81216	-0.49191	0.53311	2.81352	-0.43893	0.56896	2.81472	-0.38262	0.59977	2.81575	-0.3234
**C**	-0.50496	3.07316	0.40199	-0.53845	3.07204	0.36543	-0.56862	3.07103	0.3261	-0.59526	3.07014	0.28428	-0.61815	3.06938	0.2403
**S**	-1.11725	1.59647	1.06305	-1.2058	1.5935	0.96634	-1.2856	1.59083	0.86227	-1.35602	1.58847	0.75165	-1.41654	1.58645	0.6353
**H**	0.98605	3.56038	-1.1045	1.07806	3.56346	-1.00402	1.16096	3.56624	-0.8959	1.23414	3.56869	-0.78096	1.29702	3.57079	-0.66008
**H**	-0.87259	4.03034	0.73747	-0.93403	4.02828	0.67041	-0.98938	4.02643	0.59825	-1.03825	4.02479	0.52153	-1.08024	4.02338	0.44084
**N**	0.71297	1.48691	-0.76232	0.77647	1.48903	-0.69298	0.83369	1.49095	-0.61836	0.8842	1.49264	-0.53904	0.9276	1.49409	-0.45561
**C**	0.02428	-0.72508	0	0.02428	-0.72508	0	0.02428	-0.72508	0	0.02428	-0.72508	0	0.02428	-0.72508	0
**S**	1.11725	-1.59647	1.06305	1.2058	-1.5935	0.96634	1.2856	-1.59083	0.86227	1.35602	-1.58847	0.75165	1.41654	-1.58645	0.6353
**C**	-0.44741	-2.81065	-0.54114	-0.49249	-2.81216	-0.49191	-0.53311	-2.81352	-0.43893	-0.56896	-2.81472	-0.38262	-0.59977	-2.81575	-0.3234
**C**	0.50496	-3.07316	0.40199	0.53845	-3.07204	0.36543	0.56862	-3.07103	0.3261	0.59526	-3.07014	0.28428	0.61815	-3.06938	0.2403
**H**	-0.98605	-3.56038	-1.1045	-1.07806	-3.56346	-1.00402	-1.16096	-3.56624	-0.8959	-1.23414	-3.56869	-0.78096	-1.29702	-3.57079	-0.66008
**H**	0.87259	-4.03034	0.73747	0.93403	-4.02828	0.67041	0.98938	-4.02643	0.59825	1.03825	-4.02479	0.52153	1.08024	-4.02338	0.44084
**N**	-0.71297	-1.48691	-0.76232	-0.77647	-1.48903	-0.69298	-0.83369	-1.49095	-0.61836	-0.8842	-1.49264	-0.53904	-0.9276	-1.49409	-0.45561
	**1a** 140°			**1a** 150°			**1a** 160°			**1a** 170°			**1a** 180°		
**C**	-0.02428	0.72508	0	-0.02428	0.72508	0	-0.02428	0.72508	0	-0.02428	0.72508	0.	-0.02428	0.72508	0.

C	0.6253	2.8166	-0.26171	0.64536	2.81727	-0.19803	0.6598	2.81776	-0.13285	0.66851	2.81805	-0.06665	0.67141	2.81815	0.00002
C	-0.63712	3.06874	0.19449	-0.65203	3.06824	0.14721	-0.66277	3.06788	0.0988	-0.66924	3.06766	0.04964	-0.67141	3.06759	0.00012
S	-1.4667	1.58477	0.51412	-1.50611	1.58345	0.38902	-1.53447	1.5825	0.26097	-1.55157	1.58193	0.13093	-1.55728	1.58174	-0.00005
H	1.34913	3.57254	-0.53418	1.39008	3.57391	-0.40421	1.41955	3.5749	-0.27116	1.43732	3.57549	-0.13605	1.44325	3.57569	0.00003
H	-1.11505	4.02222	0.3568	-1.1424	4.0213	0.27005	-1.16209	4.02064	0.18123	-1.17397	4.02025	0.09104	-1.17795	4.02011	0.0002
N	0.96357	1.4953	-0.36872	0.99184	1.49624	-0.27902	1.01219	1.49692	-0.1872	1.02445	1.49734	-0.09396	1.02856	1.49747	-0.00004
C	0.02428	-0.72508	0	0.02428	-0.72508	0	0.02428	-0.72508	0	0.02428	-0.72508	0.	0.02428	-0.72508	0.
S	1.4667	-1.58477	0.51412	1.50611	-1.58345	0.38902	1.53447	-1.5825	0.26097	1.55157	-1.58193	0.13093	1.55728	-1.58174	-0.00005
C	-0.6253	-2.8166	-0.26171	-0.64536	-2.81727	-0.19803	-0.6598	-2.81776	-0.13285	-0.66851	-2.81805	-0.06665	-0.67141	-2.81815	0.00002
C	0.63712	-3.06874	0.19449	0.65203	-3.06824	0.14721	0.66277	-3.06788	0.0988	0.66924	-3.06766	0.04964	0.67141	-3.06759	0.00012
H	-1.34913	-3.57254	-0.53418	-1.39008	-3.57391	-0.40421	-1.41955	-3.5749	-0.27116	-1.43732	-3.57549	-0.13605	-1.44325	-3.57569	0.00003
H	1.11505	-4.02222	0.3568	1.1424	-4.0213	0.27005	1.16209	-4.02064	0.18123	1.17397	-4.02025	0.09104	1.17795	-4.02011	0.0002
N	-0.96357	-1.4953	-0.36872	-0.99184	-1.49624	-0.27902	-1.01219	-1.49692	-0.1872	-1.02445	-1.49734	-0.09396	-1.02856	-1.49747	-0.00004
	**1b** 90°			**1b** 100°			**1b** 110°			**1b** 120°			**1b** 130°		
C	-0.78955	-0.2849	-0.00002	-0.78955	-0.2849	-0.00002	-0.78955	-0.2849	-0.00002	-0.78955	-0.2849	-0.00002	-0.78955	-0.2849	-0.00002
C	-2.76318	-1.06855	0.59258	-2.75848	-1.11773	0.53868	-2.75425	-1.16204	0.48068	-2.75052	-1.20114	0.41902	-2.74731	-1.23475	0.35417
C	-3.18487	-0.16647	-0.34558	-3.18761	-0.1378	-0.31415	-3.19007	-0.11197	-0.28034	-3.19225	-0.08917	-0.24438	-3.19412	-0.06958	-0.20657
S	-1.83846	0.66413	-1.0445	-1.84673	0.75079	-0.9495	-1.85419	0.82888	-0.84727	-1.86077	0.8978	-0.7386	-1.86643	0.95704	-0.6243
H	-3.41033	-1.7128	1.17246	-3.40104	-1.81009	1.06581	-3.39267	-1.89775	0.95106	-3.38528	-1.97513	0.82906	-3.37893	-2.04162	0.70076
H	-4.19533	0.04963	-0.65678	-4.20054	0.10412	-0.59705	-4.20522	0.15322	-0.53277	-4.20936	0.19655	-0.46445	-4.21292	0.23379	-0.39259
N	-1.41502	-1.13312	0.78502	-1.4088	-1.19826	0.71362	-1.40319	-1.25696	0.63679	-1.39825	-1.30876	0.55511	-1.394	-1.35329	0.46921
C	0.63436	-0.14898	0.00001	0.63436	-0.14898	0.00001	0.63436	-0.14898	0.00001	0.63436	-0.14898	0.00001	0.63436	-0.14898	0.00001
S	1.70339	-1.06722	-1.01547	1.71143	-1.15148	-0.92309	1.71868	-1.2274	-0.82368	1.72507	-1.29441	-0.718	1.73057	-1.352	-0.60685
C	2.68874	0.66005	0.61012	2.68391	0.71067	0.55462	2.67956	0.75628	0.4949	2.67571	0.79653	0.43141	2.67241	0.83113	0.36465
C	3.06478	-0.24051	-0.32199	3.06733	-0.26723	-0.29269	3.06963	-0.2913	-0.26117	3.07165	-0.31256	-0.22766	3.0734	-0.33082	-0.19241
H	3.29111	1.32159	1.21137	3.28152	1.4221	1.10117	3.27288	1.51266	0.9826	3.26525	1.59259	0.85654	3.2587	1.66128	0.72397
H	4.06767	-0.46902	-0.64472	4.07277	-0.52252	-0.58606	4.07737	-0.57073	-0.52295	4.08143	-0.61327	-0.45585	4.08492	-0.64984	-0.38528
N	1.3019	0.7118	0.79338	1.29562	0.77763	0.7212	1.28996	0.83694	0.64354	1.28496	0.88929	0.56098	1.28067	0.93428	0.47416
O	0.73048	1.4986	1.63083	0.71757	1.63391	1.48248	0.70593	1.75584	1.32284	0.69566	1.86345	1.15313	0.68684	1.95593	0.97465
	**1b** 140°			**1b** 150°			**1b** 160°			**1b** 170°			**1b** 180°		

**C**	-0.78955	-0.2849	-0.00002	-0.78955	-0.2849	-0.00002	-0.78955	-0.2849	-0.00002	-0.78955	-0.2849	-0.00002	-0.78955	-0.2849	-0.00002
**C**	-2.74465	-1.26261	0.28663	-2.74256	-1.2845	0.2169	-2.74105	-1.30026	0.14552	-2.74014	-1.30976	0.07304	-2.73984	-1.31294	0.00001
**C**	-3.19567	-0.05334	-0.16719	-3.19689	-0.04057	-0.12654	-3.19777	-0.03139	-0.08492	-3.1983	-0.02585	-0.04266	-3.19848	-0.024	-0.00008
**S**	-1.87111	1.00613	-0.50526	-1.8748	1.04471	-0.38237	-1.87745	1.07247	-0.25657	-1.87905	1.08922	-0.12881	-1.87959	1.09482	-0.00011
**H**	-3.37367	-2.09674	0.56712	-3.36953	-2.14004	0.42917	-3.36655	-2.17122	0.28795	-3.36476	-2.19002	0.14453	-3.36415	-2.19631	0.00005
**H**	-4.21587	0.26466	-0.31774	-4.21818	0.28892	-0.24047	-4.21985	0.30637	-0.16138	-4.22086	0.3169	-0.08106	-4.22119	0.32043	-0.00013
**N**	-1.39047	-1.39019	0.37974	-1.3877	-1.41918	0.28737	-1.38571	-1.44006	0.19282	-1.3845	-1.45265	0.0968	-1.3841	-1.45686	0.00006
**C**	0.63436	-0.14898	0.00001	0.63436	-0.14898	0.00001	0.63436	-0.14898	0.00001	0.63436	-0.14898	0.00001	0.63436	-0.14898	0.00001
**S**	1.73512	-1.39972	-0.49109	1.7387	-1.43722	-0.37159	1.74127	-1.4642	-0.24926	1.74282	-1.48048	-0.12503	1.74334	-1.48591	0.00012
**C**	2.66968	0.8598	0.2951	2.66753	0.88233	0.22331	2.66598	0.89854	0.14982	2.66505	0.90832	0.0752	2.66474	0.91158	0.00001
**C**	3.07484	-0.34595	-0.1557	3.07598	-0.35785	-0.11781	3.07679	-0.36641	-0.07902	3.07728	-0.37157	-0.03963	3.07745	-0.37329	0.00006
**H**	3.25326	1.7182	0.58589	3.249	1.76293	0.44335	3.24593	1.79512	0.29744	3.24408	1.81453	0.14927	3.24346	1.82102	-0.00002
**H**	4.08782	-0.68014	-0.31179	4.09009	-0.70395	-0.23591	4.09172	-0.72109	-0.15824	4.09271	-0.73143	-0.07937	4.09303	-0.73488	0.00009
**N**	1.27711	0.97157	0.38372	1.27432	1.00086	0.29036	1.27231	1.02195	0.1948	1.27109	1.03466	0.09775	1.27069	1.0389	-0.00003
**O**	0.67952	2.03257	0.78875	0.67378	2.0928	0.59685	0.66965	2.13614	0.40041	0.66716	2.16227	0.20091	0.66633	2.171	-0.00007
	**1c** 90°			**1c** 100°			**1c** 110°			**1c** 120°			**1c** 130°		
**C**	-0.05492	0.7044	0	-0.05492	0.7044	0	-0.05492	0.7044	0	-0.05492	0.7044	0	-0.05492	0.7044	0
**C**	-0.89118	2.72309	-0.67681	-0.94742	2.71871	-0.61525	-0.9981	2.71475	-0.549	-1.04283	2.71127	-0.47858	-1.08127	2.70827	-0.40451
**C**	0.00561	3.13393	0.2492	0.02632	3.13555	0.22653	0.04497	3.137	0.20214	0.06144	3.13829	0.17621	0.0756	3.13939	0.14894
**S**	0.85309	1.80978	0.99119	0.93546	1.8162	0.90103	1.00967	1.82198	0.80401	1.07518	1.82709	0.70087	1.13148	1.83148	0.59241
**H**	-1.55817	3.29987	-1.29695	-1.66595	3.29147	-1.17898	-1.76306	3.2839	-1.05203	-1.84877	3.27722	-0.91708	-1.92243	3.27147	-0.77515
**H**	0.21811	4.15037	0.54007	0.26299	4.15387	0.49094	0.30343	4.15702	0.43808	0.33912	4.1598	0.38189	0.3698	4.1622	0.32278
**N**	0.05492	-0.7044	0	0.05492	-0.7044	0	0.05492	-0.7044	0	0.05492	-0.7044	0	0.05492	-0.7044	0
**C**	-0.85309	-1.80978	0.99119	-0.93546	-1.8162	0.90103	-1.00967	-1.82198	0.80401	-1.07518	-1.82709	0.70087	-1.13148	-1.83148	0.59241
**S**	0.89118	-2.72309	-0.67681	0.94742	-2.71871	-0.61525	0.9981	-2.71475	-0.549	1.04283	-2.71127	-0.47858	1.08127	-2.70827	-0.40451
**C**	-0.00561	-3.13393	0.2492	-0.02632	-3.13555	0.22653	-0.04497	-3.137	0.20214	-0.06144	-3.13829	0.17621	-0.0756	-3.13939	0.14894
**C**	1.55817	-3.29987	-1.29695	1.66595	-3.29147	-1.17898	1.76306	-3.2839	-1.05203	1.84877	-3.27722	-0.91708	1.92243	-3.27147	-0.77515
**H**	-0.21811	-4.15037	0.54007	-0.26299	-4.15387	0.49094	-0.30343	-4.15702	0.43808	-0.33912	-4.1598	0.38189	-0.3698	-4.1622	0.32278
**H**	-0.92468	1.33879	-0.81782	-0.99264	1.3335	-0.74343	-1.05388	1.32872	-0.66338	-1.10793	1.32451	-0.57829	-1.15438	1.32089	-0.48879
**N**	0.92468	-1.33879	-0.81782	0.99264	-1.3335	-0.74343	1.05388	-1.32872	-0.66338	1.10793	-1.32451	-0.57829	1.15438	-1.32089	-0.48879

**O**	-1.69835	0.7147	-1.63765	-1.83443	0.70409	-1.48869	-1.95706	0.69453	-1.3284	-2.06529	0.68609	-1.158	-2.1583	0.67884	-0.97878
**O**	1.69835	-0.7147	-1.63765	1.83443	-0.70409	-1.48869	1.95706	-0.69453	-1.3284	2.06529	-0.68609	-1.158	2.1583	-0.67884	-0.97878
	**1c** 140°			**1c** 150°			**1c** 160°			**1c** 170°			**1c** 180°		
**C**	-0.05492	0.7044	0	-0.05492	0.7044	0	-0.05492	0.7044	0	-0.05492	0.7044	0	-0.05492	0.7044	0
**C**	-1.11313	2.70579	-0.32737	-1.13816	2.70383	-0.24773	-1.15618	2.70243	-0.16621	-1.16704	2.70158	-0.08342	-1.17067	2.7013	0
**C**	0.08733	3.14031	0.12053	0.09654	3.14102	0.09121	0.10318	3.14154	0.0612	0.10718	3.14185	0.03072	0.10852	3.14196	0
**S**	1.17813	1.83512	0.47943	1.21479	1.83798	0.3628	1.24118	1.84003	0.24341	1.25709	1.84128	0.12217	1.26241	1.84169	0
**H**	-1.98348	3.26671	-0.62732	-2.03145	3.26297	-0.47472	-2.06598	3.26028	-0.3185	-2.0868	3.25866	-0.15986	-2.09376	3.25812	0
**H**	0.39522	4.16418	0.26123	0.41519	4.16574	0.19768	0.42957	4.16686	0.13263	0.43824	4.16753	0.06657	0.44114	4.16776	0
**N**	0.05492	-0.7044	0	0.05492	-0.7044	0	0.05492	-0.7044	0	0.05492	-0.7044	0	0.05492	-0.7044	0
**C**	-1.17813	-1.83512	0.47943	-1.21479	-1.83798	0.3628	-1.24118	-1.84003	0.24341	-1.25709	-1.84128	0.12217	-1.26241	-1.84169	0
**S**	1.11313	-2.70579	-0.32737	1.13816	-2.70383	-0.24773	1.15618	-2.70243	-0.16621	1.16704	-2.70158	-0.08342	1.17067	-2.7013	0
**C**	-0.08733	-3.14031	0.12053	-0.09654	-3.14102	0.09121	-0.10318	-3.14154	0.0612	-0.10718	-3.14185	0.03072	-0.10852	-3.14196	0
**C**	1.98348	-3.26671	-0.62732	2.03145	-3.26297	-0.47472	2.06598	-3.26028	-0.3185	2.0868	-3.25866	-0.15986	2.09376	-3.25812	0
**H**	-0.39522	-4.16418	0.26123	-0.41519	-4.16574	0.19768	-0.42957	-4.16686	0.13263	-0.43824	-4.16753	0.06657	-0.44114	-4.16776	0
**H**	-1.19287	1.31788	-0.39557	-1.22312	1.31553	-0.29934	-1.24489	1.31383	-0.20084	-1.25802	1.3128	-0.1008	-1.26241	1.31246	0
**N**	1.19287	-1.31788	-0.39557	1.22312	-1.31553	-0.29934	1.24489	-1.31383	-0.20084	1.25802	-1.3128	-0.1008	1.26241	-1.31246	0
**O**	-2.23539	0.67282	-0.79212	-2.29596	0.6681	-0.59942	-2.33956	0.6647	-0.40217	-2.36585	0.66265	-0.20185	-2.37464	0.66197	0
**O**	2.23539	-0.67282	-0.79212	2.29596	-0.6681	-0.59942	2.33956	-0.6647	-0.40217	2.36585	-0.66265	-0.20185	2.37464	-0.66197	0

Table S3 – Total Molecular Energies (Hartrees) calculated with ccsd(t) 6-311++g(d,p) on the DFT optimized structures.

Angle (°)	**1a**	**1b**	**1c**
90	-1133.540487	-1208.328794	-1283.118228
100	-1133.541316	-1208.329786	-1283.119401
110	-1133.542695	-1208.331077	-1283.120752
120	-1133.544504	-1208.33269	-1283.122429
130	-1133.546524	-1208.334666	-1283.124697
140	-1133.548498	-1208.337054	-1283.127874
150	-1133.550204	-1208.339791	-1283.131978
160	-1133.55149	-1208.342535	-1283.136397

170	-1133.552278	-1208.344641	-1283.139914
180	-1133.552542	-1208.345438	-1283.141265

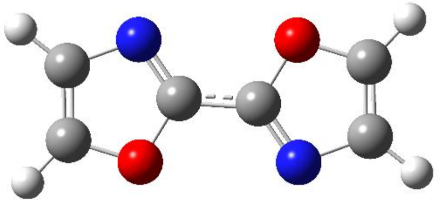

O1

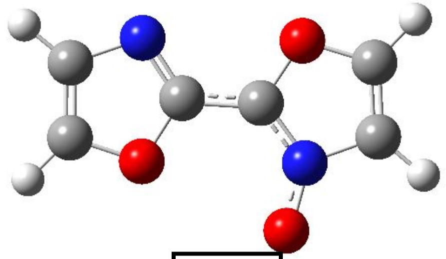

O2

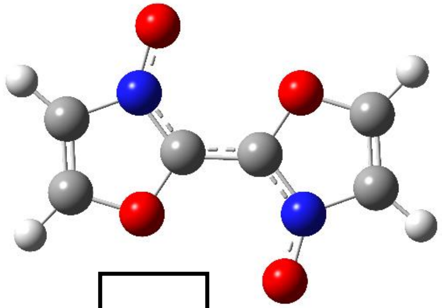

O3

Table S4 – Cartesian coordinates for 2,2'-bioxazoles **O1-3** at **N-C-C-N** 90 – 180° (10° increments) calculated at the DFT B3LYP 6-311G++(d,p) level of theory.

	**O1** 90°			**O1** 100°			**O1** 110°			**O1** 120°			**O1** 130°		
**C**	-0.09424	0.71694	-0.00002	-0.09424	0.71694	-0.00002	-0.09424	0.71694	-0.00002	-0.09424	0.71694	-0.00002	-0.09424	0.71694	-0.00002
**C**	-0.03995	2.81176	-0.32681	-0.01294	2.81531	-0.29708	0.01139	2.81851	-0.26509	0.03287	2.82133	-0.23108	0.05132	2.82375	-0.19531
**C**	-0.97325	2.57742	0.62914	-1.02524	2.57059	0.57192	-1.07210	2.56443	0.51035	-1.11345	2.55899	0.44490	-1.14899	2.55432	0.37605
**H**	0.26894	3.75126	-0.75546	0.33137	3.75947	-0.68674	0.38763	3.76686	-0.61279	0.43728	3.77339	-0.53417	0.47994	3.77900	-0.45149
**H**	-1.64126	3.18713	1.21208	-1.74143	3.17396	1.10185	-1.83169	3.16209	0.98323	-1.91136	3.15162	0.85712	-1.97983	3.14262	0.72449
**N**	0.51678	1.60809	-0.72197	0.57644	1.61594	-0.65630	0.63021	1.62300	-0.58564	0.67765	1.62924	-0.51052	0.71843	1.63460	-0.43151
**C**	0.09424	-0.71694	-0.00002	0.09424	-0.71694	-0.00002	0.09424	-0.71694	-0.00002	0.09424	-0.71694	-0.00002	0.09424	-0.71694	-0.00002
**C**	0.03995	-2.81176	-0.32681	0.01294	-2.81531	-0.29708	-0.01139	-2.81851	-0.26509	-0.03287	-2.82133	-0.23108	-0.05132	-2.82375	-0.19531
**C**	0.97325	-2.57742	0.62914	1.02524	-2.57059	0.57192	1.07210	-2.56443	0.51035	1.11345	-2.55899	0.44490	1.14899	-2.55432	0.37605
**H**	-0.26894	-3.75126	-0.75546	-0.33137	-3.75947	-0.68674	-0.38763	-3.76686	-0.61279	-0.43728	-3.77339	-0.53417	-0.47994	-3.77900	-0.45149
**H**	1.64126	-3.18713	1.21208	1.74143	-3.17396	1.10185	1.83169	-3.16209	0.98323	1.91136	-3.15162	0.85712	1.97983	-3.14262	0.72449
**N**	-0.51678	-1.60809	-0.72197	-0.57644	-1.61594	-0.65630	-0.63021	-1.62300	-0.58564	-0.67765	-1.62924	-0.51052	-0.71843	-1.63460	-0.43151
**O**	-1.01973	1.23043	0.85057	-1.09003	1.22119	0.77319	-1.15336	1.21286	0.68993	-1.20927	1.20551	0.60142	-1.25731	1.19920	0.50833
**O**	1.01973	-1.23043	0.85057	1.09003	-1.22119	0.77319	1.15336	-1.21286	0.68993	1.20927	-1.20551	0.60142	1.25731	-1.19920	0.50833

	**O1** 140°			**O1** 150°			**O1** 160°			**O1** 170°			**O1** 180°		
**C**	-0.09424	0.716944	-0.00001	-0.09424	0.716944	-0.00001	-0.09424	0.716944	-0.00001	-0.09424	0.716944	-0.00001	-0.09424	0.716944	-1.7E-05
**C**	0.066618	2.825764	-0.15806	0.078636	2.827344	-0.11960	0.087286	2.828481	-0.08023	0.092501	2.829167	-0.04026	0.094242	2.829396	0.000007
**C**	-1.17844	2.550449	0.304350	-1.20158	2.547406	0.230330	-1.21825	2.545216	0.154557	-1.22830	2.543895	0.077607	-1.23167	2.543453	0.000091
**H**	0.515301	3.783646	-0.36537	0.543085	3.787298	-0.27647	0.563081	3.789927	-0.18547	0.575137	3.791511	-0.09306	0.579162	3.792041	0.000027
**H**	-2.03657	3.135162	0.586351	-2.08116	3.129300	0.443746	-2.11326	3.125081	0.297765	-2.13263	3.122535	0.149516	-2.13911	3.121684	0.000176
**N**	0.752227	1.639042	-0.34921	0.778783	1.642532	-0.26427	0.797897	1.645045	-0.17731	0.809423	1.646560	-0.08900	0.813276	1.647067	-4.4E-05
**C**	0.094242	-0.71694	-0.00001	0.094242	-0.71694	-0.00001	0.094242	-0.71694	-0.00001	0.094242	-0.71694	-0.00001	0.094242	-0.71694	-1.7E-05
**C**	-0.06661	-2.82576	-0.15806	-0.07863	-2.82734	-0.11960	-0.08728	-2.82848	-0.08023	-0.09250	-2.82916	-0.04026	-0.09424	-2.8294	0.000007
**C**	1.178442	-2.55044	0.304350	1.201589	-2.54740	0.230330	1.218252	-2.54521	0.154557	1.228304	-2.54389	0.077607	1.231668	-2.54345	0.000091
**H**	-0.51530	-3.78364	-0.36537	-0.54308	-3.78729	-0.27647	-0.56308	-3.78992	-0.18547	-0.57513	-3.79151	-0.09306	-0.57916	-3.79204	0.000027
**H**	2.036573	-3.13516	0.586351	2.081166	-3.12930	0.443746	2.113266	-3.12508	0.297765	2.132630	-3.12253	0.149516	2.13911	-3.12168	0.000176
**N**	-0.75222	-1.63904	-0.34921	-0.77878	-1.64253	-0.26427	-0.79789	-1.64504	-0.17731	-0.80942	-1.64656	-0.08900	-0.81328	-1.64707	-4.4E-05
**O**	-1.29711	1.193965	0.411365	-1.32840	1.189853	0.311275	-1.35091	1.186893	0.208815	-1.36449	1.185108	0.104766	-1.36903	1.184513	-4.8E-05
**O**	1.297119	-1.19396	0.411365	1.328403	-1.18985	0.311275	1.350918	-1.18689	0.208815	1.364494	-1.18510	0.104766	1.369028	-1.18451	-4.8E-05
	**O2** 90°			**O2** 100°			**O2** 110°			**O2** 120°			**O2** 130°		
**C**	-0.87312	-0.21381	0.00001	-0.87312	-0.21381	0.00001	-0.87312	-0.21381	0.00001	-0.87312	-0.21381	0.00001	-0.87312	-0.21381	0.00001
**C**	-2.93486	-0.54452	0.36683	-2.93539	-0.57510	0.33347	-2.93587	-0.60265	0.29756	-2.93630	-0.62696	0.25939	-2.93667	-0.64786	0.21925
**C**	-2.84283	0.41207	-0.59136	-2.84197	0.46135	-0.53758	-2.84119	0.50575	-0.47971	-2.84051	0.54495	-0.41819	-2.83992	0.57863	-0.35349
**H**	-3.81770	-0.97414	0.81190	-3.81889	-1.04181	0.73805	-3.81996	-1.10278	0.65858	-3.82090	-1.15659	0.57410	-3.82171	-1.20284	0.48526
**H**	-3.54509	0.99434	-1.16133	-3.54339	1.09112	-1.05572	-3.54186	1.17833	-0.94207	-3.54051	1.25530	-0.82125	-3.53936	1.32146	-0.69418
**N**	-1.66444	-0.94020	0.74025	-1.66552	-1.00189	0.67292	-1.66650	-1.05748	0.60047	-1.66736	-1.10654	0.52346	-1.66810	-1.14871	0.44246
**C**	0.55588	-0.23889	0.00003	0.55588	-0.23889	0.00003	0.55588	-0.23889	0.00003	0.55588	-0.23889	0.00003	0.55588	-0.23889	0.00003
**C**	2.71676	0.06276	0.33946	2.71726	0.09104	0.30858	2.71771	0.11653	0.27535	2.71810	0.13902	0.24003	2.71844	0.15835	0.20288
**C**	2.54907	-0.87973	-0.60576	2.54818	-0.93022	-0.55065	2.54739	-0.97571	-0.49136	2.54668	-1.01586	-0.42833	2.54607	-1.05037	-0.36203
**H**	3.58475	0.50351	0.79528	3.58591	0.56978	0.72293	3.58696	0.62950	0.64508	3.58789	0.68220	0.56232	3.58868	0.72748	0.47528
**H**	3.23077	-1.47422	-1.18820	3.22903	-1.57325	-1.08012	3.22747	-1.66249	-0.96382	3.22608	-1.74124	-0.84018	3.22489	-1.80893	-0.71015
**N**	1.42596	0.47833	0.73233	1.42703	0.53936	0.66571	1.42799	0.59435	0.59403	1.42885	0.64288	0.51782	1.42958	0.68459	0.43768
**O**	1.18309	1.36234	1.61182	1.18545	1.49666	1.46519	1.18758	1.61769	1.30741	1.18945	1.72452	1.13968	1.19107	1.81632	0.96328
**O**	-1.51731	0.63655	-0.83901	-1.51608	0.70647	-0.76270	-1.51498	0.76947	-0.68058	-1.51400	0.82508	-0.59329	-1.51317	0.87287	-0.50148

**O**	1.22163	-1.07838	-0.82748	1.22042	-1.14734	-0.75219	1.21933	-1.20948	-0.67118	1.21837	-1.26433	-0.58506	1.21754	-1.31146	-0.49449
	**O2** 140°			**O2** 150°			**O2** 160°			**O2** 170°			**O2** 180°		
**C**	-0.87312	-0.21381	0.00001	-0.87312	-0.21381	0.00001	-0.87312	-0.21381	0.00001	-0.87312	-0.21381	0.00001	-0.87312	-0.21381	0.00001
**C**	-2.93697	-0.66518	0.17743	-2.93721	-0.67879	0.13427	-2.93738	-0.68858	0.09008	-2.93748	-0.69449	0.04521	-2.93752	-0.69647	0.00001
**C**	-2.83943	0.60655	-0.28609	-2.83904	0.62849	-0.21652	-2.83877	0.64428	-0.14530	-2.83860	0.65381	-0.07298	-2.83855	0.65700	-0.00011
**H**	-3.82238	-1.24117	0.39272	-3.82291	-1.27129	0.29719	-3.82329	-1.29298	0.19939	-3.82351	-1.30605	0.10008	-3.82359	-1.31043	0.00003
**H**	-3.53840	1.37628	-0.56183	-3.53764	1.41937	-0.42520	-3.53710	1.45038	-0.28534	-3.53677	1.46909	-0.14330	-3.53667	1.47535	-0.00020
**N**	-1.66871	-1.18366	0.35809	-1.66919	-1.21112	0.27099	-1.66953	-1.23089	0.18184	-1.66974	-1.24281	0.09130	-1.66981	-1.24680	0.00008
**C**	0.55588	-0.23889	0.00003	0.55588	-0.23889	0.00003	0.55588	-0.23889	0.00003	0.55588	-0.23889	0.00003	0.55588	-0.23889	0.00003
**C**	2.71872	0.17436	0.16418	2.71894	0.18695	0.12424	2.71910	0.19601	0.08335	2.71920	0.20147	0.04183	2.71923	0.20329	0.00000
**C**	2.54557	-1.07897	-0.29298	2.54517	-1.10144	-0.22170	2.54489	-1.11762	-0.14873	2.54472	-1.12738	-0.07463	2.54466	-1.13064	0.00002
**H**	3.58934	0.76502	0.38462	3.58986	0.79450	0.29104	3.59023	0.81573	0.19524	3.59046	0.82852	0.09796	3.59054	0.83278	-0.00005
**H**	3.22391	-1.86502	-0.57471	3.22313	-1.90910	-0.43489	3.22257	-1.94083	-0.29177	3.22223	-1.95997	-0.14642	3.22212	-1.96637	0.00001
**N**	1.43019	0.71915	0.35420	1.43067	0.74631	0.26803	1.43101	0.76586	0.17982	1.43122	0.77765	0.09024	1.43129	0.78158	0.00000
**O**	1.19241	1.89239	0.77955	1.19346	1.95217	0.58989	1.19422	1.99520	0.39573	1.19467	2.02114	0.19857	1.19483	2.02980	-0.00007
**O**	-1.51247	0.91248	-0.40585	-1.51193	0.94361	-0.30714	-1.51154	0.96601	-0.20608	-1.51130	0.97952	-0.10346	-1.51122	0.98404	-0.00007
**O**	1.21685	-1.35052	-0.40015	1.21631	-1.38121	-0.30277	1.21592	-1.40329	-0.20308	1.21569	-1.41661	-0.10185	1.21561	-1.42105	0.00014
	**O3** 90°			**O3** 100°			**O3** 110°			**O3** 120°			**O3** 130°		
**C**	0.01945	0.70642	0.00000	0.01945	0.70642	0.00000	0.01945	0.70642	0.00000	0.01945	0.70642	0.00000	0.01945	0.70642	0.00000
**C**	0.46862	2.83224	0.39049	0.50115	2.83135	0.35497	0.53047	2.83054	0.31675	0.55634	2.82983	0.27612	0.57858	2.82922	0.23338
**C**	-0.48285	2.75091	-0.55838	-0.52938	2.75219	-0.50759	-0.57130	2.75335	-0.45294	-0.60830	2.75437	-0.39484	-0.64010	2.75524	-0.33373
**H**	0.96569	3.65905	0.86462	1.03773	3.65707	0.78597	1.10264	3.65528	0.70134	1.15994	3.65370	0.61138	1.20917	3.65235	0.51676
**H**	-1.03031	3.48251	-1.12577	-1.12411	3.48509	-1.02337	-1.20863	3.48742	-0.91318	-1.28323	3.48947	-0.79604	-1.34734	3.49124	-0.67284
**C**	-0.01945	-0.70642	0.00000	-0.01945	-0.70642	0.00000	-0.01945	-0.70642	0.00000	-0.01945	-0.70642	0.00000	-0.01945	-0.70642	0.00000
**C**	-0.46862	-2.83224	0.39049	-0.50115	-2.83135	0.35497	-0.53047	-2.83054	0.31675	-0.55634	-2.82983	0.27612	-0.57858	-2.82922	0.23338
**C**	0.48285	-2.75091	-0.55838	0.52938	-2.75219	-0.50759	0.57130	-2.75335	-0.45294	0.60830	-2.75437	-0.39484	0.64010	-2.75524	-0.33373
**H**	-0.96569	-3.65905	0.86462	-1.03773	-3.65707	0.78597	-1.10264	-3.65528	0.70134	-1.15994	-3.65370	0.61138	-1.20917	-3.65235	0.51676
**H**	1.03031	-3.48251	-1.12577	1.12411	-3.48509	-1.02337	1.20863	-3.48742	-0.91318	1.28323	-3.48947	-0.79604	1.34734	-3.49124	-0.67284
**N**	0.79589	1.51212	0.75397	0.85871	1.51039	0.68539	0.91531	1.50883	0.61159	0.96527	1.50745	0.53314	1.00821	1.50627	0.45063
**N**	-0.79589	-1.51212	0.75397	-0.85871	-1.51039	0.68539	-0.91531	-1.50883	0.61159	-0.96527	-1.50745	0.53314	-1.00821	-1.50627	0.45063

**O**	1.66093	1.17985	1.62783	1.79656	1.17611	1.47976	1.91877	1.17275	1.32043	2.02664	1.16978	1.15105	2.11934	1.16722	0.97291
**O**	-1.66093	-1.17985	1.62783	-1.79656	-1.17611	1.47976	-1.91877	-1.17275	1.32043	-2.02664	-1.16978	1.15105	-2.11934	-1.16722	0.97291
**O**	0.77206	-1.44403	-0.81151	0.83967	-1.44589	-0.73770	0.90060	-1.44757	-0.65827	0.95437	-1.44905	-0.57383	1.00059	-1.45032	-0.48502
**O**	-0.77206	1.44403	-0.81151	-0.83967	1.44589	-0.73770	-0.90060	1.44757	-0.65827	-0.95437	1.44905	-0.57383	-1.00059	1.45032	-0.48502
	**O3** 140°			**O3** 150°			**O3** 160°			**O3** 170°			**O3** 180°		
**C**	0.01945	0.70642	0.00000	0.01945	0.70642	0.00000	0.01945	0.70642	0.00000	0.01945	0.70642	0.00000	0.01945	0.70642	0.00000
**C**	0.59701	2.82871	0.18888	0.61149	2.82831	0.14293	0.62192	2.82802	0.09589	0.62820	2.82785	0.04813	0.63030	2.82779	0.00000
**C**	-0.66645	2.75597	-0.27008	-0.68716	2.75654	-0.20438	-0.70206	2.75695	-0.13713	-0.71105	2.75720	-0.06882	-0.71406	2.75728	0.00000
**H**	1.24998	3.65122	0.41821	1.28204	3.65034	0.31647	1.30512	3.64970	0.21233	1.31904	3.64932	0.10657	1.32369	3.64919	0.00000
**H**	-1.40047	3.49270	-0.54452	-1.44222	3.49385	-0.41206	-1.47227	3.49468	-0.27646	-1.49039	3.49518	-0.13876	-1.49644	3.49534	0.00000
**C**	-0.01945	-0.70642	0.00000	-0.01945	-0.70642	0.00000	-0.01945	-0.70642	0.00000	-0.01945	-0.70642	0.00000	-0.01945	-0.70642	0.00000
**C**	-0.59701	-2.82871	0.18888	-0.61149	-2.82831	0.14293	-0.62192	-2.82802	0.09589	-0.62820	-2.82785	0.04813	-0.63030	-2.82779	0.00000
**C**	0.66645	-2.75597	-0.27008	0.68716	-2.75654	-0.20438	0.70206	-2.75695	-0.13713	0.71105	-2.75720	-0.06882	0.71406	-2.75728	0.00000
**H**	-1.24998	-3.65122	0.41821	-1.28204	-3.65034	0.31647	-1.30512	-3.64970	0.21233	-1.31904	-3.64932	0.10657	-1.32369	-3.64919	0.00000
**H**	1.40047	-3.49270	-0.54452	1.44222	-3.49385	-0.41206	1.47227	-3.49468	-0.27646	1.49039	-3.49518	-0.13876	1.49644	-3.49534	0.00000
**N**	1.04379	1.50529	0.36469	1.07175	1.50452	0.27597	1.09188	1.50397	0.18516	1.10402	1.50363	0.09293	1.10807	1.50352	0.00000
**N**	-1.04379	-1.50529	0.36469	-1.07175	-1.50452	0.27597	-1.09188	-1.50397	0.18516	-1.10402	-1.50363	0.09293	-1.10807	-1.50352	0.00000
**O**	2.19616	1.16511	0.78736	2.25653	1.16345	0.59583	2.29998	1.16225	0.39975	2.32619	1.16153	0.20064	2.33494	1.16129	0.00000
**O**	-2.19616	-1.16511	0.78736	-2.25653	-1.16345	0.59583	-2.29998	-1.16225	0.39975	-2.32619	-1.16153	0.20064	-2.33494	-1.16129	0.00000
**O**	1.03889	-1.45138	-0.39252	1.06898	-1.45221	-0.29703	1.09064	-1.45280	-0.19929	1.10371	-1.45316	-0.10002	1.10807	-1.45328	0.00000
**O**	-1.03889	1.45138	-0.39252	-1.06898	1.45221	-0.29703	-1.09064	1.45280	-0.19929	-1.10371	1.45316	-0.10002	-1.10807	1.45328	0.00000

Table S5 – Total Molecular Energies (Hartrees) calculated with ccsd(t) 6-311++g(d,p) on the DFT optimized structures of 2,2'-bioxazole **O1**, **O2** and **O3**.

Angle (°)	**O1**	**O2**	**O3**
90	-488.2228535	-563.0043741	-637.7869715
100	-488.2230806	-563.0048065	-637.7877221
110	-488.2237822	-563.0055669	-637.7886273
120	-488.2248653	-563.006558	-637.7895533
130	-488.2261751	-563.0076576	-637.7903795
140	-488.2275251	-563.008748	-637.7910558

150	-488.2287406	-563.0097271	-637.7915741
160	-488.2296907	-563.0105091	-637.7919404
170	-488.2302934	-563.0110216	-637.7921662
180	-488.2305007	-563.0112026	-637.7922458

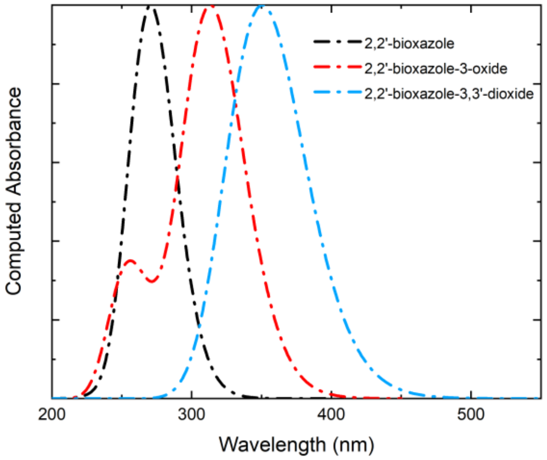

Figure S5 – Computed UV-Vis absorbance spectra of bioxazole **O1**, **O2** and **O3** from TD-DFT calculations (B3LYP/6-311++g(d,p)).

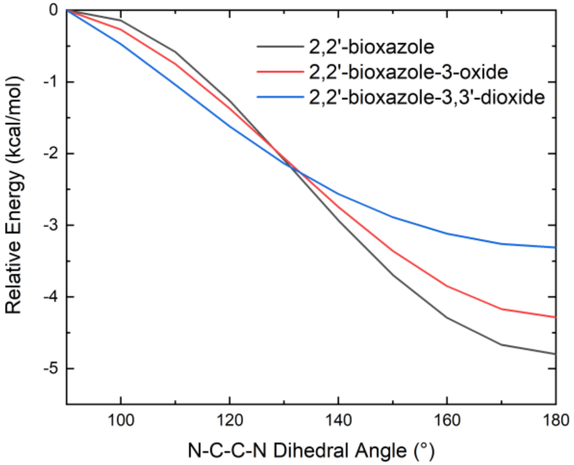

Figure S6 – Relative energies of **O1**, **O2** and **O3** for conformations from 90-180° (N-C-C-N bond) in 10° intervals. DFT optimization performed with B3LYP/6-311++g(d,p). CCSD(T) energy calculations performed with 6-311++g(d,p).

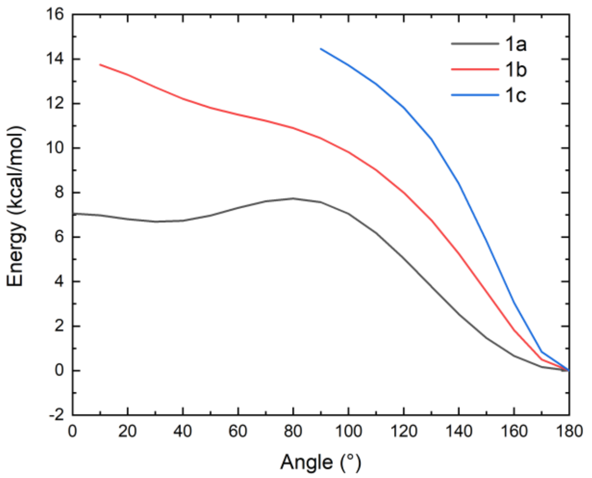

Figure S7 – Torsional barrier of **1a**, **1b**, and **1c** for conformations shown extended from 0 - 180° (N-C-C-N bond) in 10° intervals. Steric interactions of *N*-oxide with proton (**1b**) or second *N*-oxide (**1c**) cause large increases in barrier between 90 – 0°. DFT optimization performed with B3LYP/6-311++g(d,p). CCSD(T) energy calculations performed with 6-311++g(d,p).

Table S6 – Second order perturbative stabilization energies from Natural Bonding Orbital (pop=nbo) calculations

	**Donor**	**Acceptor**	**Energy (kcal/mol)**
**1b**	O15 (LP1)	BD*(1) S4 - C3	0.64
	O15 (LP2)	BD*(1) S4 - C3	2.70
**1c**	O15 (LP1)	BD*(1) S8 - C10	0.64
	O15 (LP2)	BD*(1) S8 - C10	3.11
	O16 (LP1)	BD*(1) S4 - C3	0.65
	O16 (LP2)	BD*(1) S4 - C3	3.09
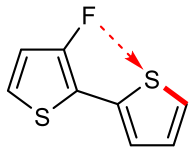	F16 (LP2)	BD*(1) S4 - C3	0.57

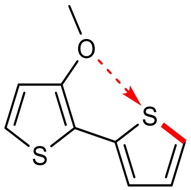	O16 (LP1)	BD*(1) S4 - C3	1.14
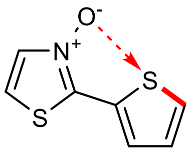	O14 (LP1)	BD*(1) S4 - C3	0.66
	O14 (LP2)	BD*(1) S4 - C3	2.87

Table S7 – QTAIM Calculation Summary

**Compound**	□ / a.u.	∇^2□□ / a.u.
**1b**	0.0199	+0.0622
**1c**	0.0210	+0.0642
	0.0210	+0.0642
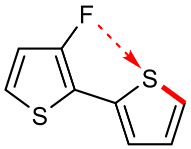	0.0105	+0.0401
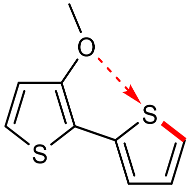	0.0209	+0.0650
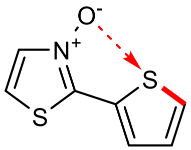	0.0133	+0.0471

Table S8 – QTAIM Calculation Structures

Compound	Charge Densities (□□	Laplacians (∇^2□□□
**1b**	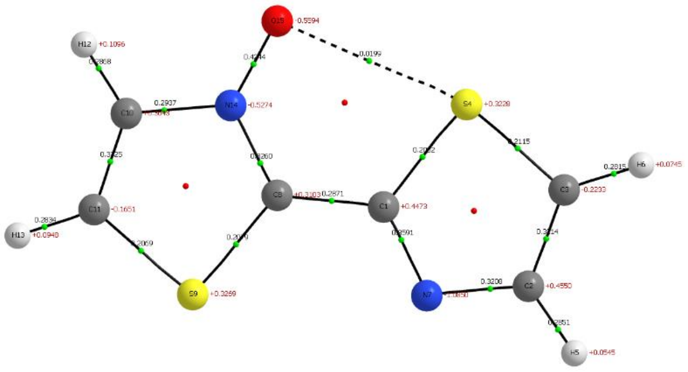	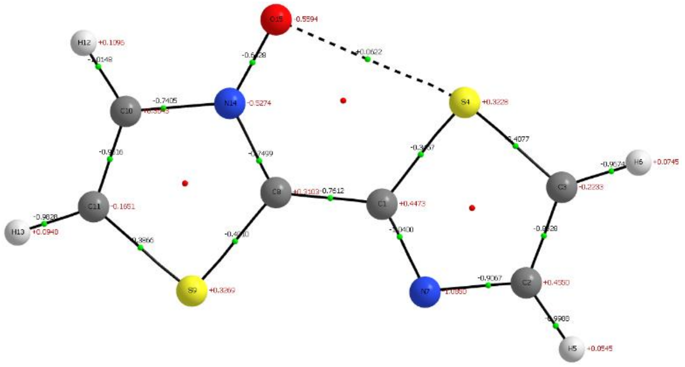
**1c**	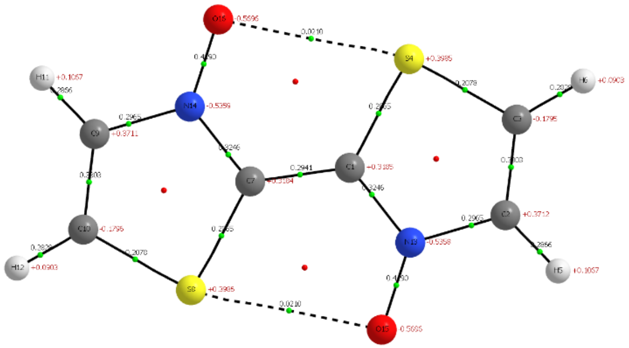	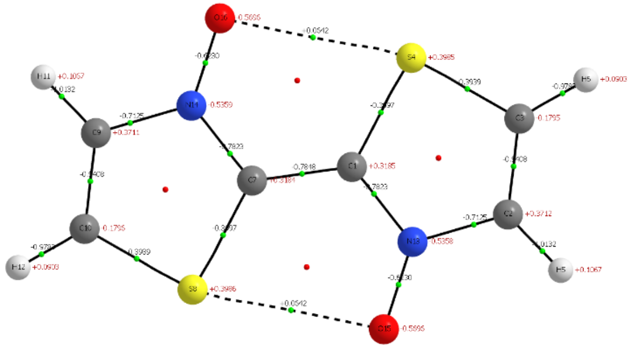
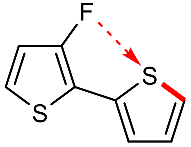	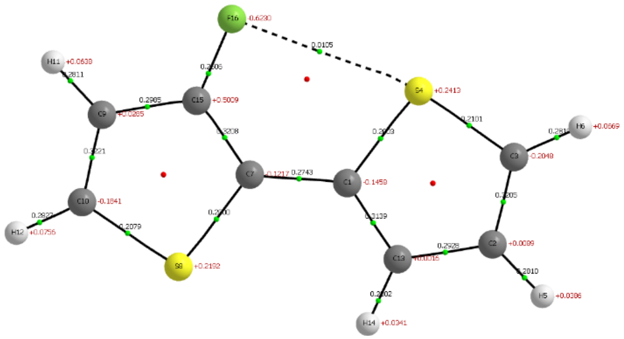	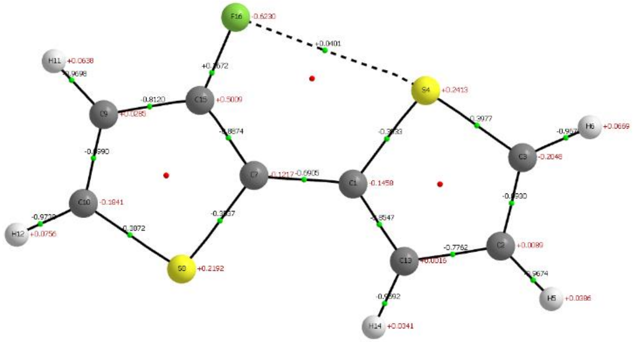

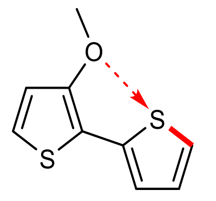	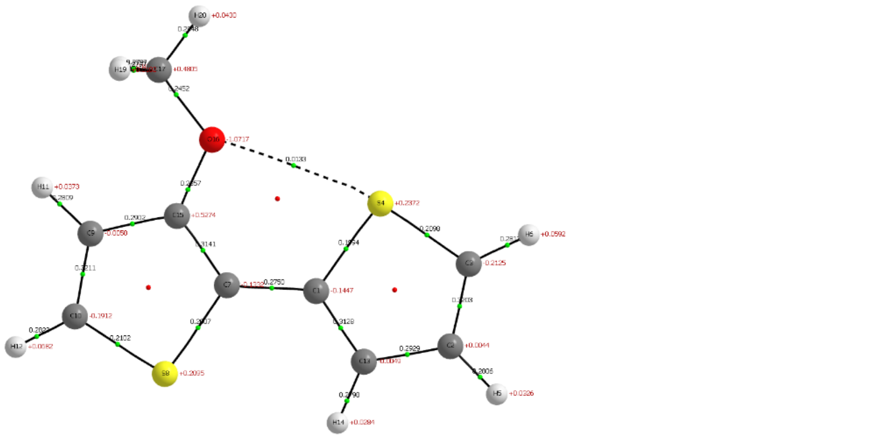	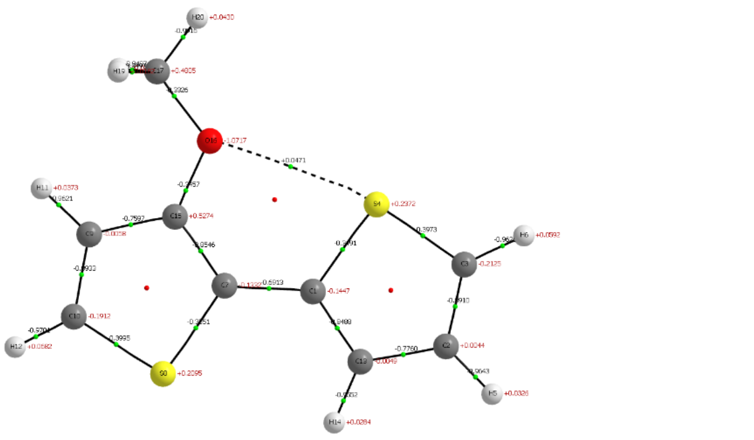
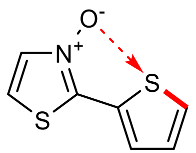	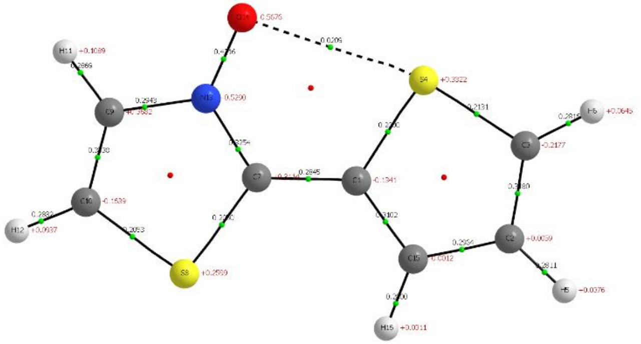	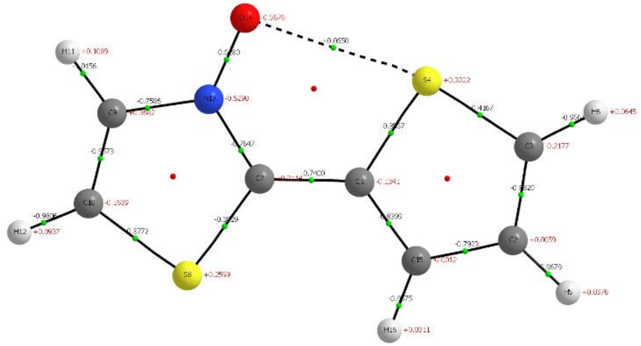

10. Spectral Data

1a

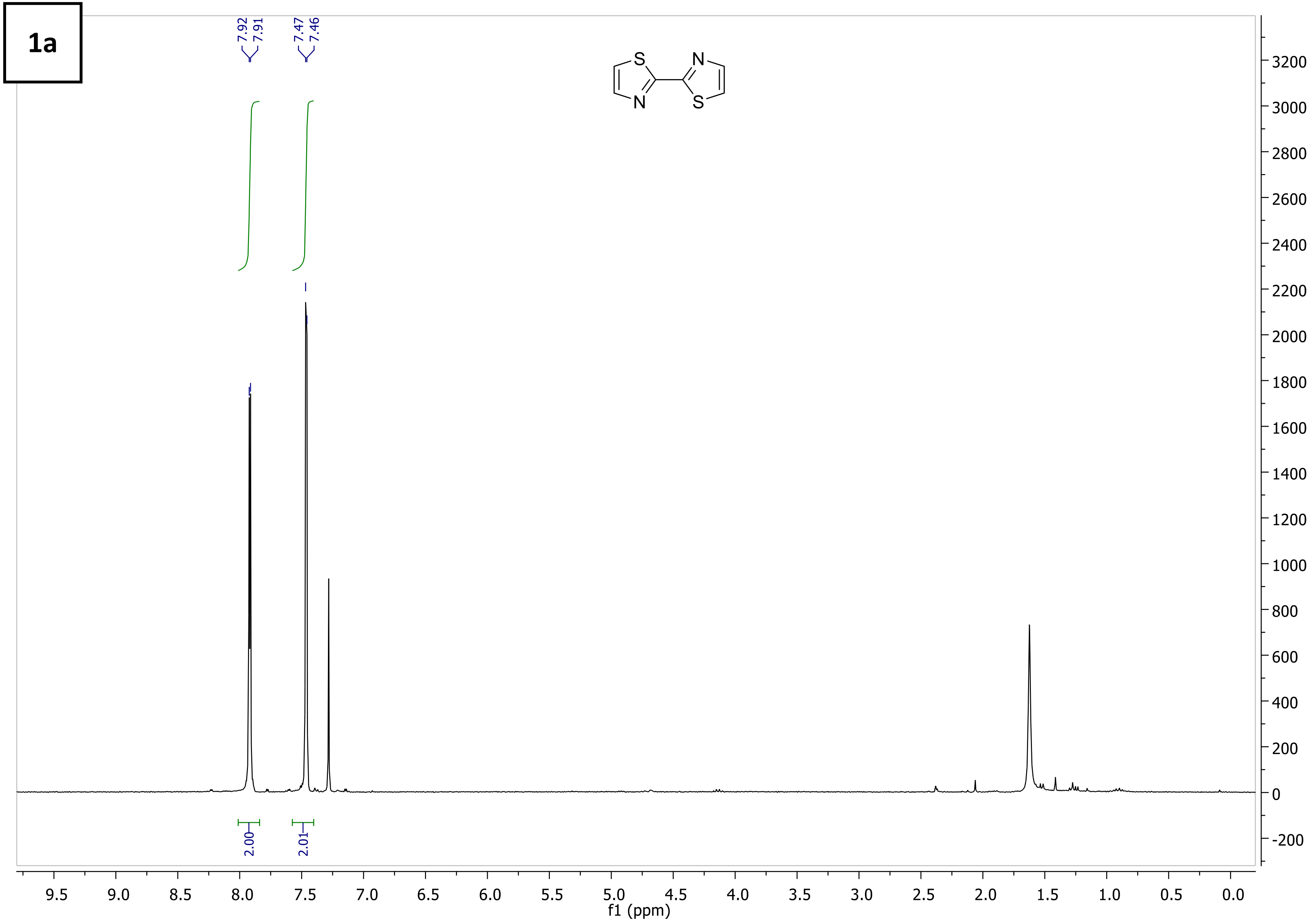

1a

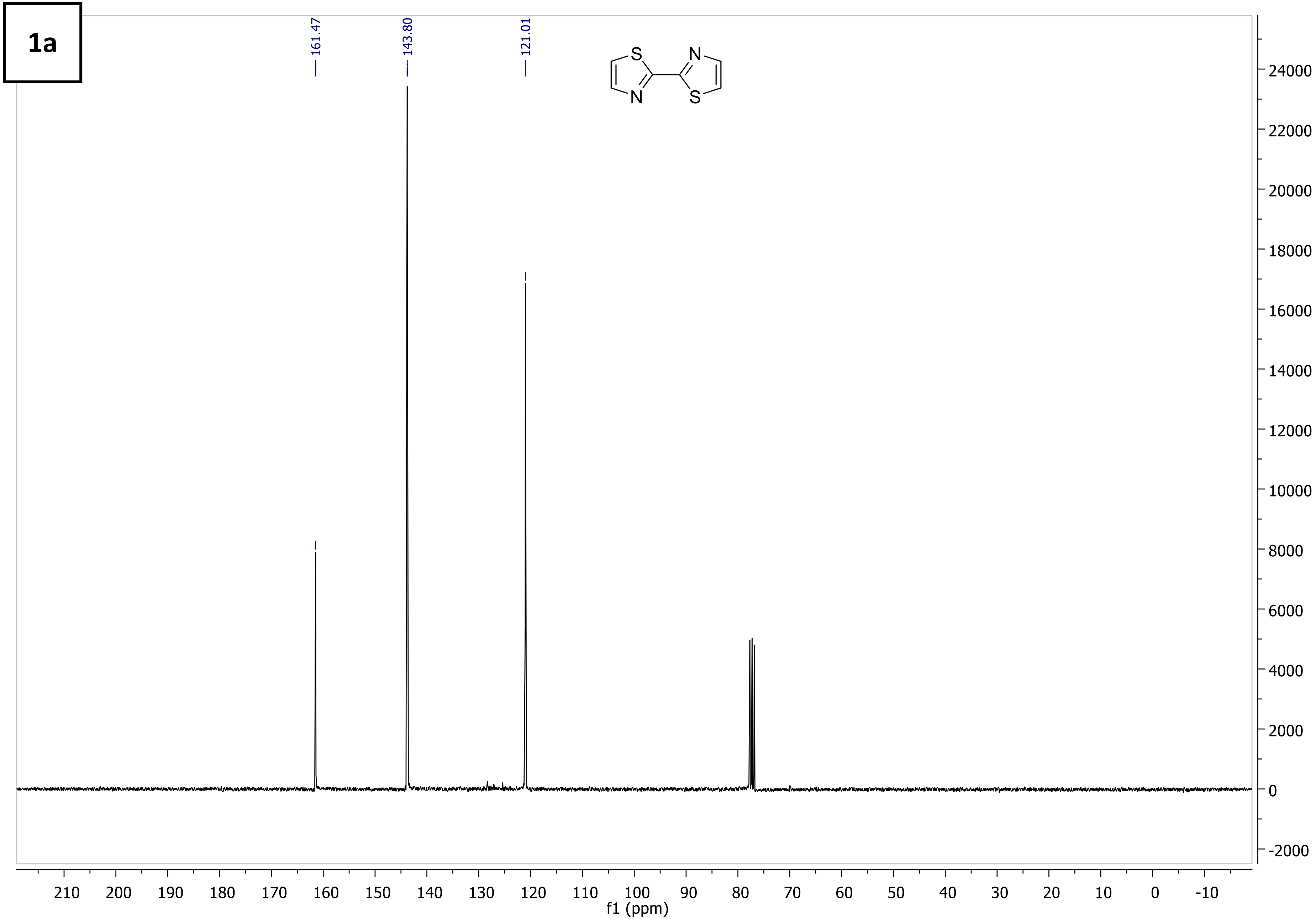
161.47
143.80
121.01
S
N
N
S
24000
22000
20000
18000
16000
14000
12000
10000
8000
6000
4000
2000
0
-2000
210 200 190 180 170 160 150 140 130 120 110 100 90 80 70 60 50 40 30 20 10 0 -10
f1 (ppm)

1b

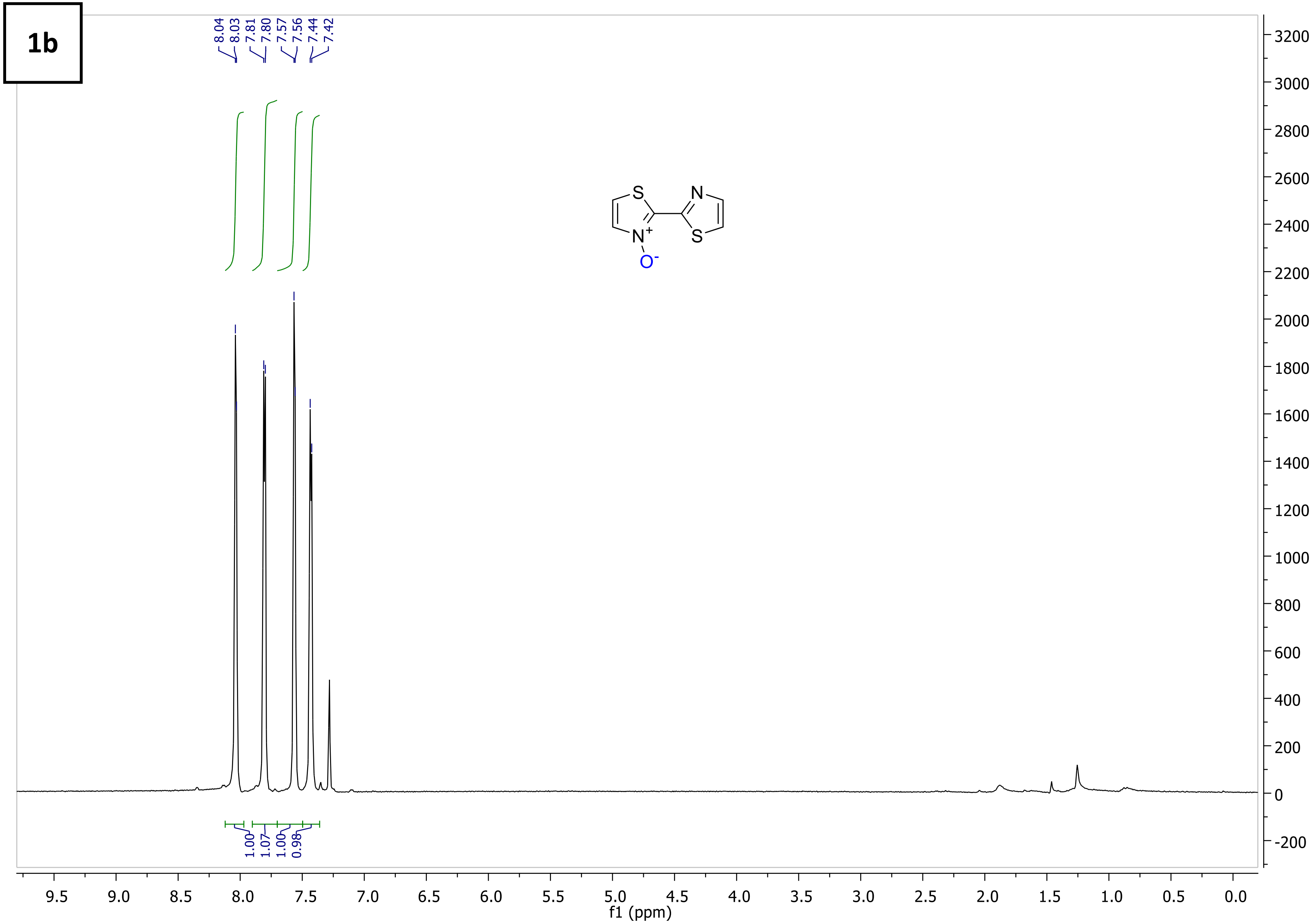

1b

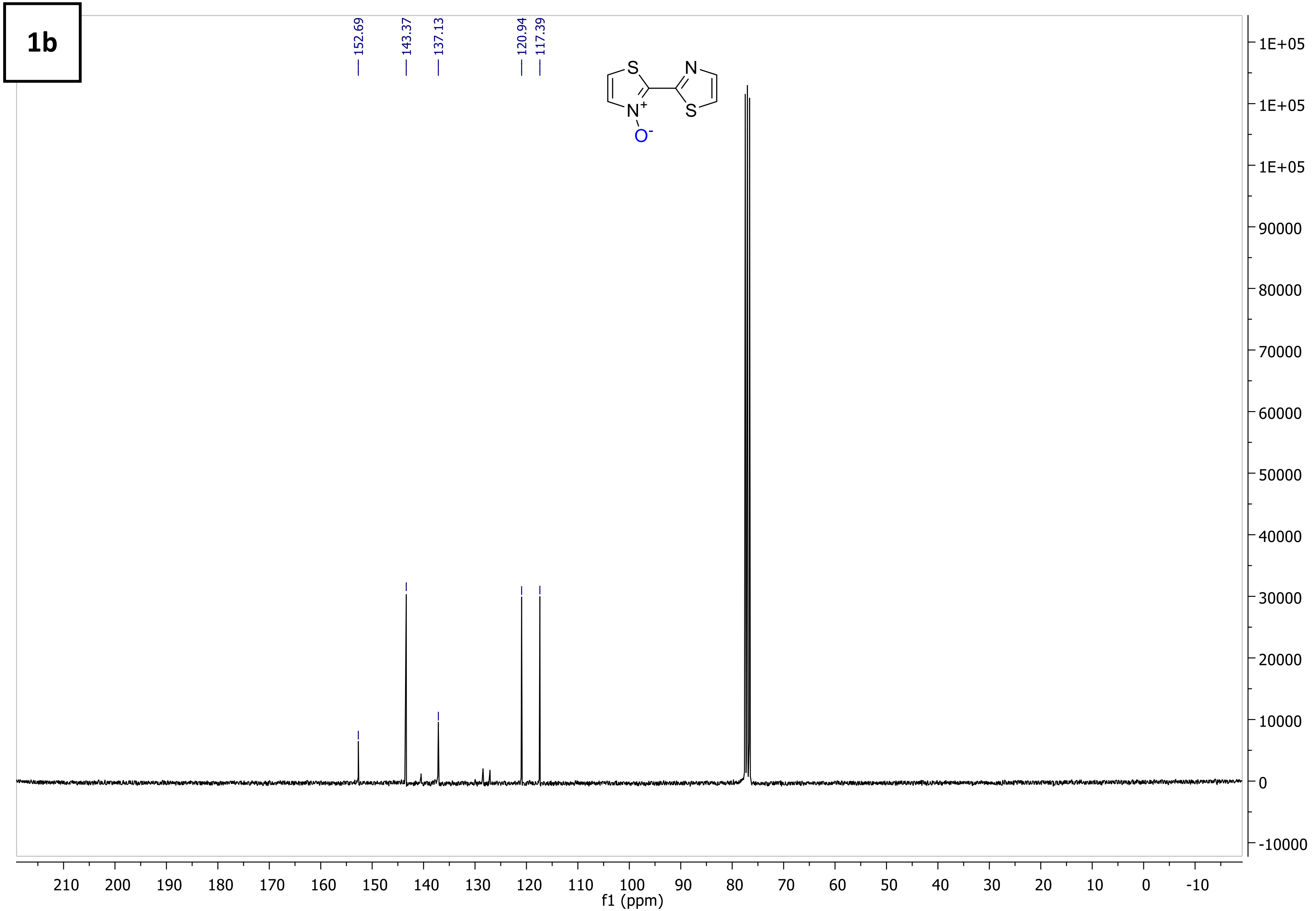
152.69
143.37
137.13
120.94
117.39
S
N
N+
O-
S
1E+05
1E+05
1E+05
90000
80000
70000
60000
50000
40000
30000
20000
10000
0
-10000
210 200 190 180 170 160 150 140 130 120 110 100 90 80 70 60 50 40 30 20 10 0 -10
f1 (ppm)

1c

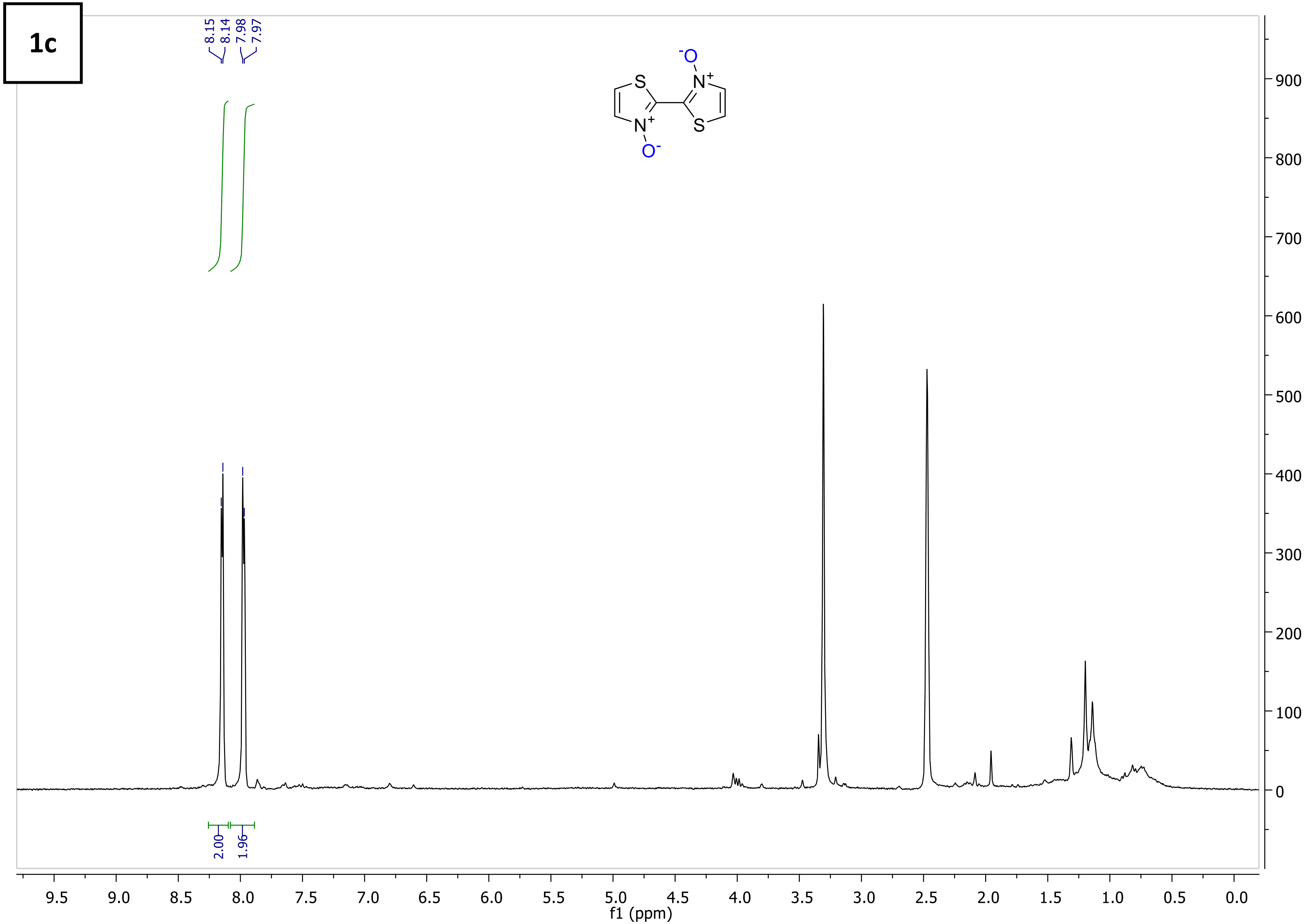

1c

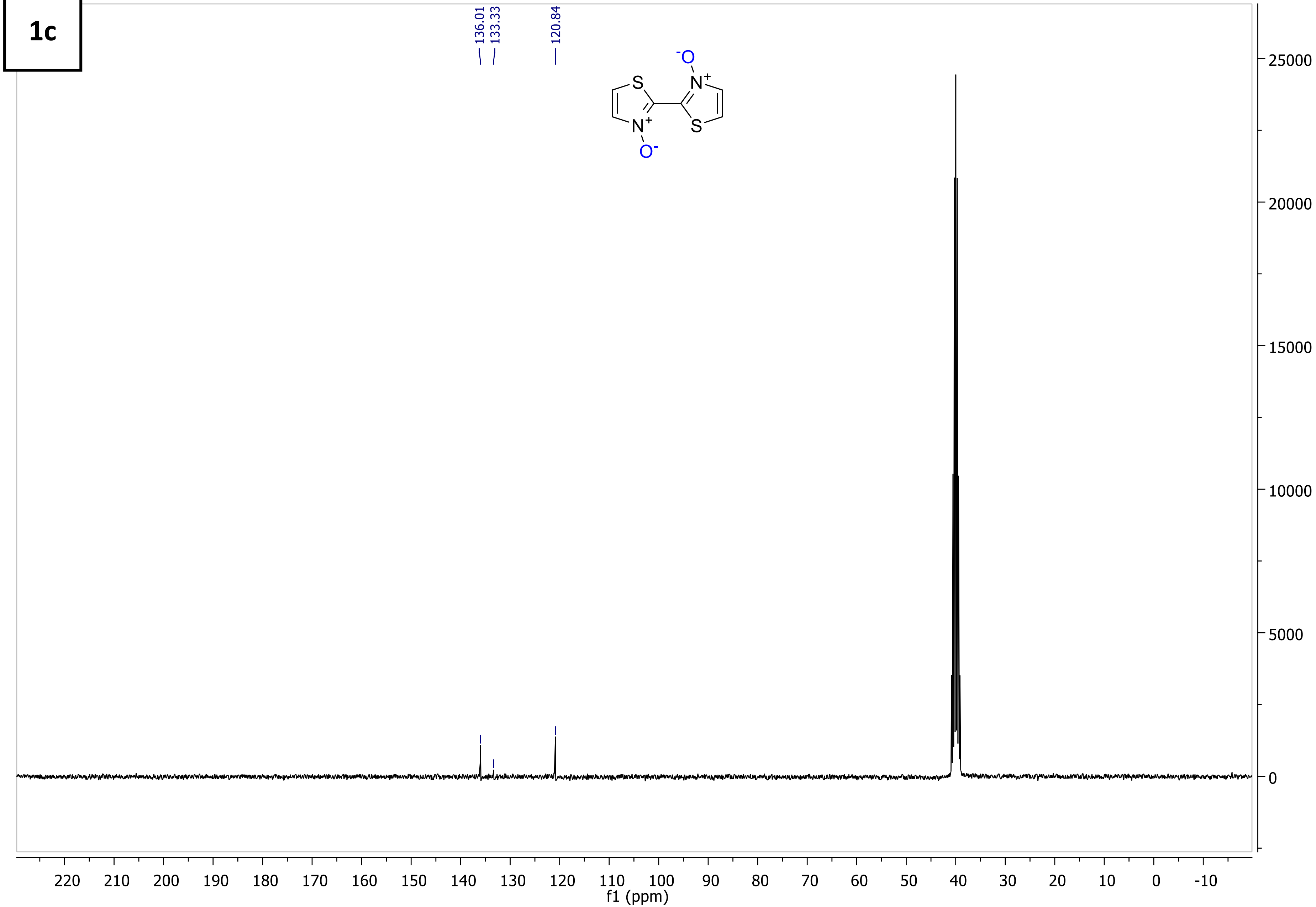
136.01
133.33
120.84
S
N+
O-
25000
20000
15000
10000
5000
0
220 210 200 190 180 170 160 150 140 130 120 110 100 90 80 70 60 50 40 30 20 10 0 -10
f1 (ppm)

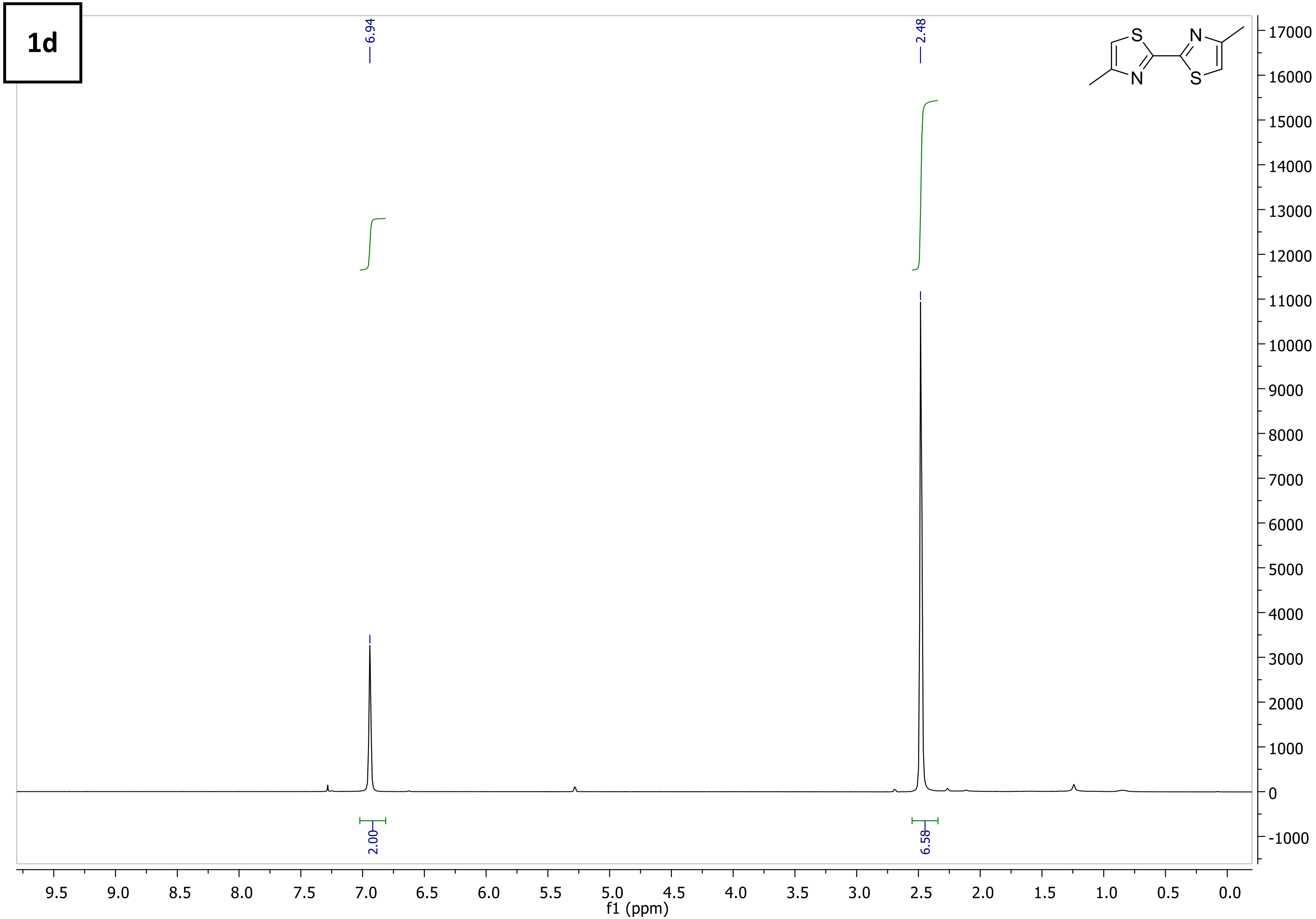
1d
6.94
2.48
S
N
N
S
2.00
6.58
f1 (ppm)

1d

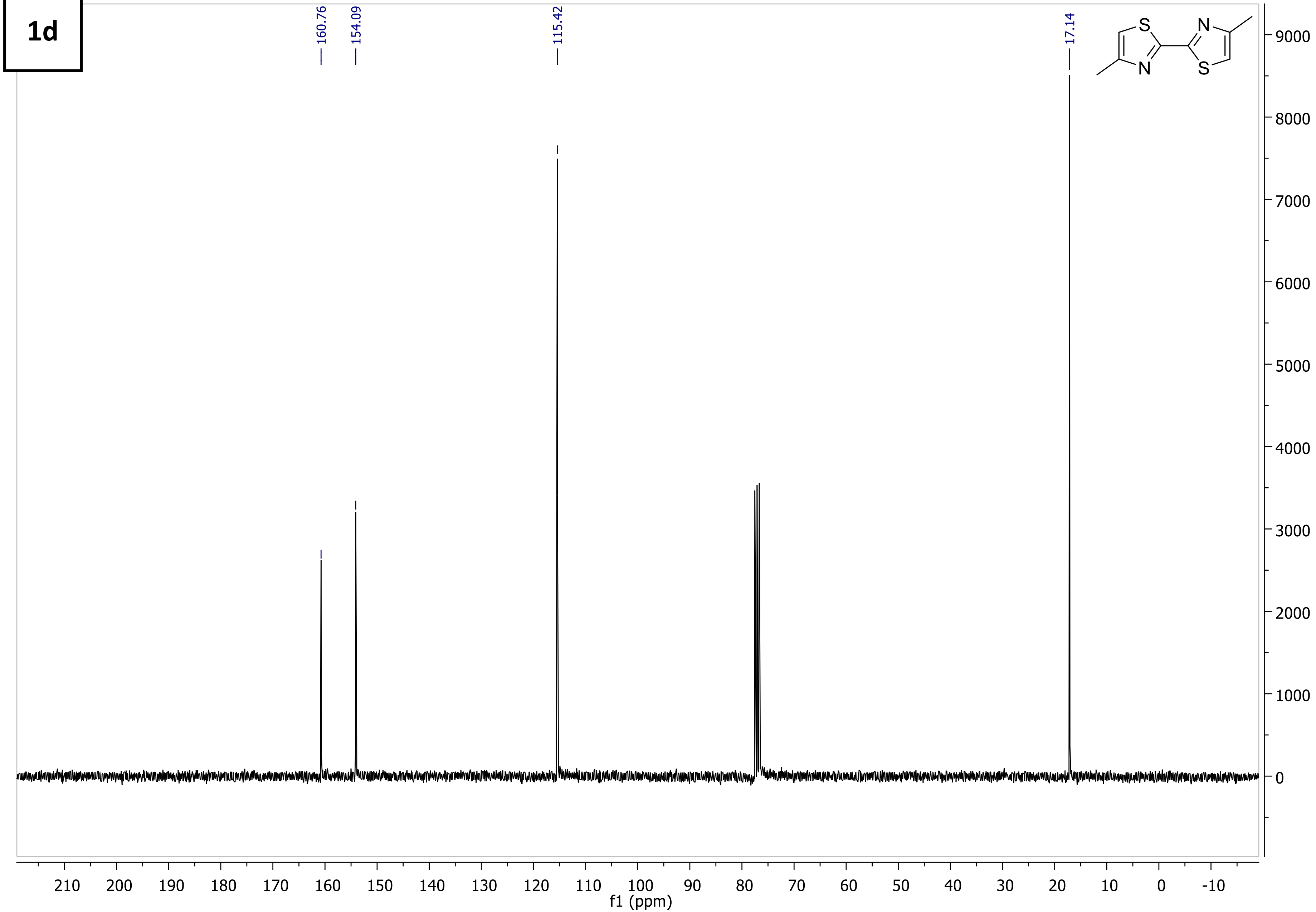

1e

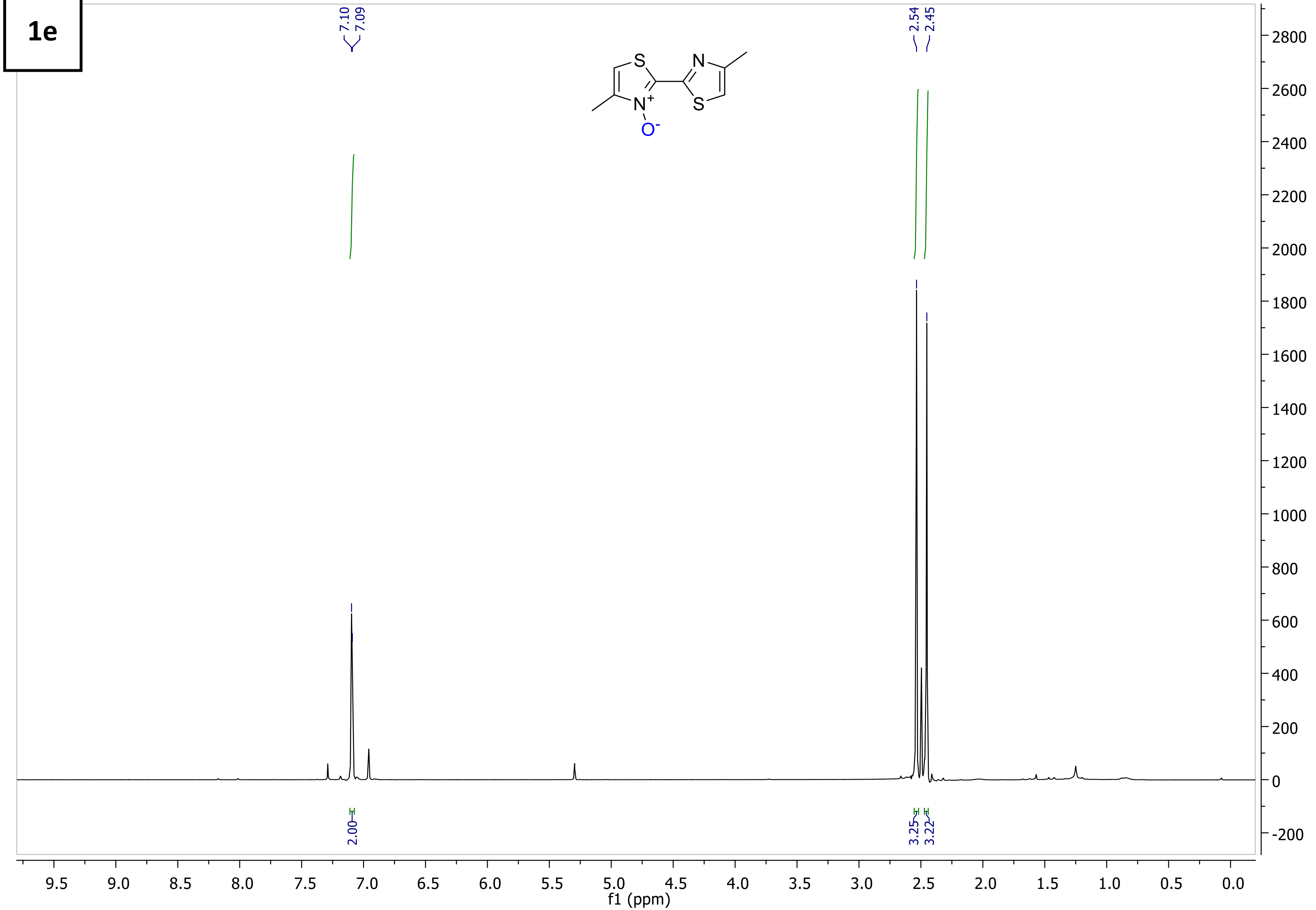
7.10
7.09
2.54
2.45
S
N
N+
O-
2.00
3.25
3.22
9.5 9.0 8.5 8.0 7.5 7.0 6.5 6.0 5.5 5.0 4.5 4.0 3.5 3.0 2.5 2.0 1.5 1.0 0.5 0.0
f1 (ppm)
2800 2600 2400 2200 2000 1800 1600 1400 1200 1000 800 600 400 200 0 -200

1e

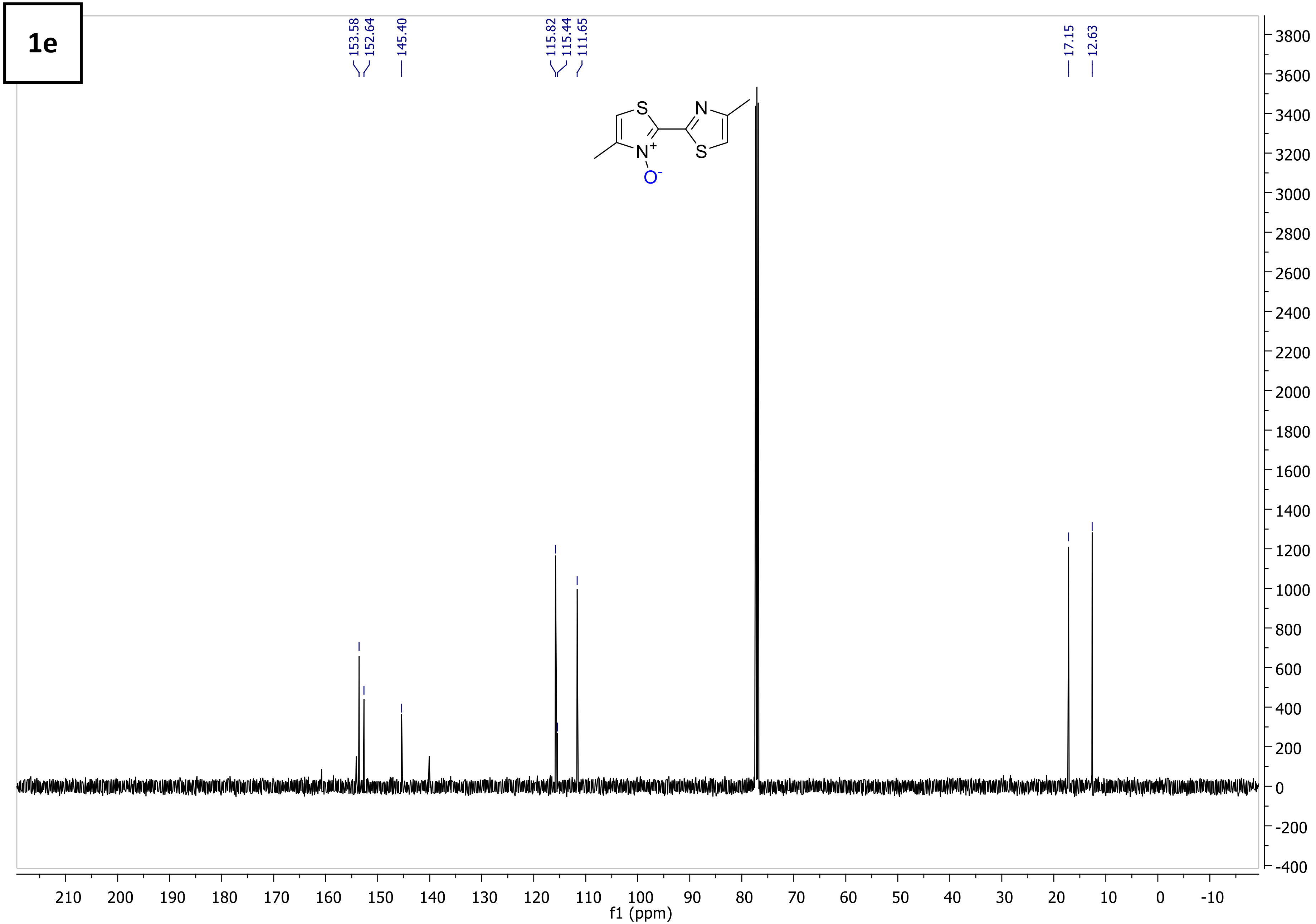
153.58
152.64
145.40
115.82
115.44
111.65
17.15
12.63
S
N
N+
O-
S
f1 (ppm)
210 200 190 180 170 160 150 140 130 120 110 100 90 80 70 60 50 40 30 20 10 0 -10
3800 3600 3400 3200 3000 2800 2600 2400 2200 2000 1800 1600 1400 1200 1000 800 600 400 200 0 -200 -400

1f

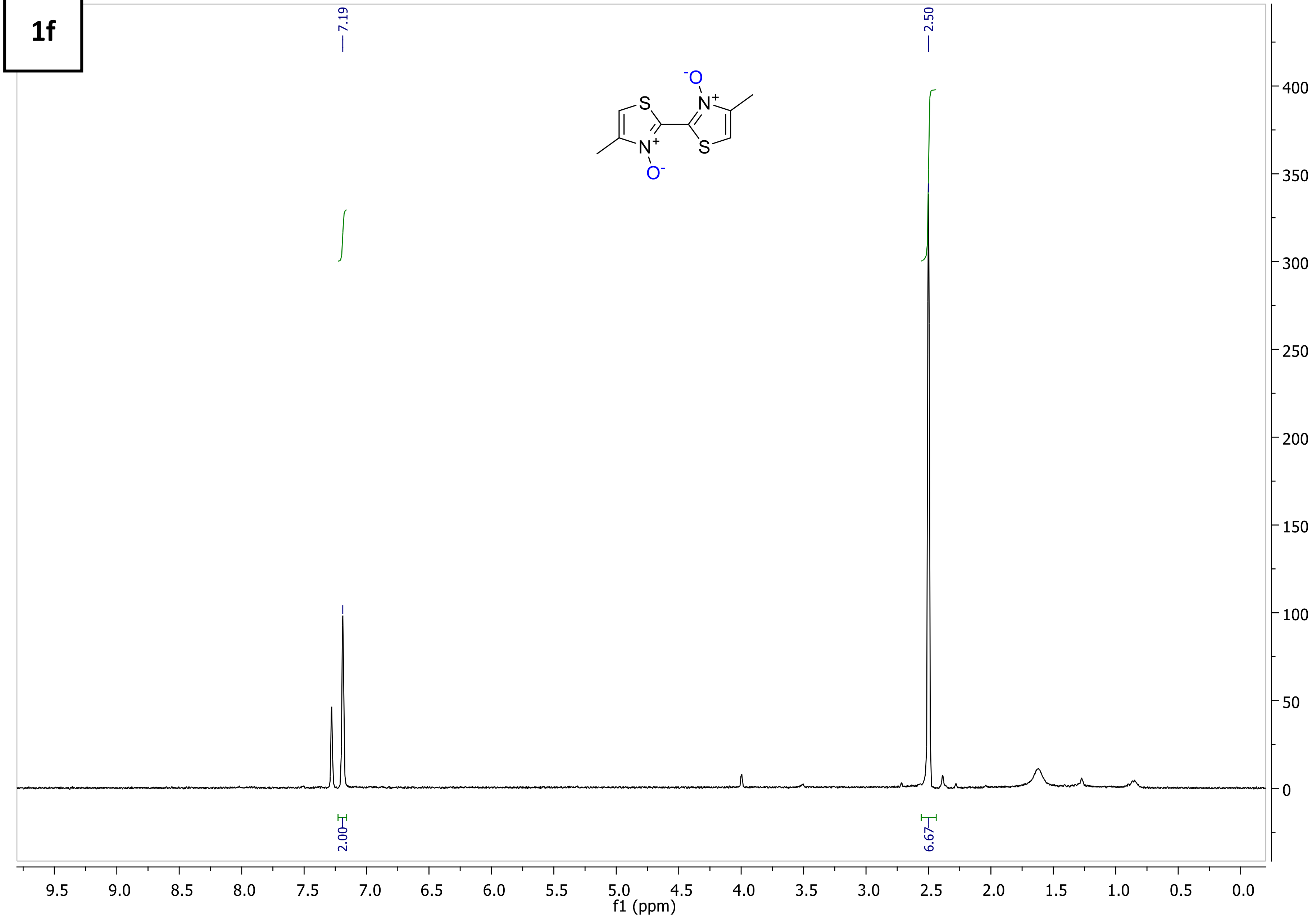

7.19
2.50
S
N+
O-
400
350
300
250
200
150
100
50
0
2.00
6.67
9.5
9.0
8.5
8.0
7.5
7.0
6.5
6.0
5.5
5.0
4.5
4.0
3.5
3.0
2.5
2.0
1.5
1.0
0.5
0.0
f1 (ppm)

1f

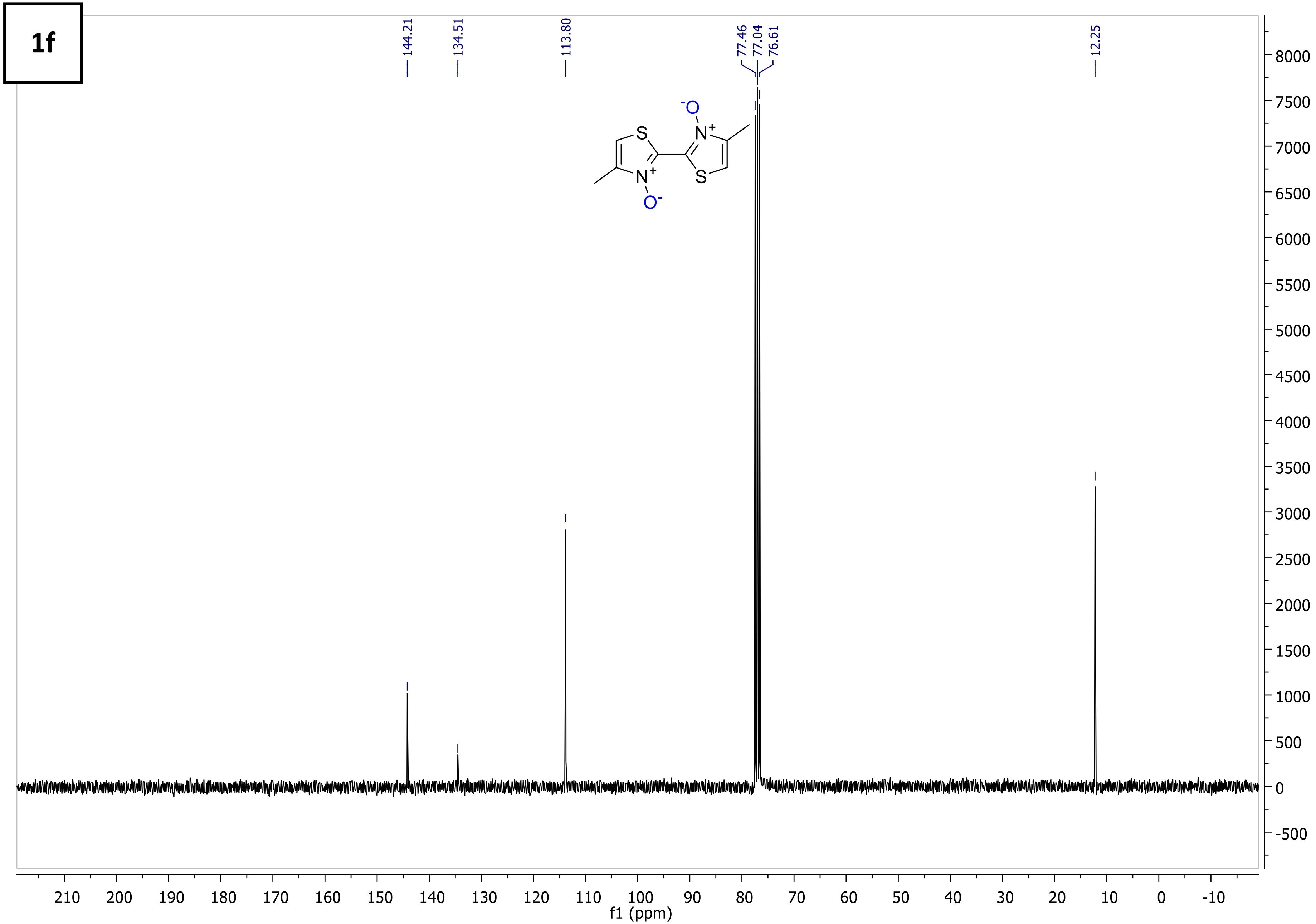
144.21
134.51
113.80
77.46
77.04
76.61
12.25
8000
7500
7000
6500
6000
5500
5000
4500
4000
3500
3000
2500
2000
1500
1000
500
0
-500
210
200
190
180
170
160
150
140
130
120
110
100
90
80
70
60
50
40
30
20
10
0
-10
f1 (ppm)

2a

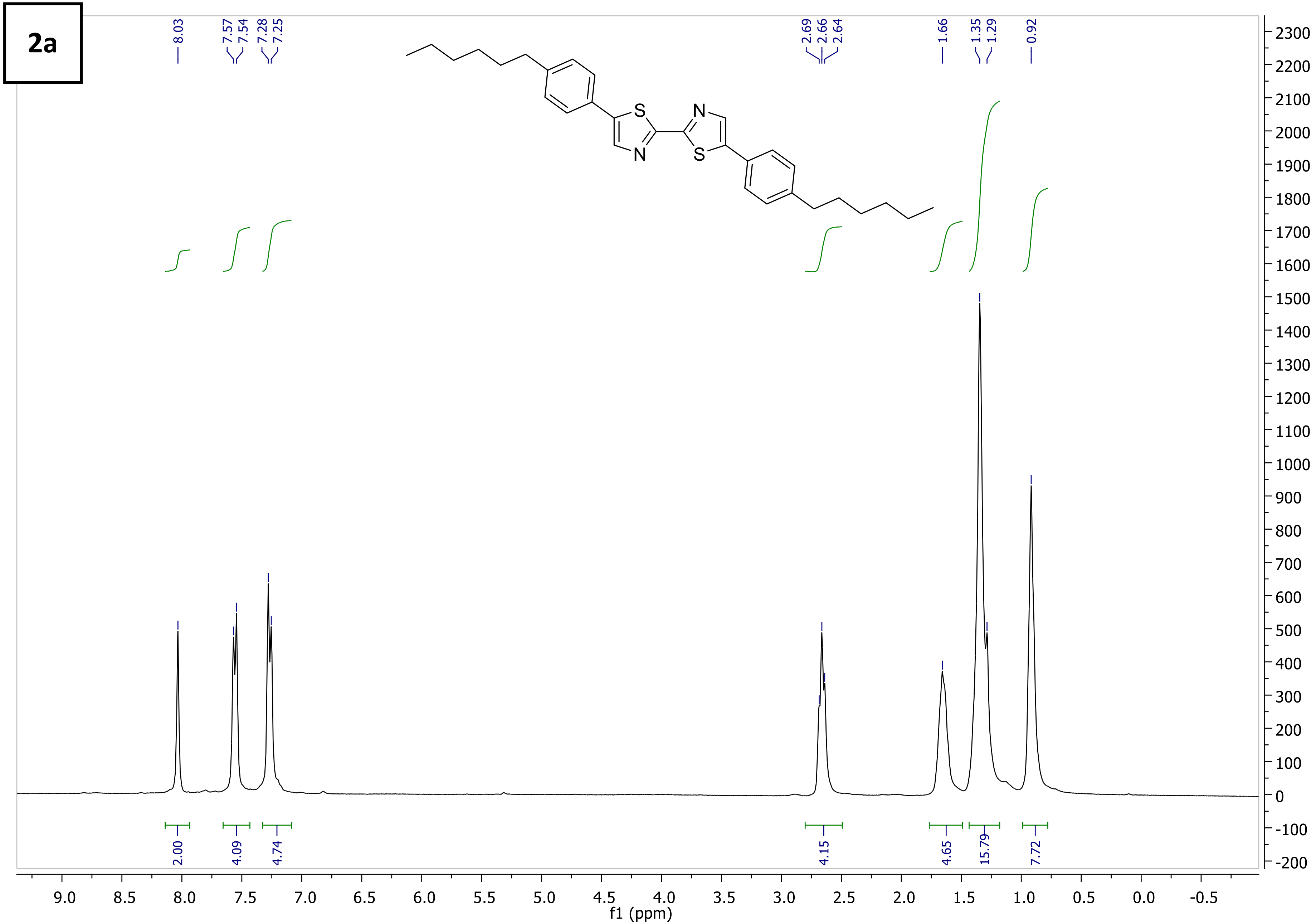
8.03
7.57
7.54
7.28
7.25
2.69
2.66
2.64
1.66
1.35
1.29
0.92
S
N
N
S
2.00
4.09
4.74
4.15
4.65
15.79
7.72
9.0 8.5 8.0 7.5 7.0 6.5 6.0 5.5 5.0 4.5 4.0 3.5 3.0 2.5 2.0 1.5 1.0 0.5 0.0 -0.5
f1 (ppm)
2300 2200 2100 2000 1900 1800 1700 1600 1500 1400 1300 1200 1100 1000 900 800 700 600 500 400 300 200 100 0 -100 -200

2a

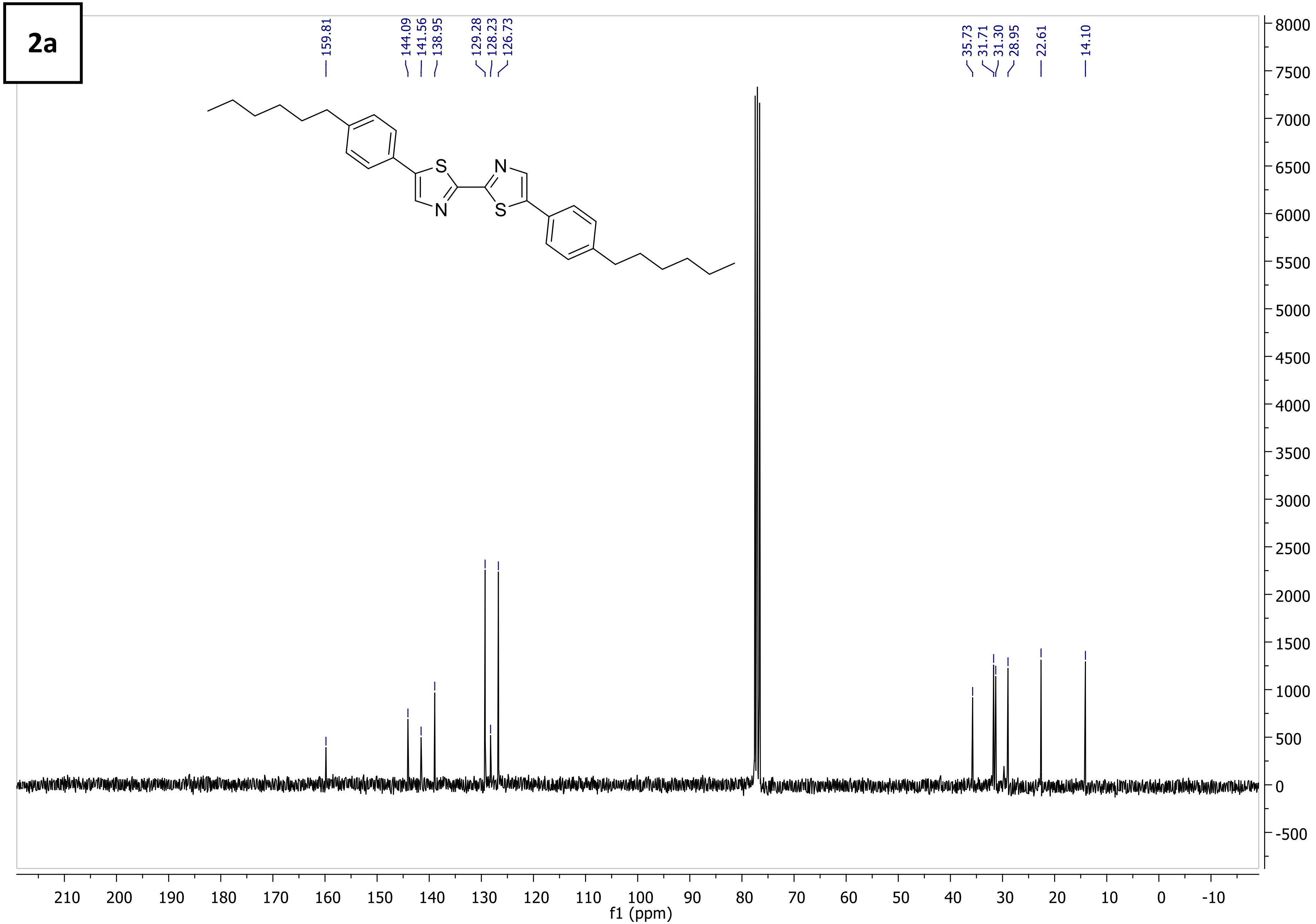
159.81
144.09
141.56
138.95
129.28
128.23
126.73
35.73
31.71
31.30
28.95
22.61
14.10
S
N
N
S
f1 (ppm)

2b

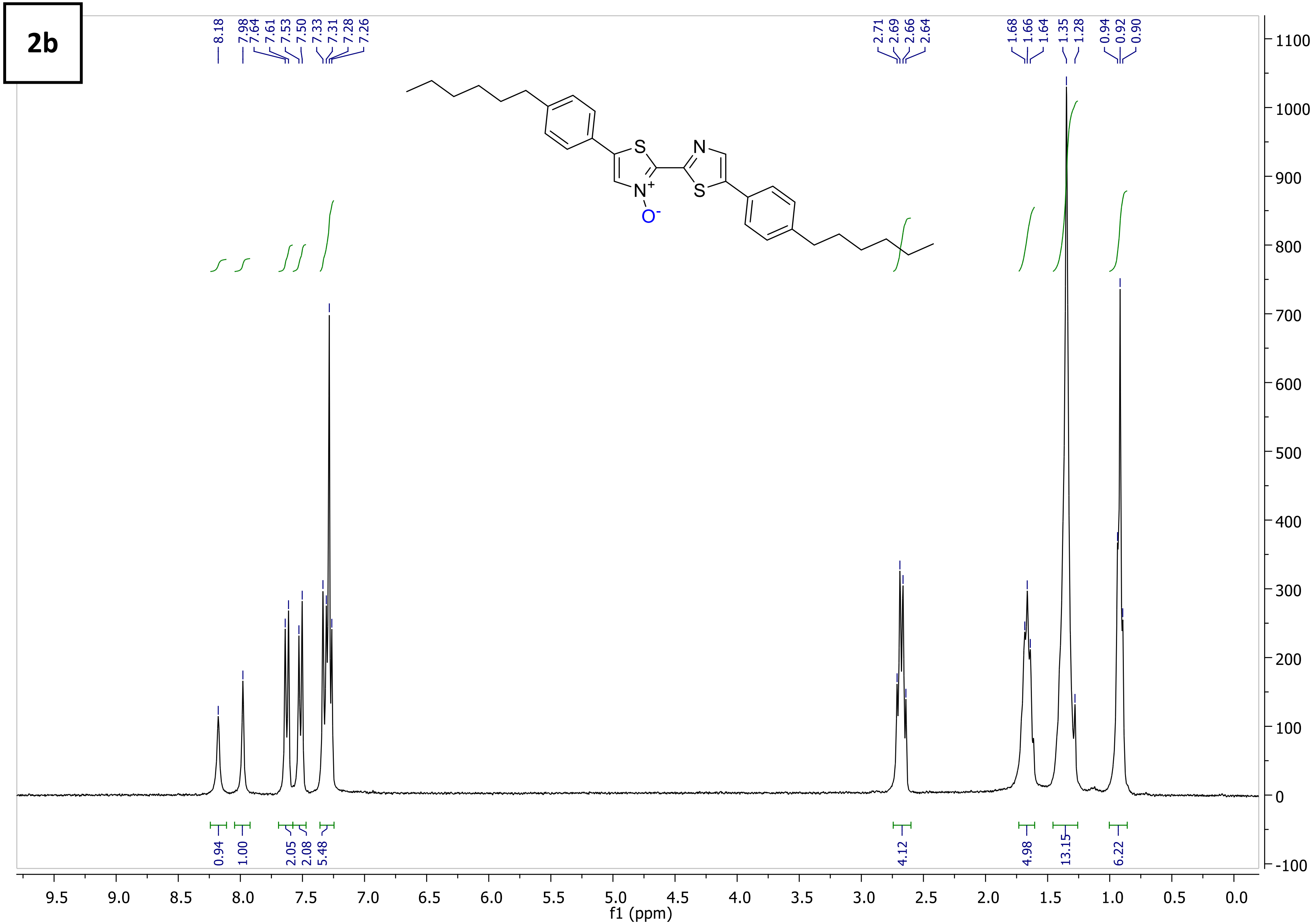
8.18
7.98
7.64
7.61
7.53
7.50
7.33
7.31
7.28
7.26
2.71
2.69
2.66
2.64
1.68
1.66
1.64
1.35
1.28
0.94
0.92
0.90
S
N
N+
S
O-
0.94
1.00
2.05
2.08
5.48
4.12
4.98
13.15
6.22
f1 (ppm)

2b

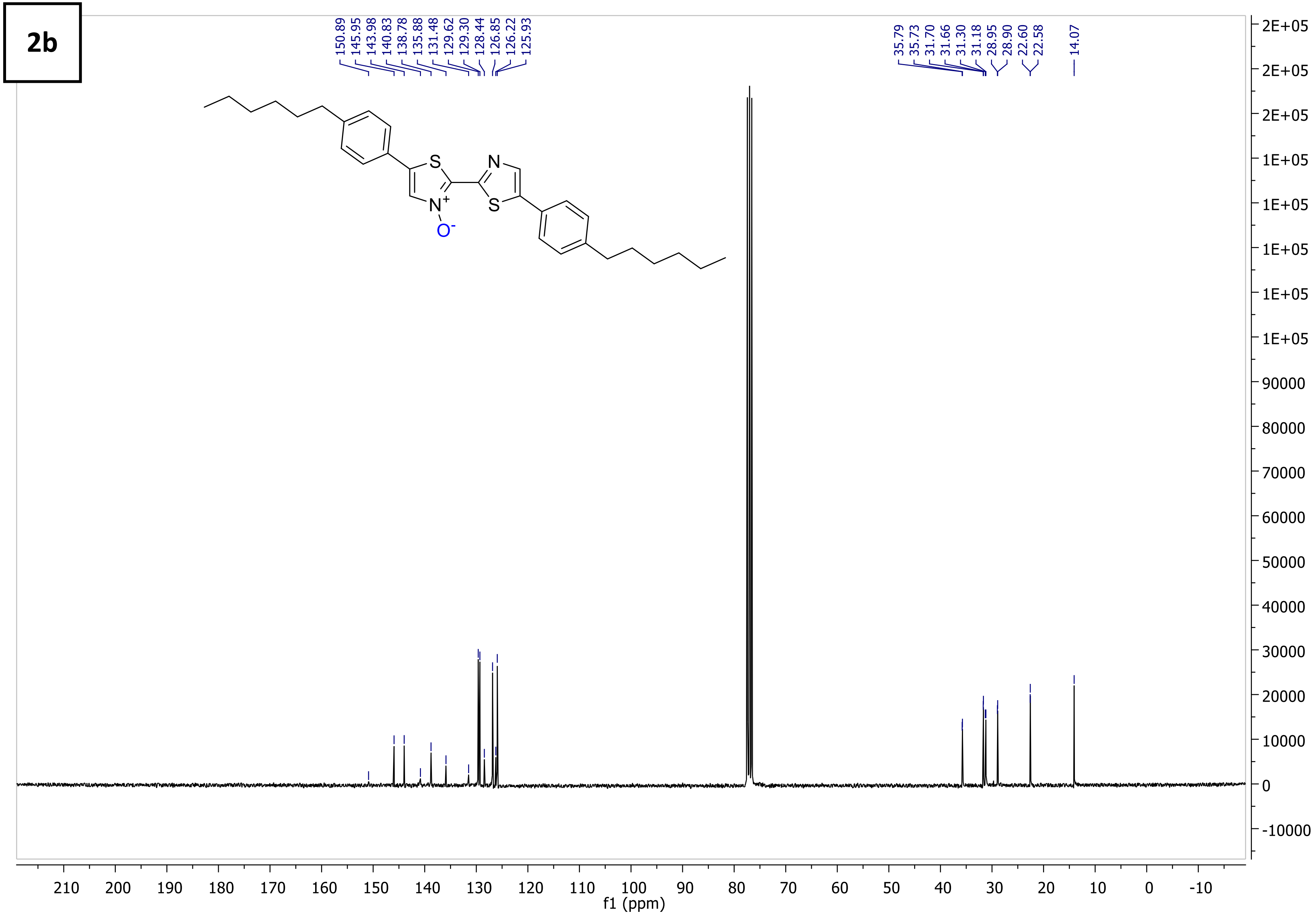

150.89
145.95
143.98
140.83
138.78
135.88
131.48
129.62
129.30
128.44
126.85
126.22
125.93
35.79
35.73
31.70
31.66
31.30
31.18
28.95
28.90
22.60
22.58
14.07
S
N
N+
O-
S
f1 (ppm)

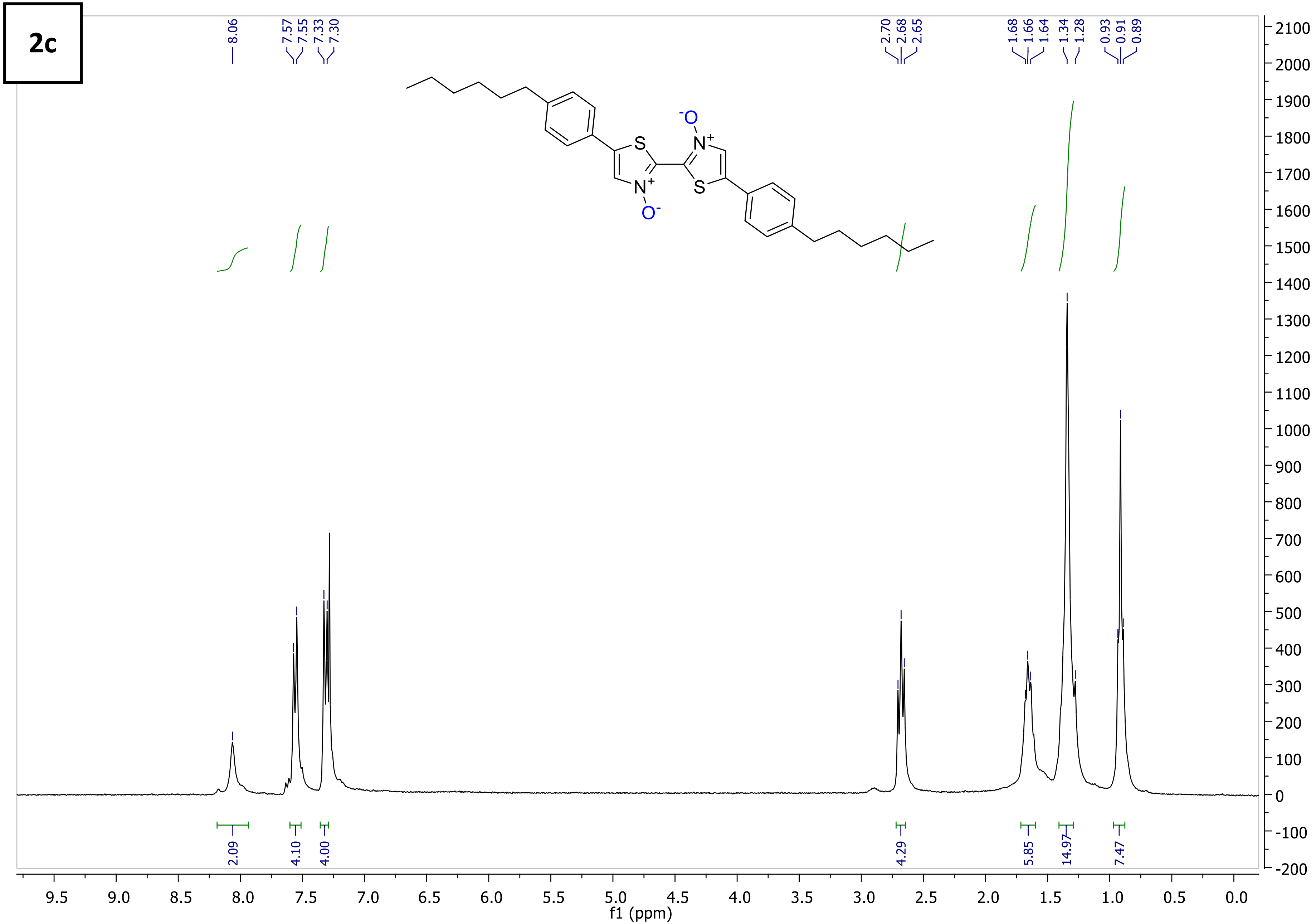
2c
8.06
7.57
7.55
7.33
7.30
2.70
2.68
2.65
1.68
1.66
1.64
1.34
1.28
0.93
0.91
0.89
2.09
4.10
4.00
4.29
5.85
14.97
7.47
f1 (ppm)

2c

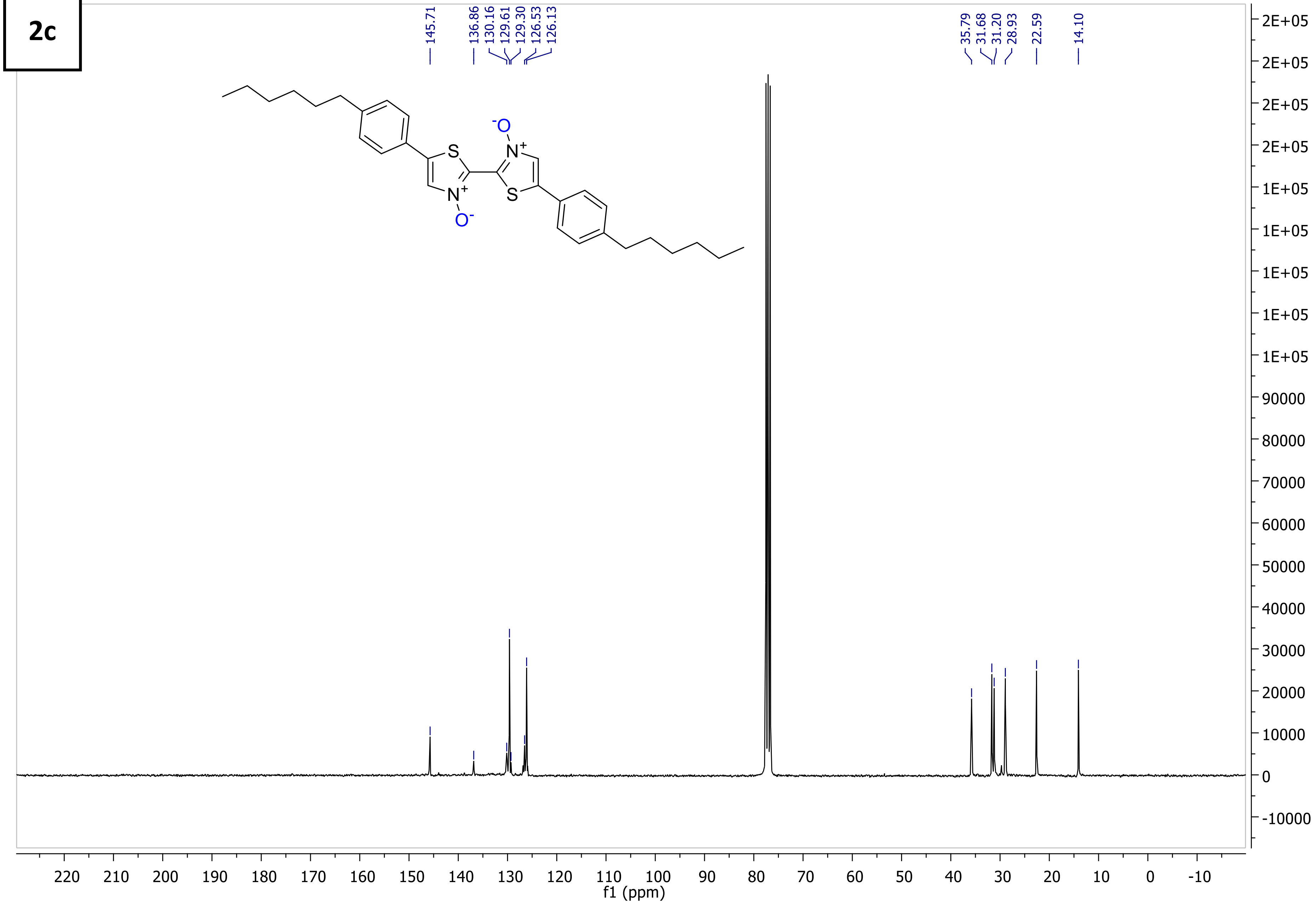
145.71
136.86
130.16
129.61
129.30
126.53
126.13
35.79
31.68
31.20
28.93
22.59
14.10
f1 (ppm)

3a

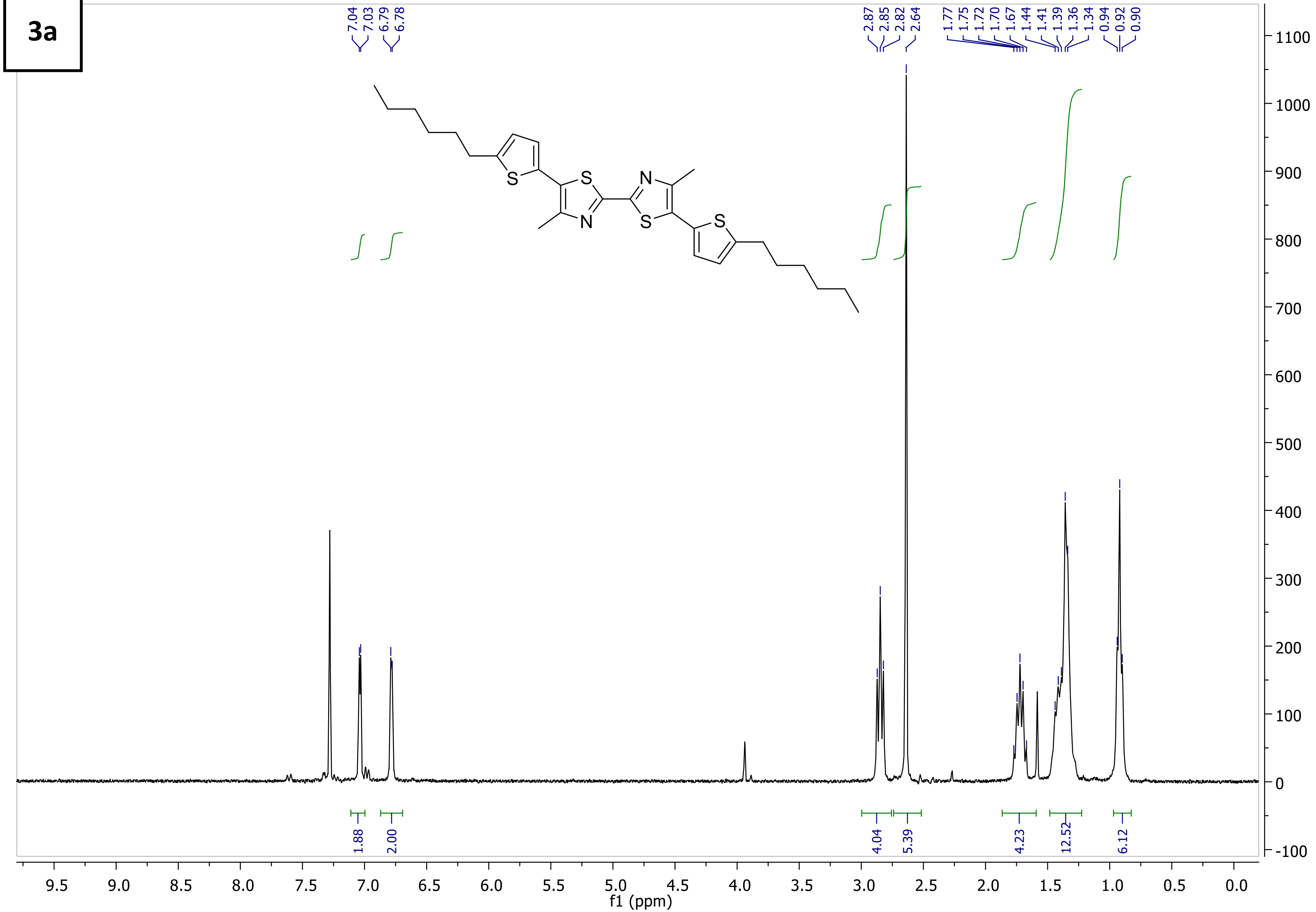

7.04
7.03
6.79
6.78
2.87
2.85
2.82
2.64
1.77
1.75
1.72
1.70
1.67
1.44
1.41
1.39
1.36
1.34
0.94
0.92
0.90
S
N
1.88
2.00
4.04
5.39
4.23
12.52
6.12
f1 (ppm)

3a

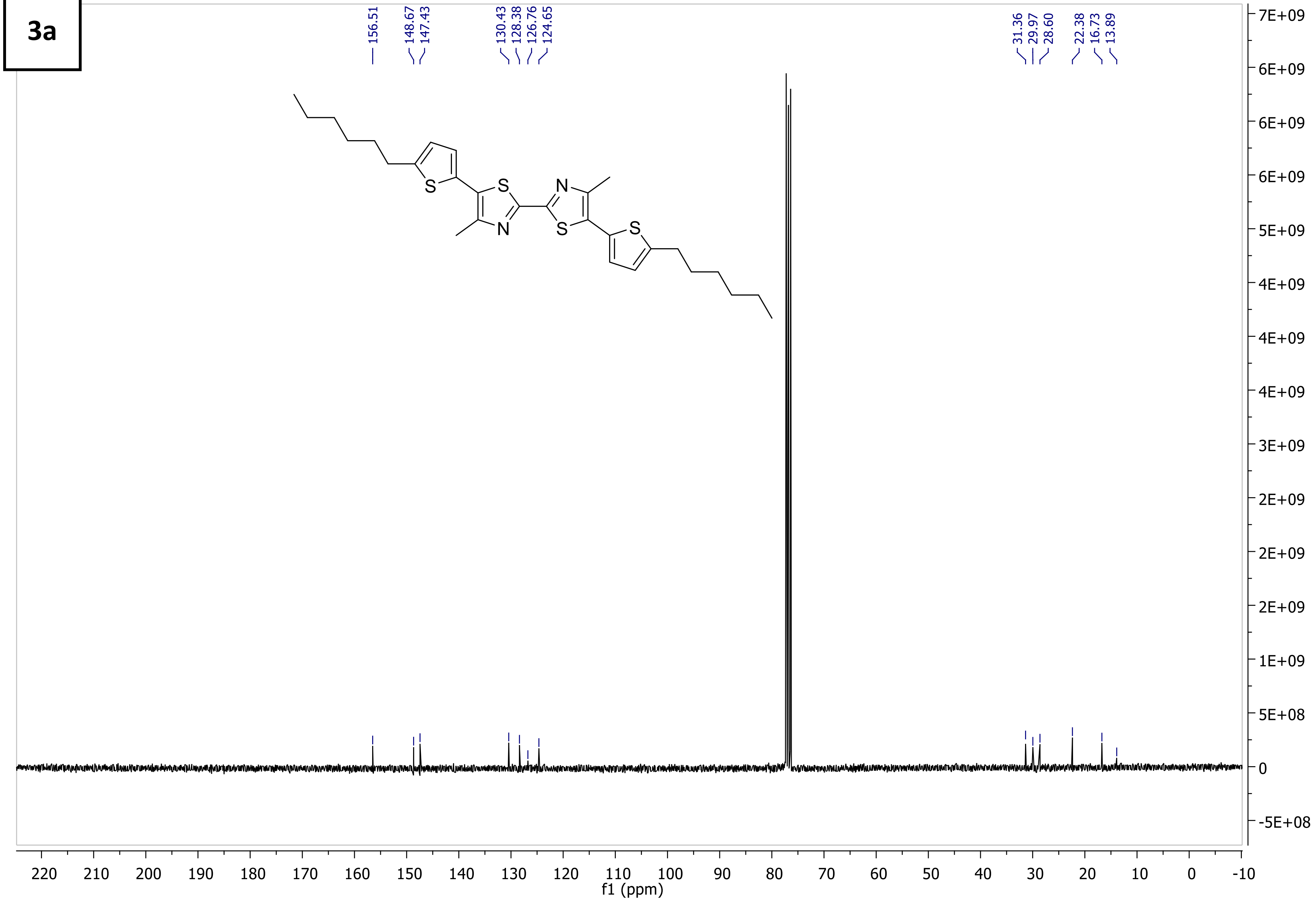

156.51
148.67
147.43
130.43
128.38
126.76
124.65
31.36
29.97
28.60
22.38
16.73
13.89
S
S
N
N
S
S
7E+09
6E+09
6E+09
6E+09
5E+09
4E+09
4E+09
4E+09
3E+09
2E+09
2E+09
2E+09
1E+09
5E+08
0
-5E+08
220
210
200
190
180
170
160
150
140
130
120
110
100
90
80
70
60
50
40
30
20
10
0
-10
f1 (ppm)

3b

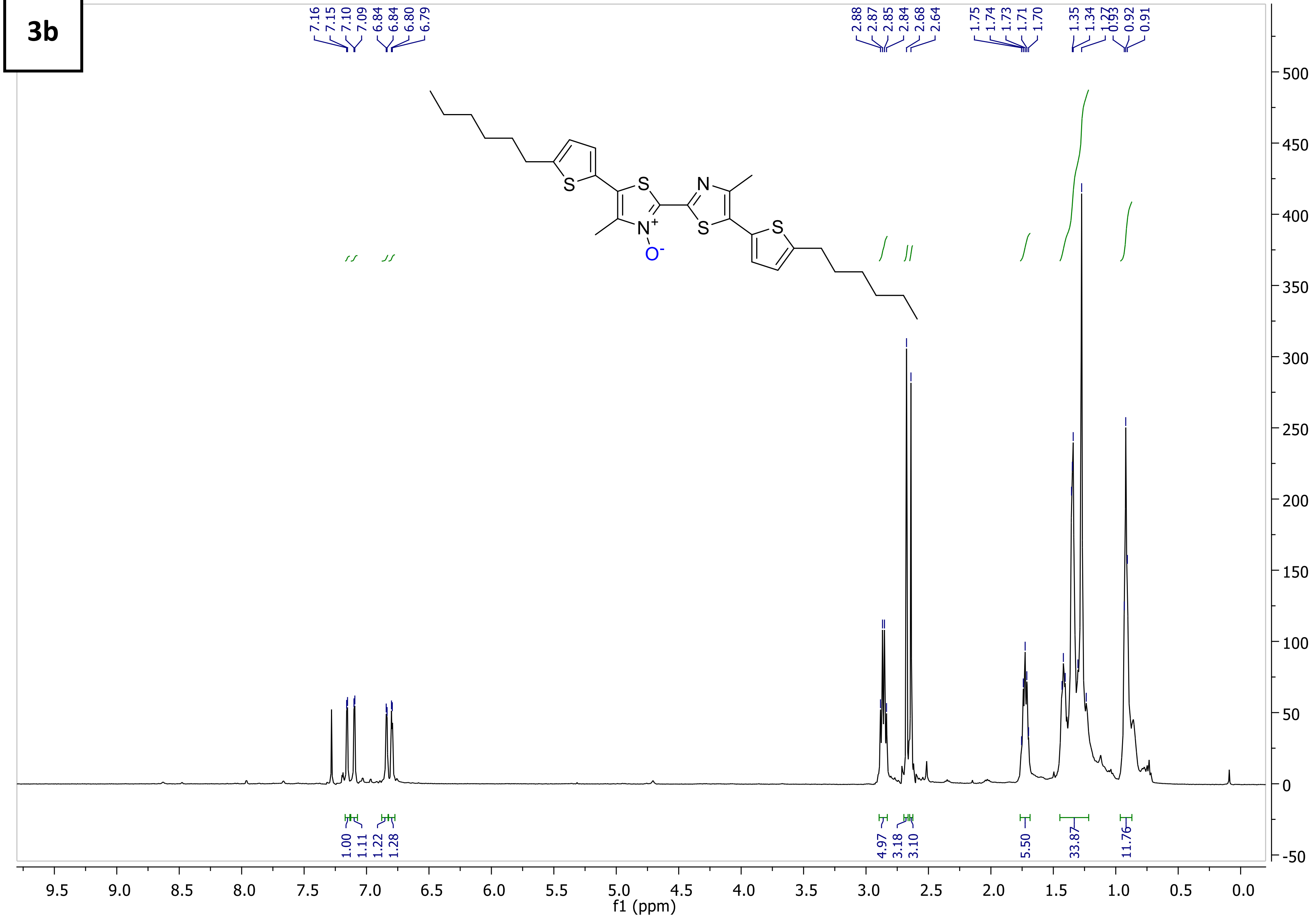

7.16
7.15
7.10
7.09
6.84
6.84
6.80
6.79
2.88
2.87
2.85
2.84
2.68
2.64
1.75
1.74
1.73
1.71
1.70
1.35
1.34
1.27
0.93
0.92
0.91
1.00
1.11
1.22
1.28
4.97
3.18
3.10
5.50
33.87
11.76
f1 (ppm)

3b

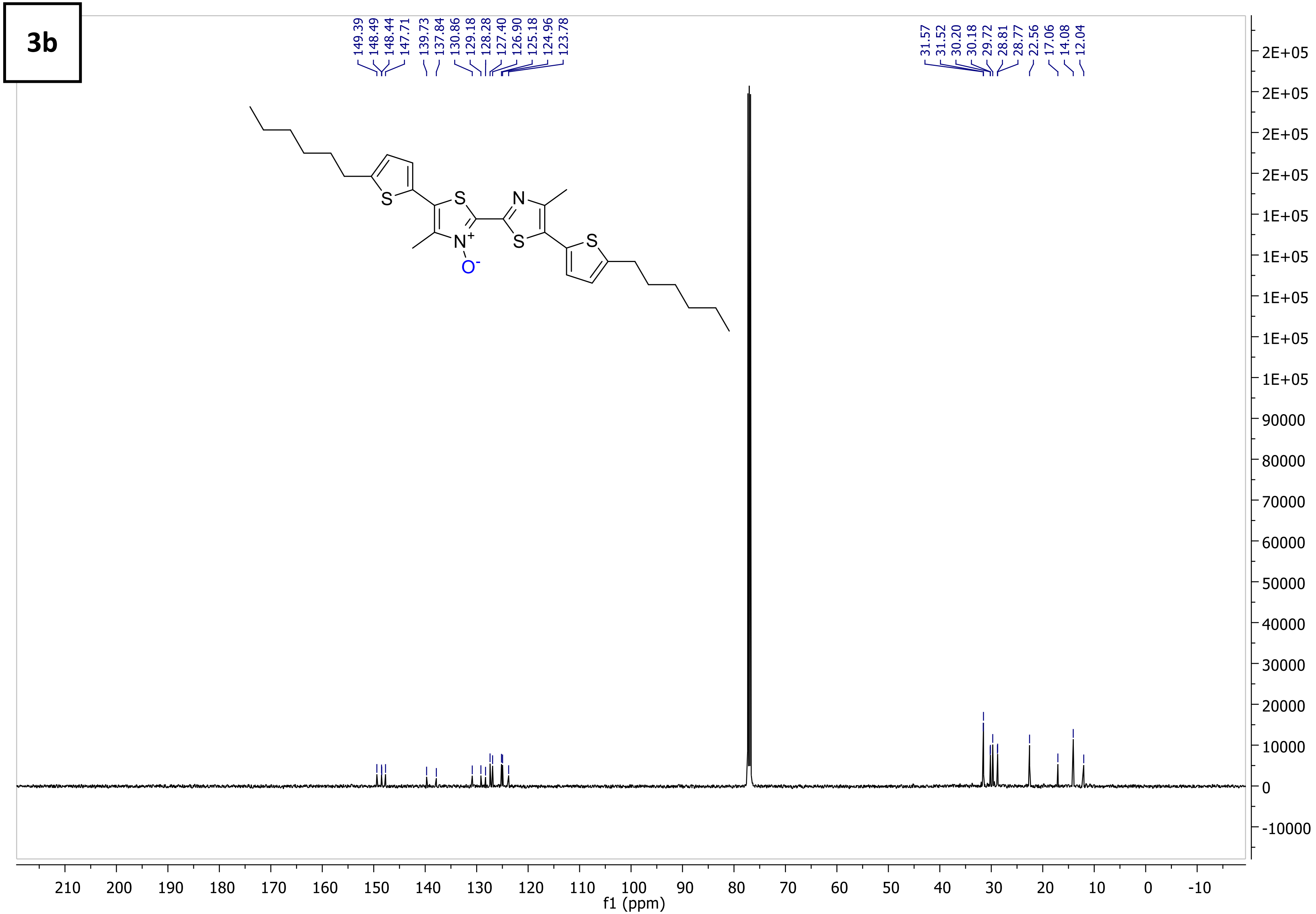
149.39
148.49
148.44
147.71
139.73
137.84
130.86
129.18
128.28
127.40
126.90
125.18
124.96
123.78
31.57
31.52
30.20
30.18
29.72
28.81
28.77
22.56
17.06
14.08
12.04
S
S
N+
O-
N
S
S
f1 (ppm)

3c

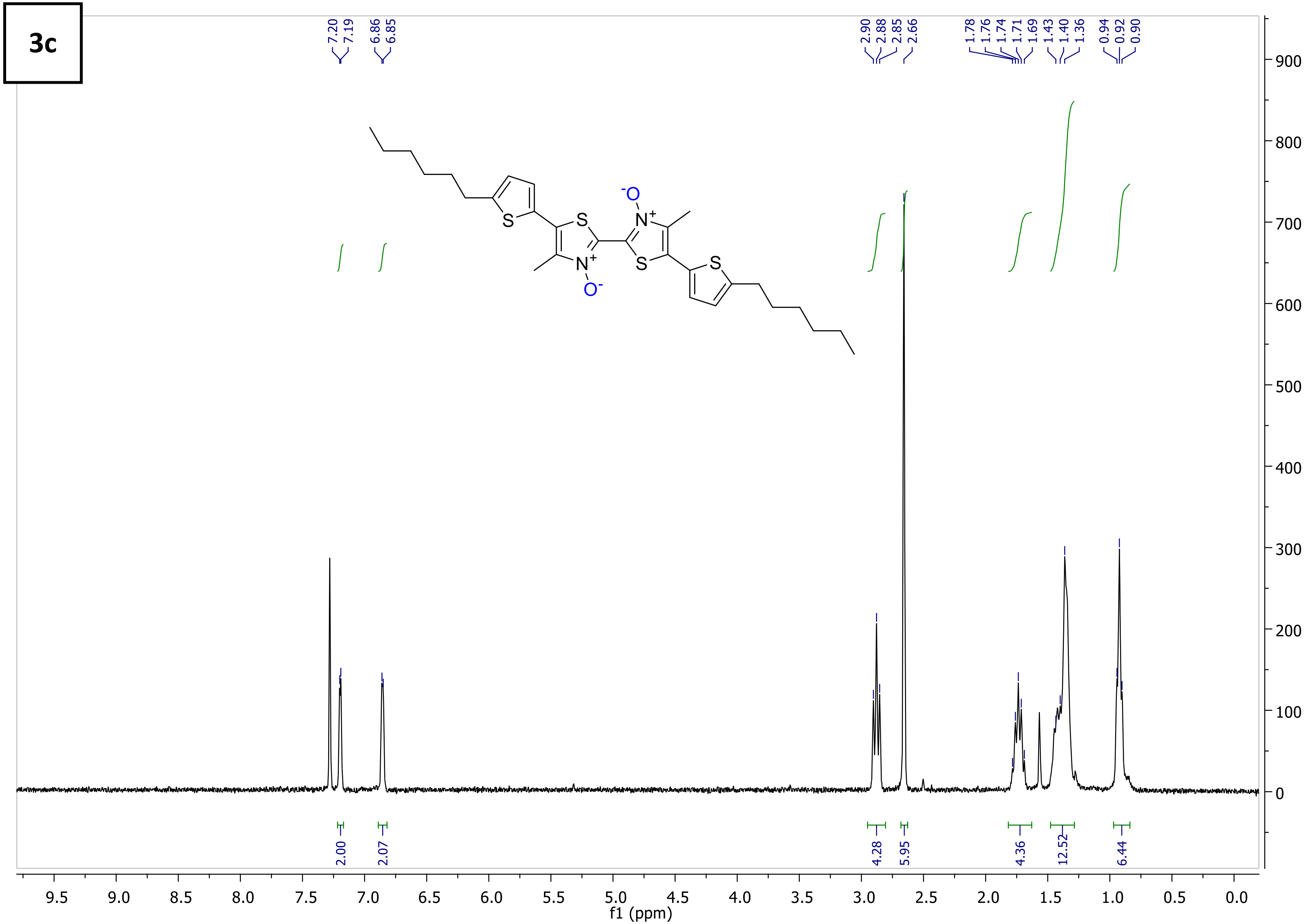
7.20
7.19
6.86
6.85
2.90
2.88
2.85
2.66
1.78
1.76
1.74
1.71
1.69
1.43
1.40
1.36
0.94
0.92
0.90
2.00
2.07
4.28
5.95
4.36
12.52
6.44
f1 (ppm)

3c

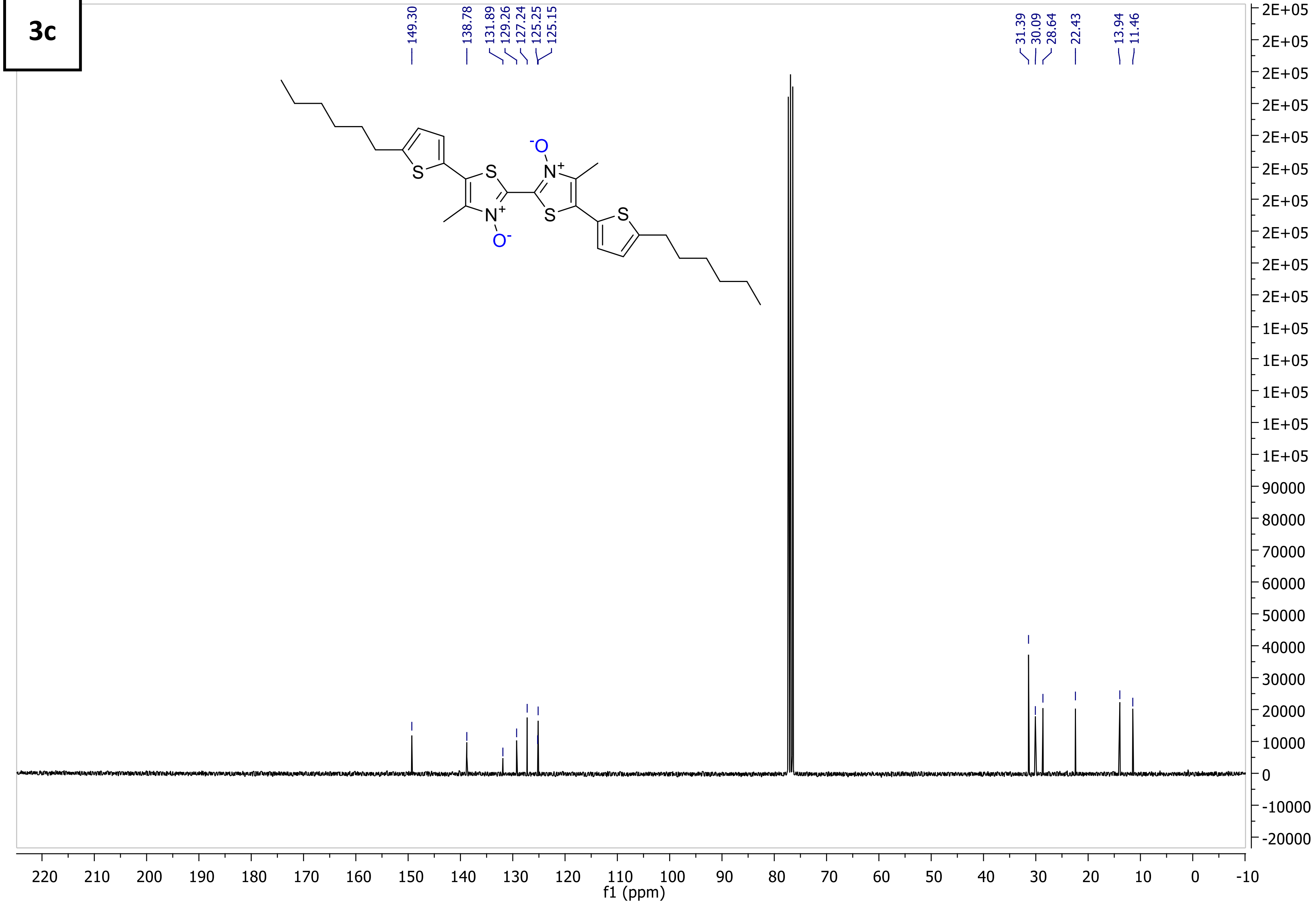
149.30
138.78
131.89
129.26
127.24
125.25
125.15
31.39
30.09
28.64
22.43
13.94
11.46
f1 (ppm)

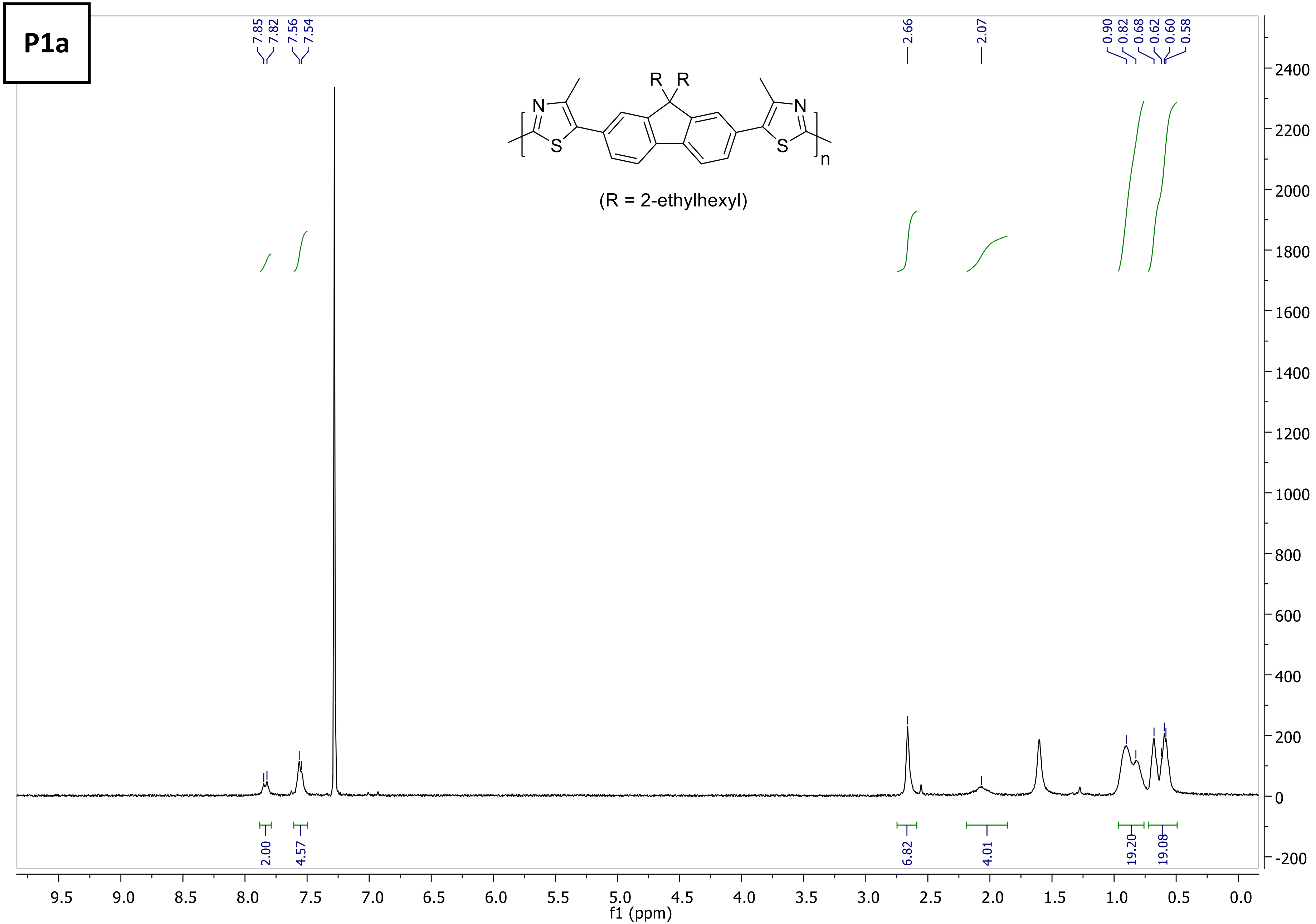

P1a
7.85
7.82
7.56
7.54
2.66
2.07
0.90
0.82
0.68
0.62
0.60
0.58
R R
N
S
n
(R = 2-ethylhexyl)
2.00
4.57
6.82
4.01
19.20
19.08
2400
2200
2000
1800
1600
1400
1200
1000
800
600
400
200
0
-200
9.5 9.0 8.5 8.0 7.5 7.0 6.5 6.0 5.5 5.0 4.5 4.0 3.5 3.0 2.5 2.0 1.5 1.0 0.5 0.0
f1 (ppm)

P1b

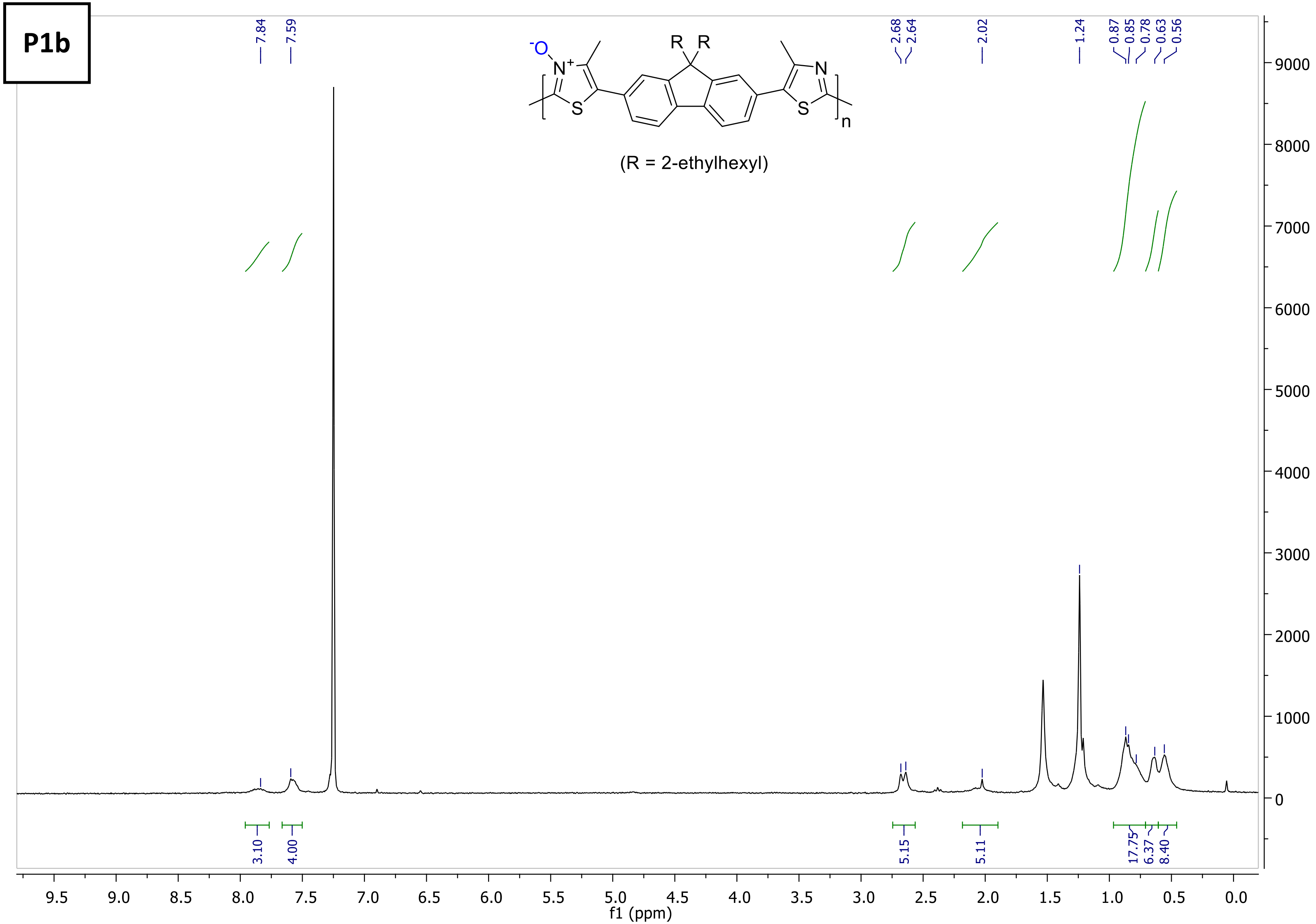
7.84
7.59
2.68
2.64
2.02
1.24
0.87
0.85
0.78
0.63
0.56
(R = 2-ethylhexyl)
3.10
4.00
5.15
5.11
17.75
6.37
8.40
f1 (ppm)

P1c

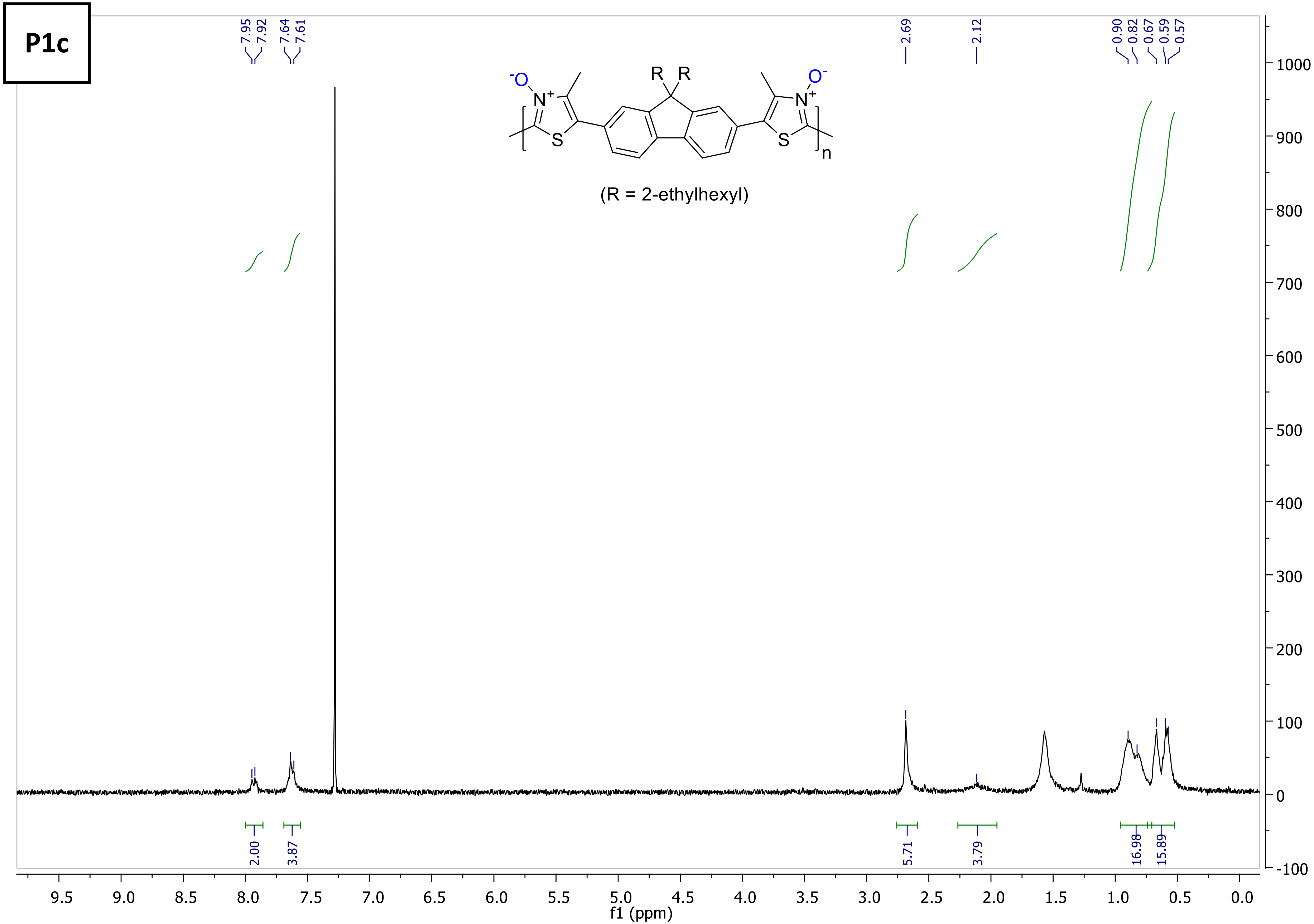

7.95
7.92
7.64
7.61
2.69
2.12
0.90
0.82
0.67
0.59
0.57
(R = 2-ethylhexyl)
2.00
3.87
5.71
3.79
16.98
15.89
f1 (ppm)

P2a

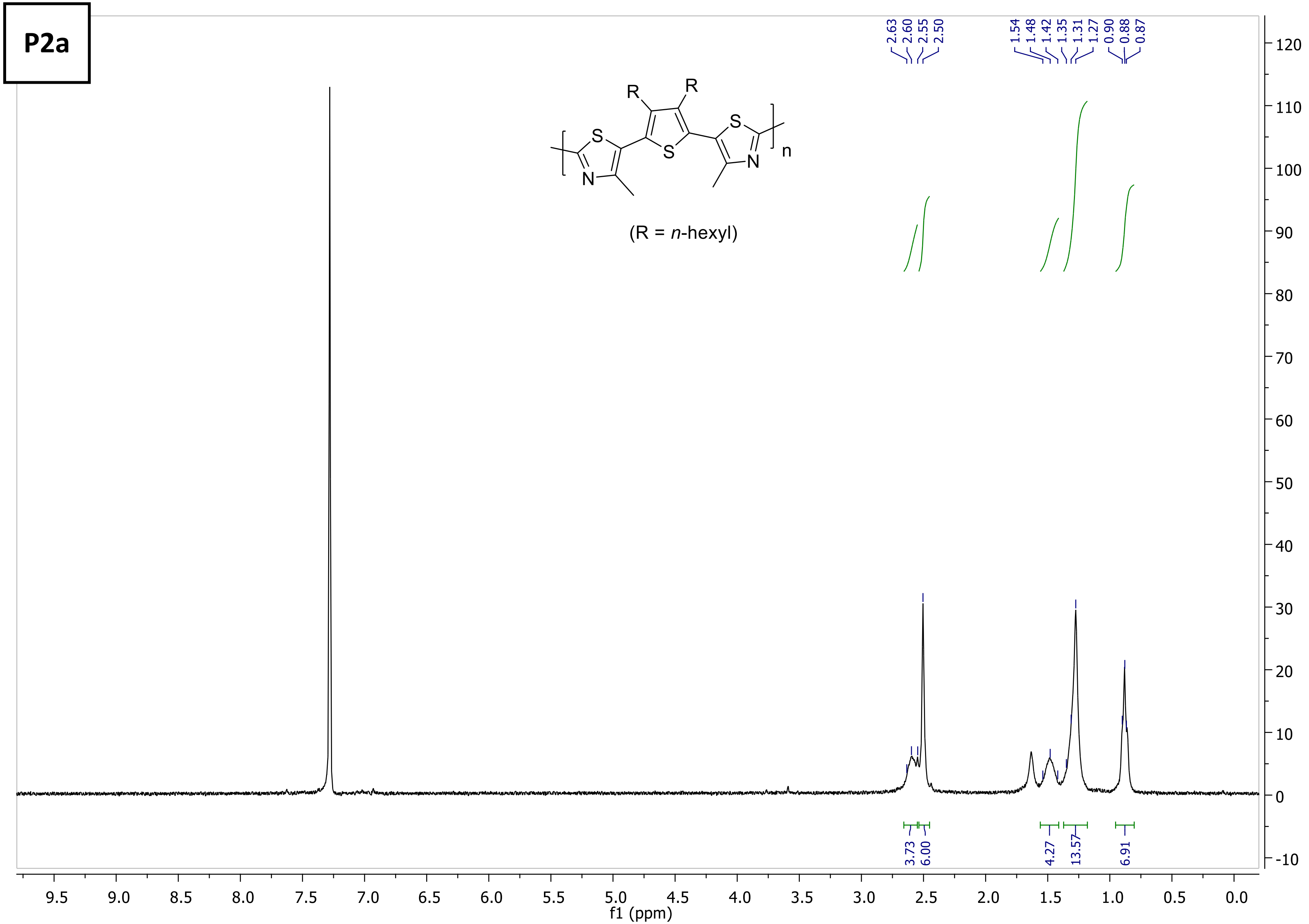
2.63
2.60
2.55
2.50
1.54
1.48
1.42
1.35
1.31
1.27
0.90
0.88
0.87
R
S
N
n
(R = n-hexyl)
3.73
6.00
4.27
13.57
6.91
9.5 9.0 8.5 8.0 7.5 7.0 6.5 6.0 5.5 5.0 4.5 4.0 3.5 3.0 2.5 2.0 1.5 1.0 0.5 0.0
f1 (ppm)
120 110 100 90 80 70 60 50 40 30 20 10 0 -10

P2b

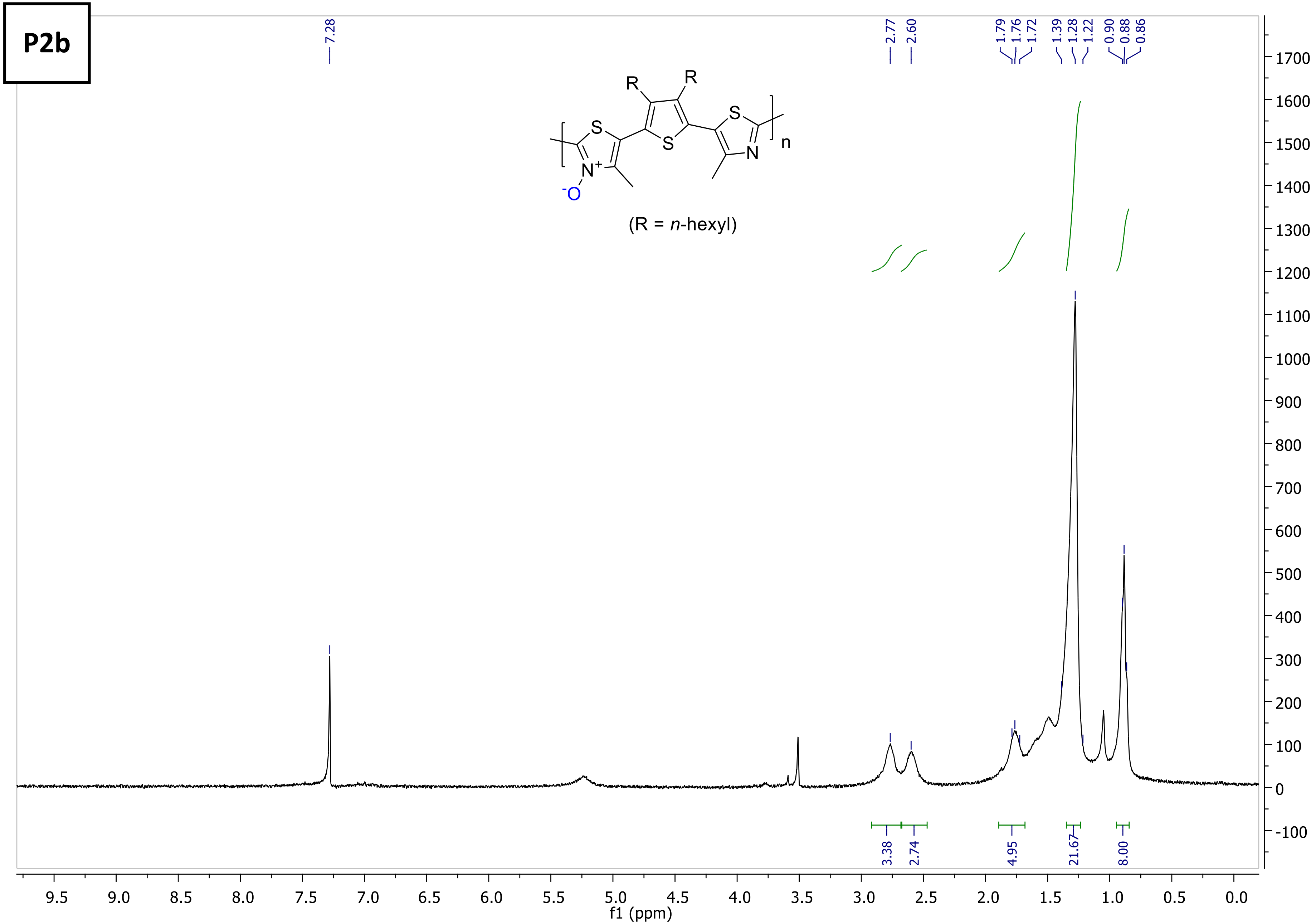
7.28
2.77
2.60
1.79
1.76
1.72
1.39
1.28
1.22
0.90
0.88
0.86
(R = n-hexyl)
3.38
2.74
4.95
21.67
8.00
f1 (ppm)

P2c

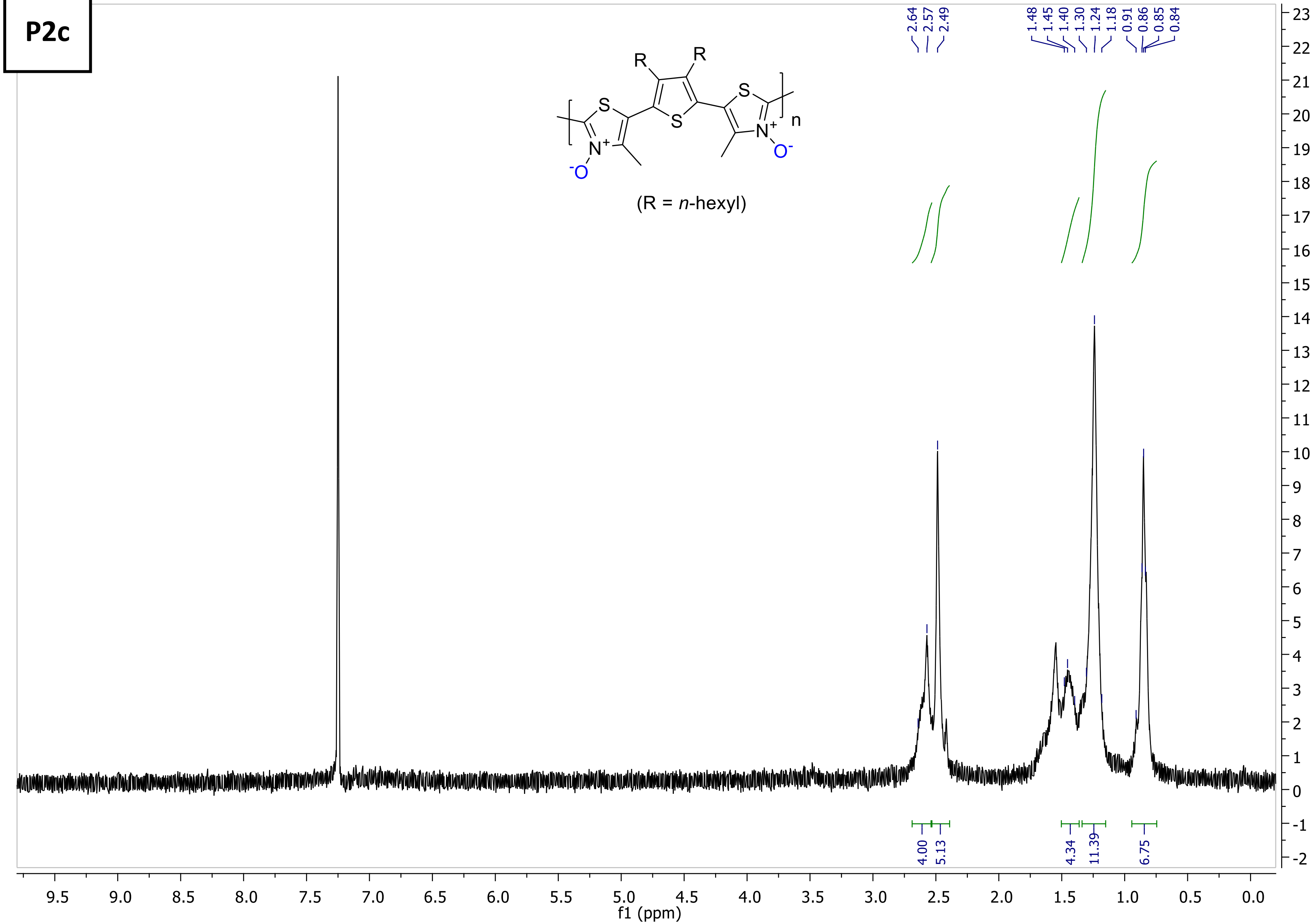

(R = n-hexyl)
2.64
2.57
2.49
1.48
1.45
1.40
1.30
1.24
1.18
0.91
0.86
0.85
0.84
4.00
5.13
4.34
11.39
6.75
f1 (ppm)

S1

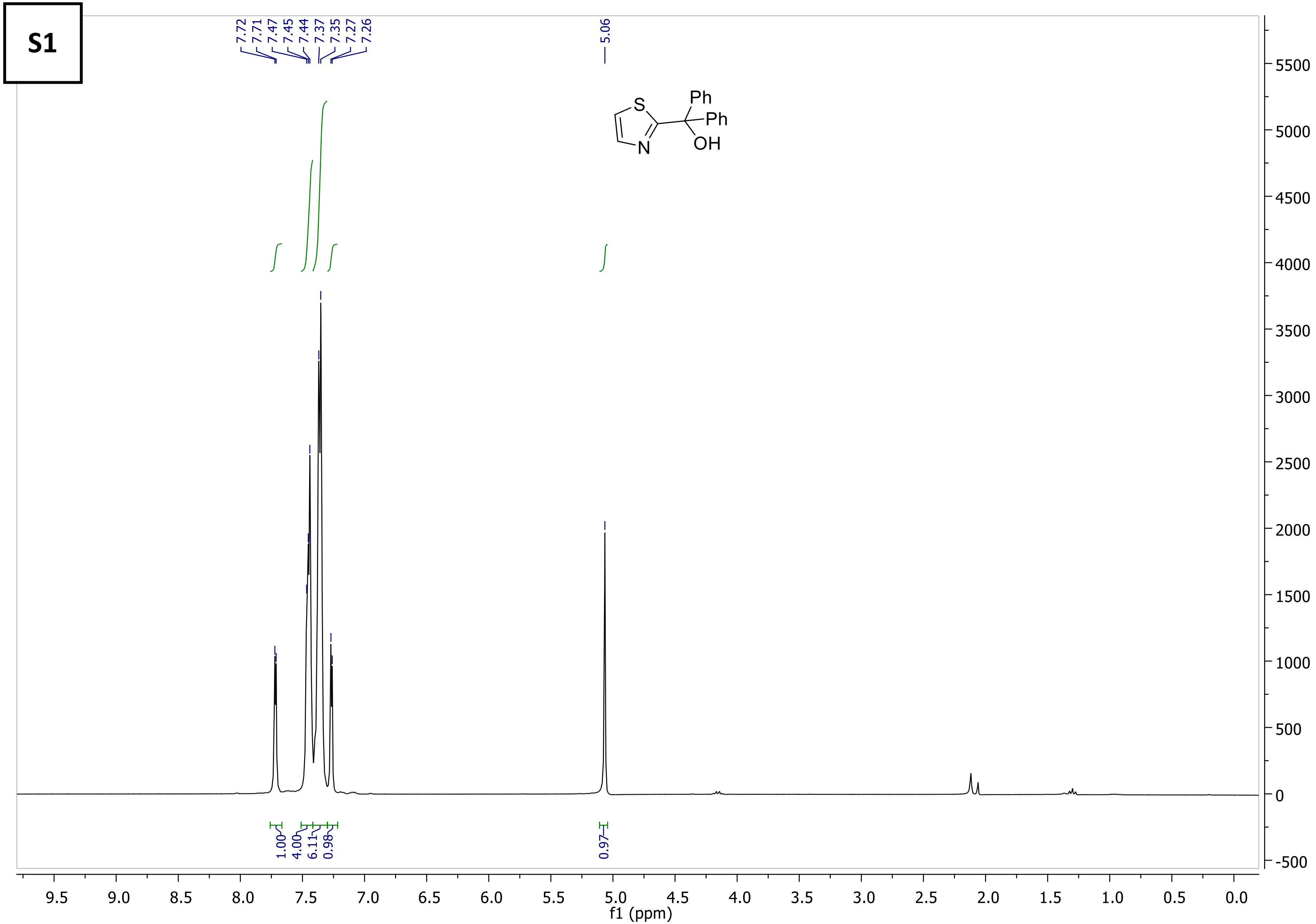
7.72
7.71
7.47
7.45
7.44
7.37
7.35
7.27
7.26
5.06
S
N
Ph
Ph
OH
1.00
4.00
6.11
0.98
0.97
f1 (ppm)

S1

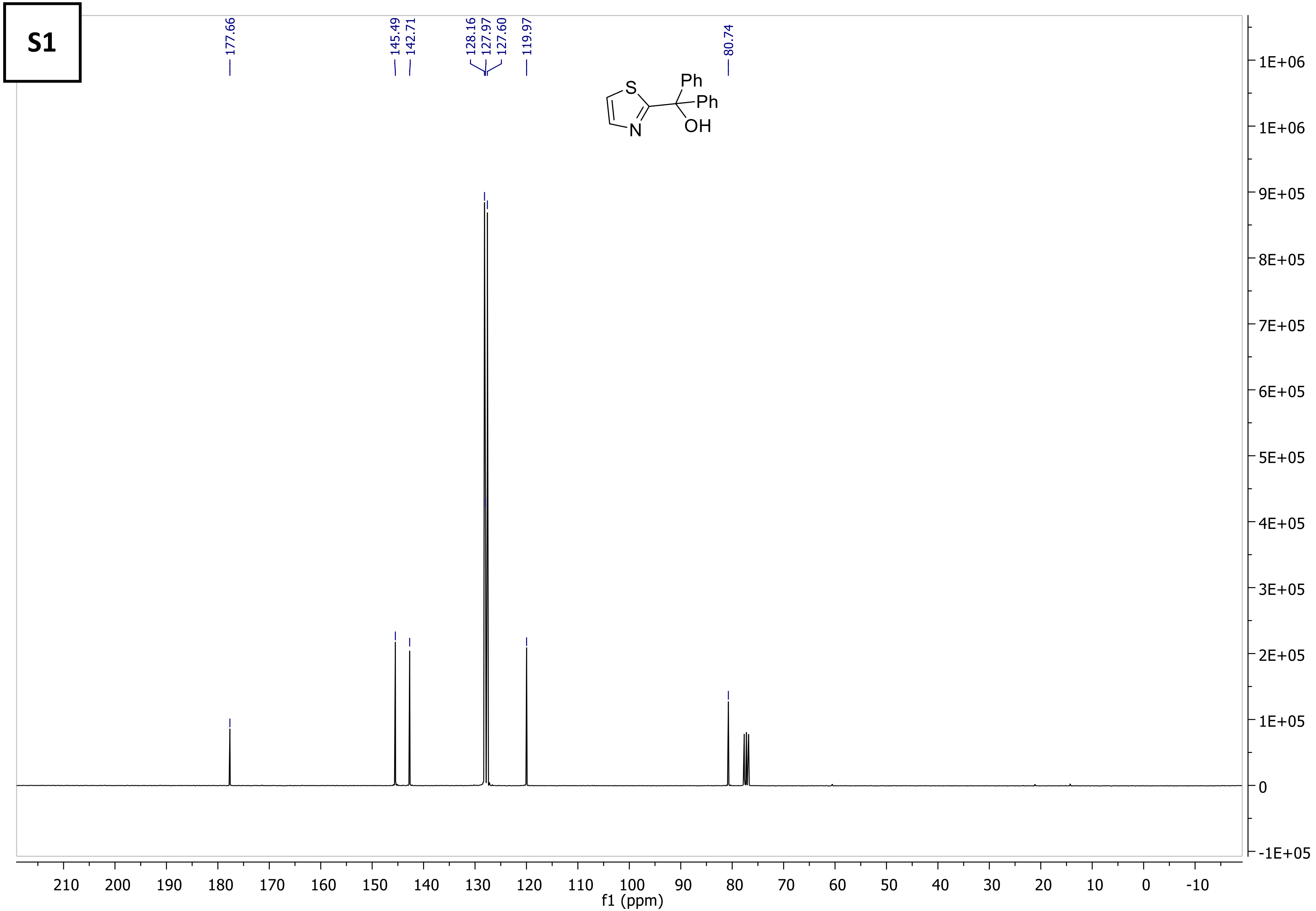
177.66
145.49
142.71
128.16
127.97
127.60
119.97
80.74
S
N
Ph
Ph
OH
1E+06
1E+06
9E+05
8E+05
7E+05
6E+05
5E+05
4E+05
3E+05
2E+05
1E+05
0
-1E+05
210 200 190 180 170 160 150 140 130 120 110 100 90 80 70 60 50 40 30 20 10 0 -10
f1 (ppm)

S2

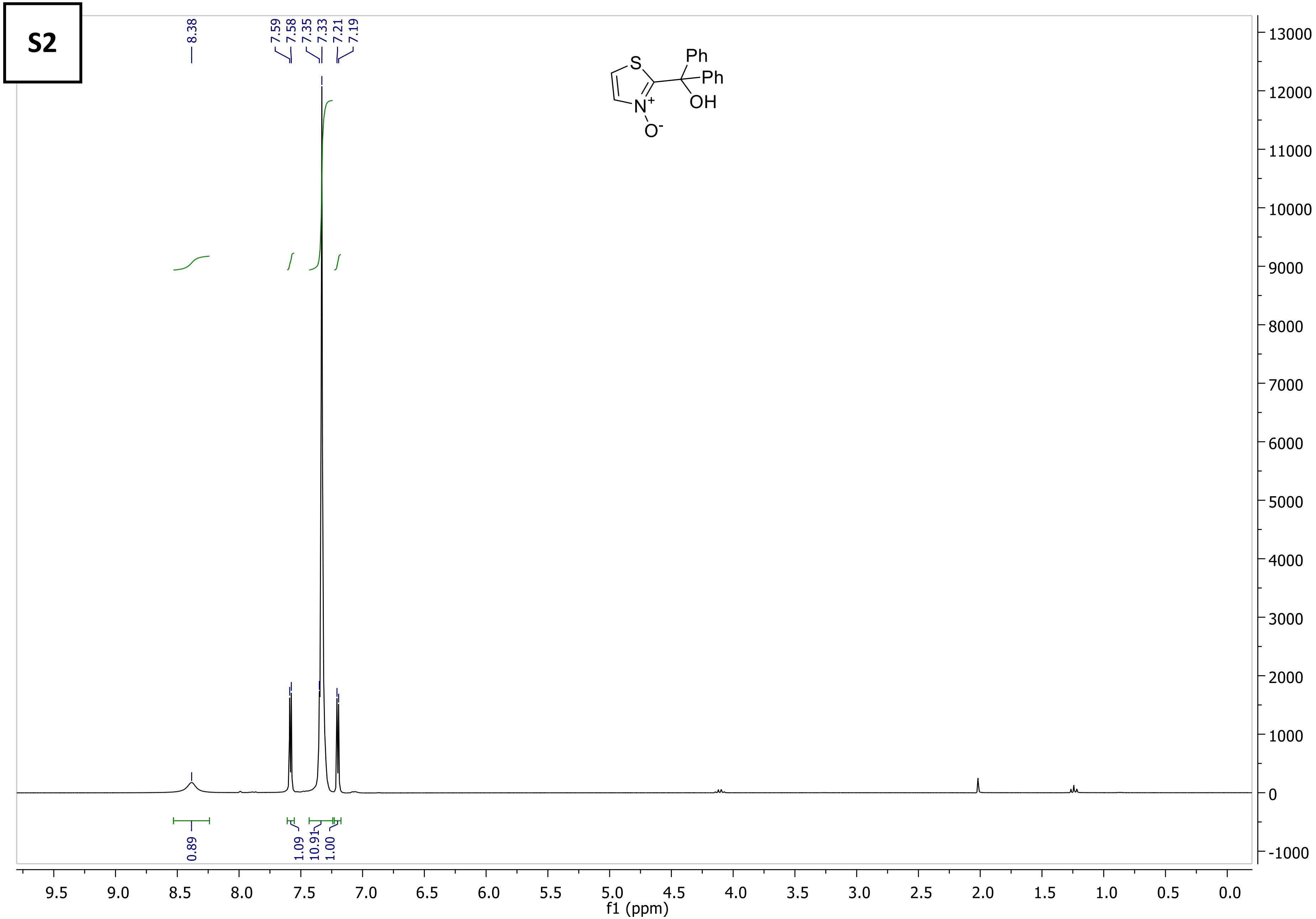

8.38
7.59
7.58
7.35
7.33
7.21
7.19
S
Ph
Ph
N+
OH
O-
0.89
1.09
10.91
1.00
13000
12000
11000
10000
9000
8000
7000
6000
5000
4000
3000
2000
1000
0
-1000
9.5
9.0
8.5
8.0
7.5
7.0
6.5
6.0
5.5
5.0
4.5
4.0
3.5
3.0
2.5
2.0
1.5
1.0
0.5
0.0
f1 (ppm)

S2

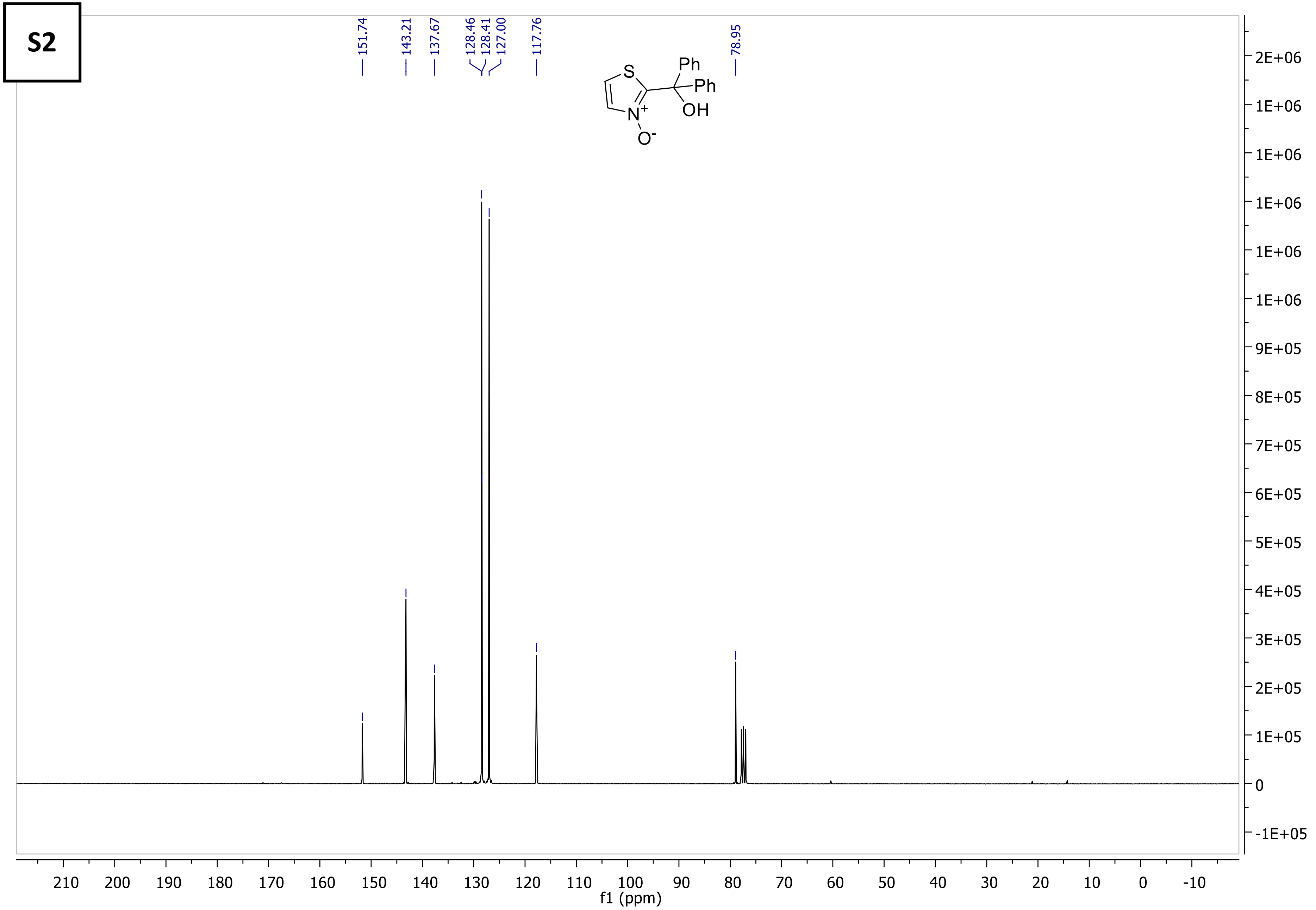
151.74
143.21
137.67
128.46
128.41
127.00
117.76
78.95
S
N+
O-
Ph
Ph
OH
f1 (ppm)

S3

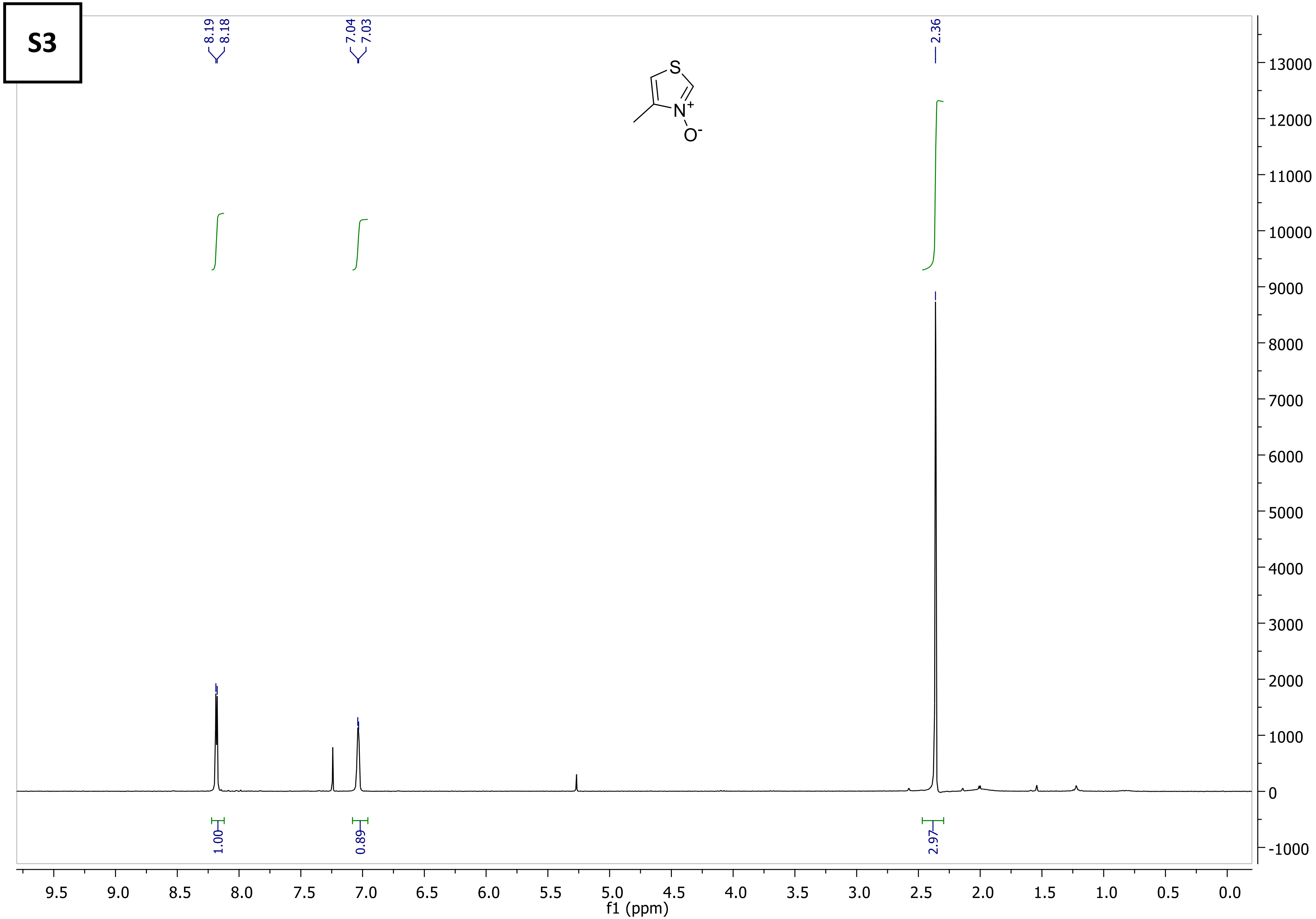
8.19
8.18
7.04
7.03
2.36
S
N+
O-
1.00
0.89
2.97
9.5 9.0 8.5 8.0 7.5 7.0 6.5 6.0 5.5 5.0 4.5 4.0 3.5 3.0 2.5 2.0 1.5 1.0 0.5 0.0
f1 (ppm)
13000 12000 11000 10000 9000 8000 7000 6000 5000 4000 3000 2000 1000 0 -1000

S3

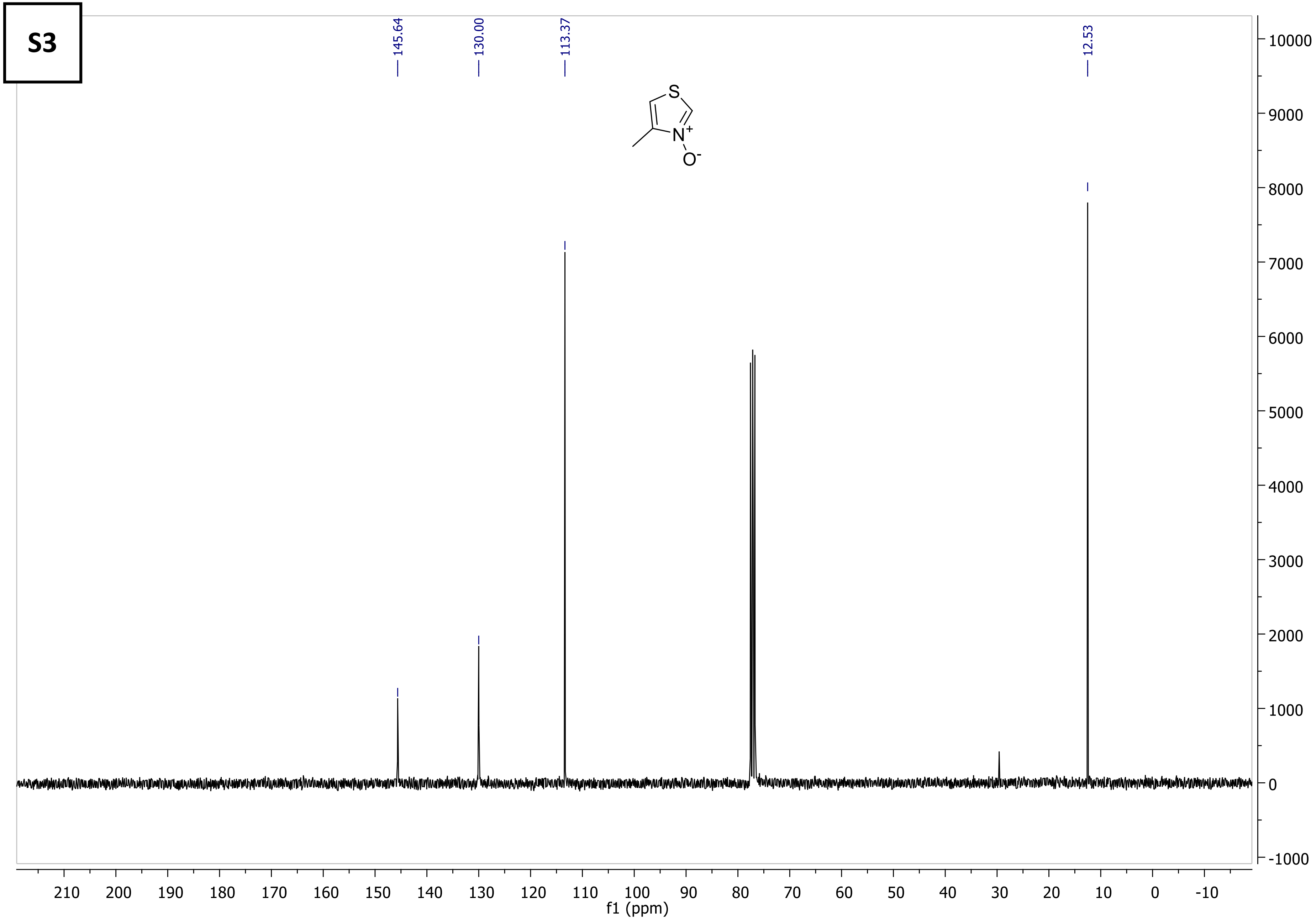

145.64
130.00
113.37
12.53
S
N+
O-
10000
9000
8000
7000
6000
5000
4000
3000
2000
1000
0
-1000
210
200
190
180
170
160
150
140
130
120
110
100
90
80
70
60
50
40
30
20
10
0
-10
f1 (ppm)

S4

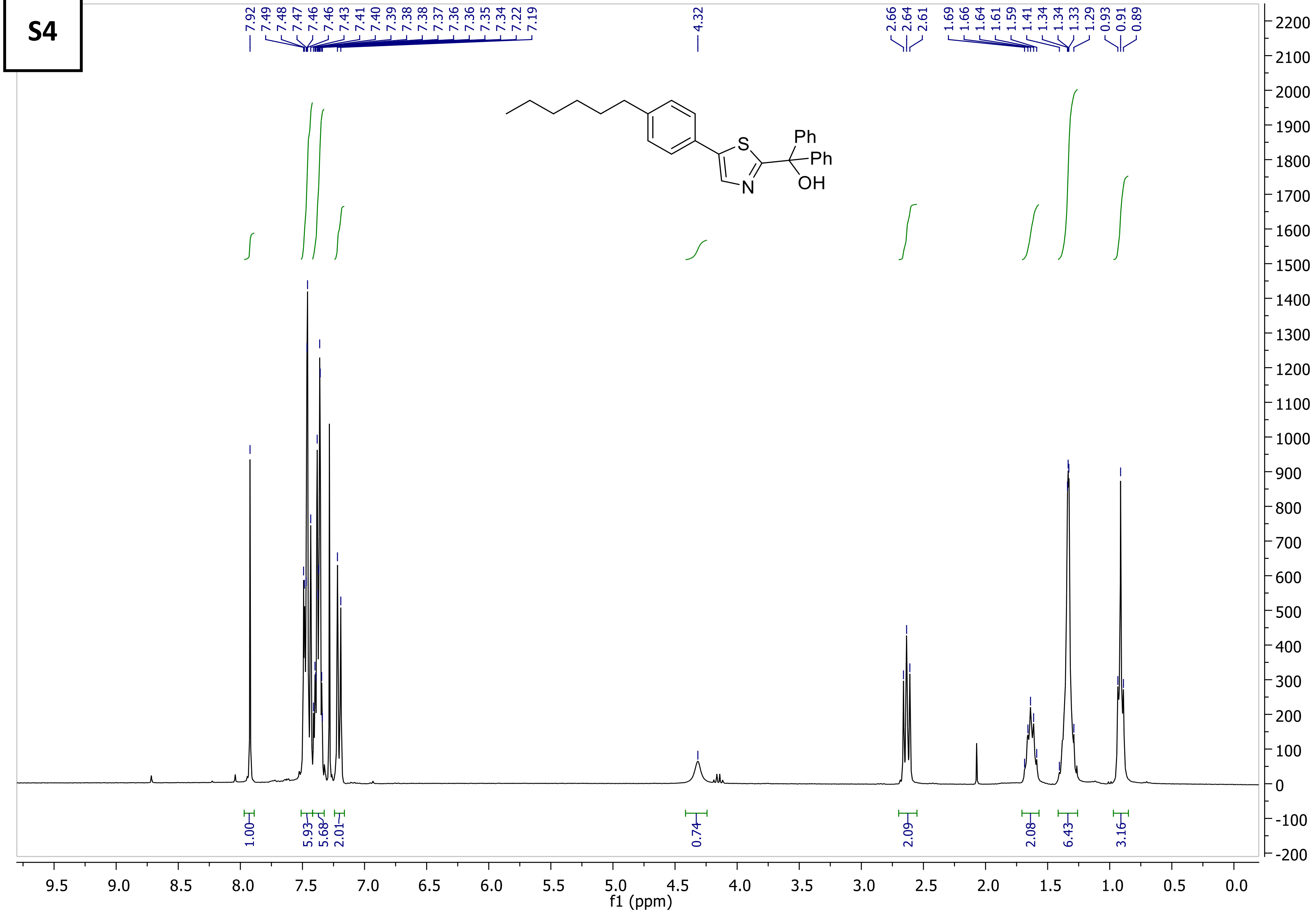

7.92
7.49
7.48
7.47
7.46
7.46
7.43
7.41
7.40
7.39
7.38
7.38
7.37
7.36
7.36
7.35
7.34
7.22
7.19
4.32
2.66
2.64
2.61
1.69
1.66
1.64
1.61
1.59
1.41
1.34
1.33
1.29
0.93
0.91
0.89
S
N
Ph
Ph
OH
1.00
5.93
5.68
2.01
0.74
2.09
2.08
6.43
3.16
f1 (ppm)

S4

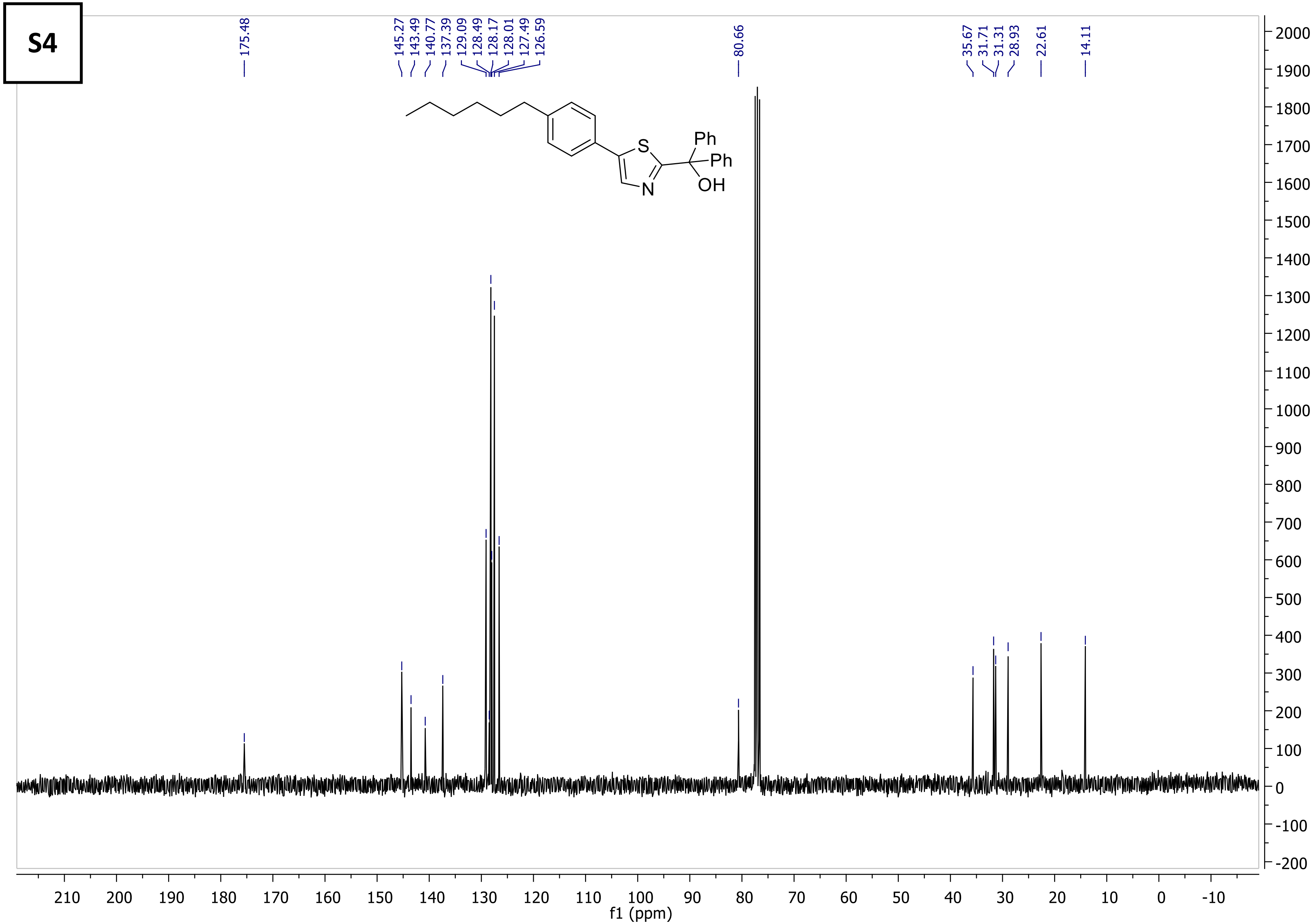

175.48
145.27
143.49
140.77
137.39
129.09
128.49
128.17
128.01
127.49
126.59
80.66
35.67
31.71
31.31
28.93
22.61
14.11
S
N
Ph
Ph
OH
f1 (ppm)

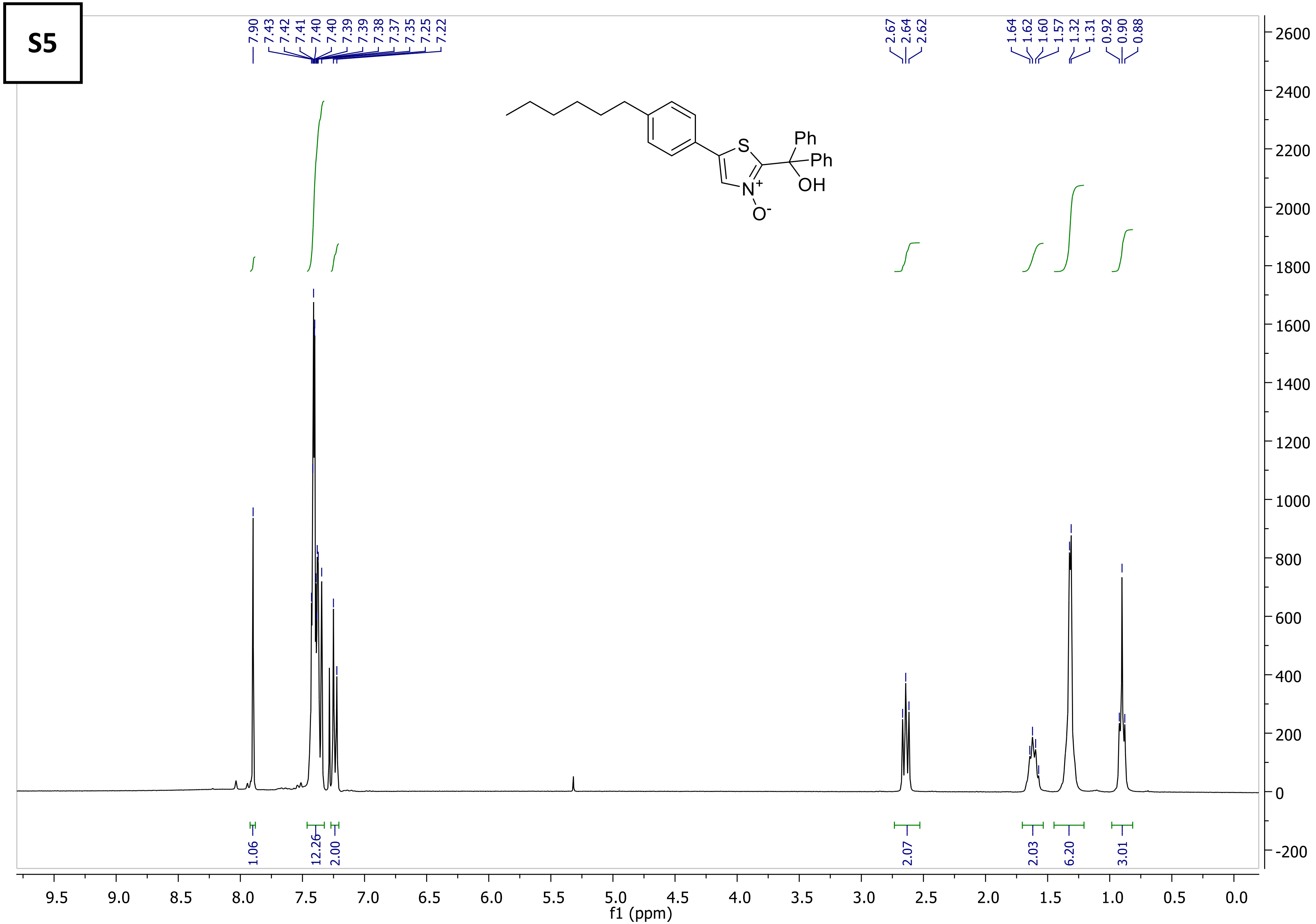
S5
7.90
7.43
7.42
7.41
7.40
7.40
7.39
7.39
7.38
7.37
7.35
7.25
7.22
2.67
2.64
2.62
1.64
1.62
1.60
1.57
1.32
1.31
0.92
0.90
0.88
S
N+
O-
Ph
Ph
OH
1.06
12.26
2.00
2.07
2.03
6.20
3.01
f1 (ppm)

S5

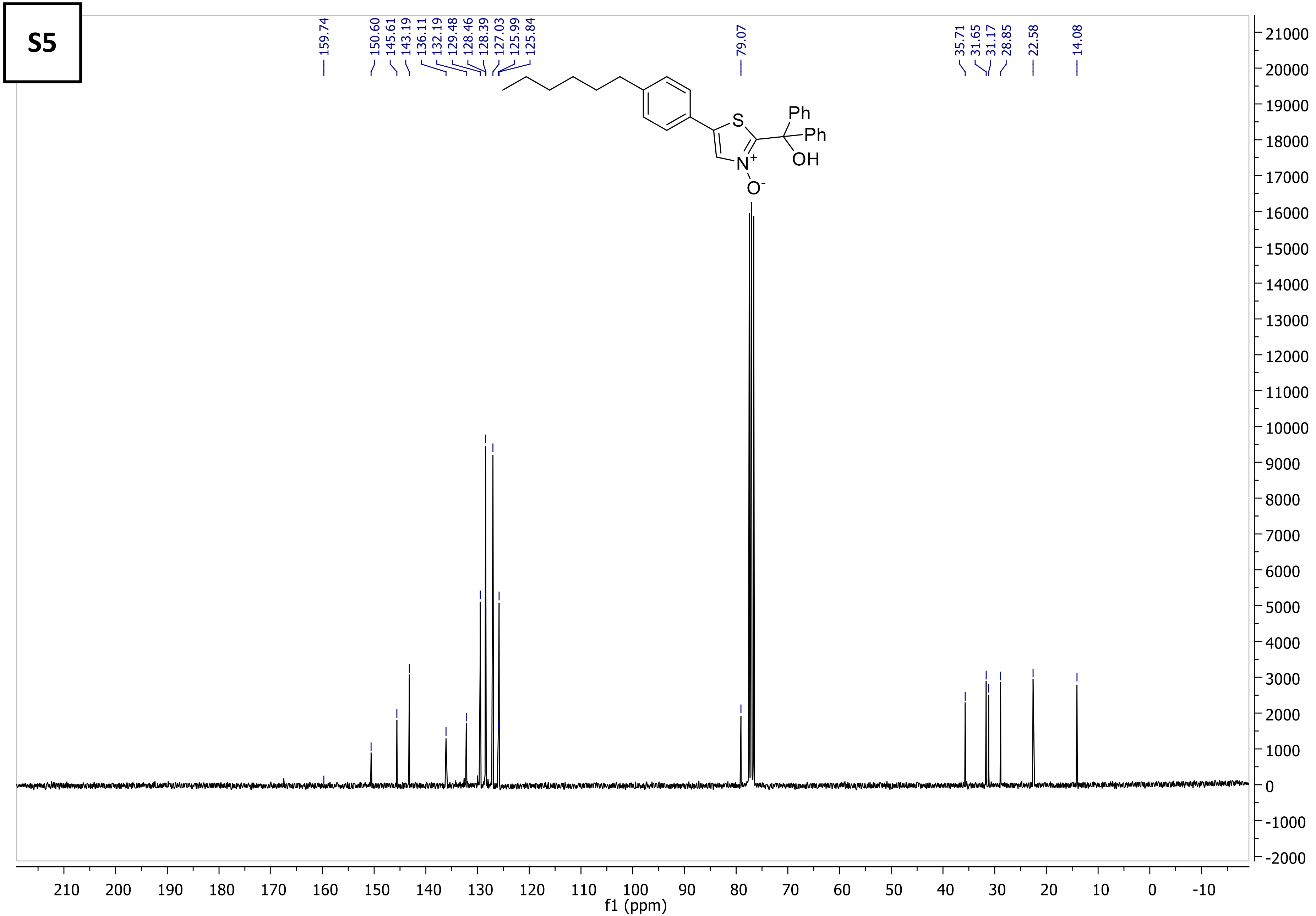
159.74
150.60
145.61
143.19
136.11
132.19
129.48
128.46
128.39
127.03
125.99
125.84
79.07
35.71
31.65
31.17
28.85
22.58
14.08
S
N+
O-
Ph
Ph
OH
210 200 190 180 170 160 150 140 130 120 110 100 90 80 70 60 50 40 30 20 10 0 -10
f1 (ppm)
21000 20000 19000 18000 17000 16000 15000 14000 13000 12000 11000 10000 9000 8000 7000 6000 5000 4000 3000 2000 1000 0 -1000 -2000

2b

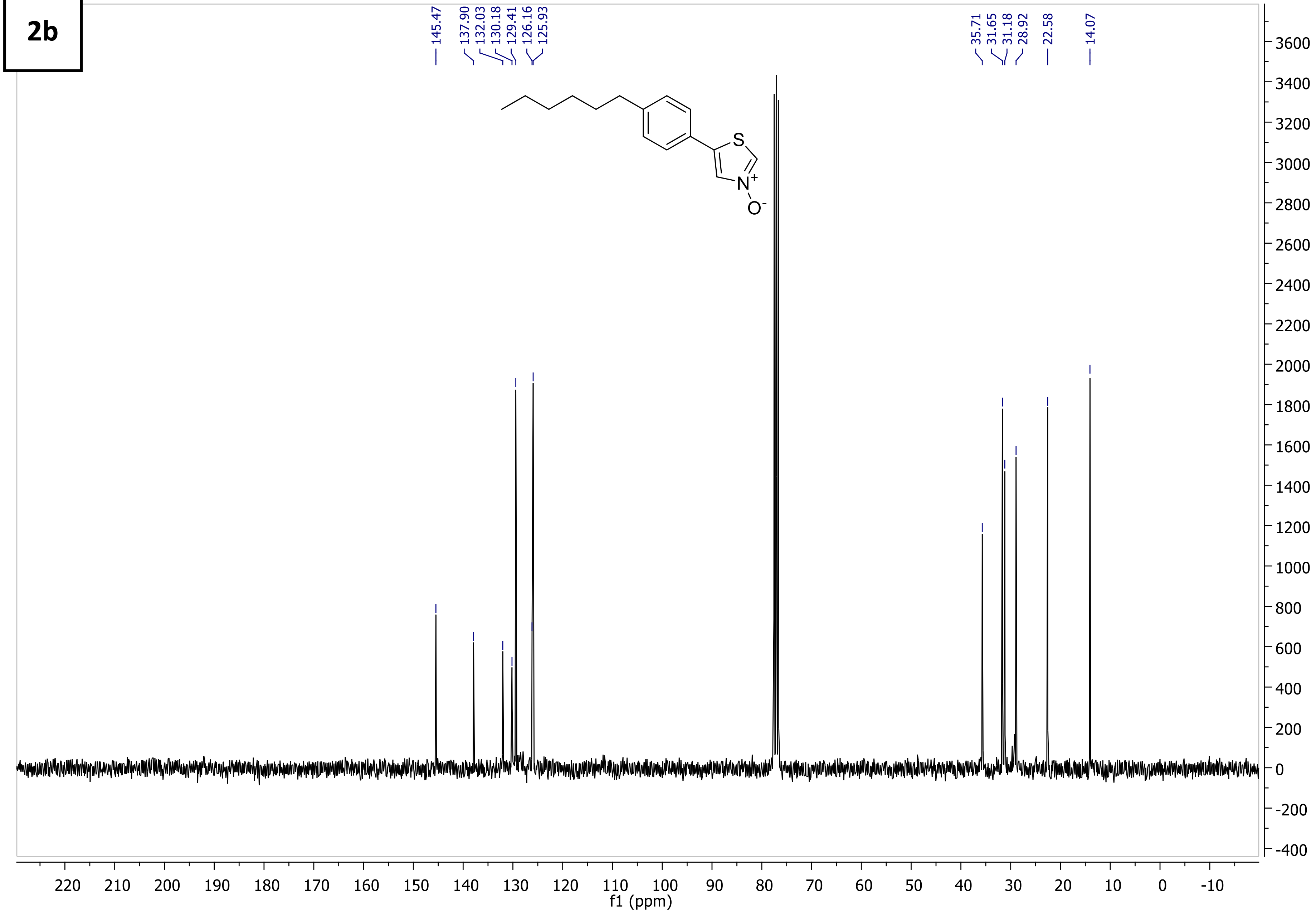

145.47
137.90
132.03
130.18
129.41
126.16
125.93
35.71
31.65
31.18
28.92
22.58
14.07
S
N+
O-
3600
3400
3200
3000
2800
2600
2400
2200
2000
1800
1600
1400
1200
1000
800
600
400
200
0
-200
-400
220 210 200 190 180 170 160 150 140 130 120 110 100 90 80 70 60 50 40 30 20 10 0 -10
f1 (ppm)

S6

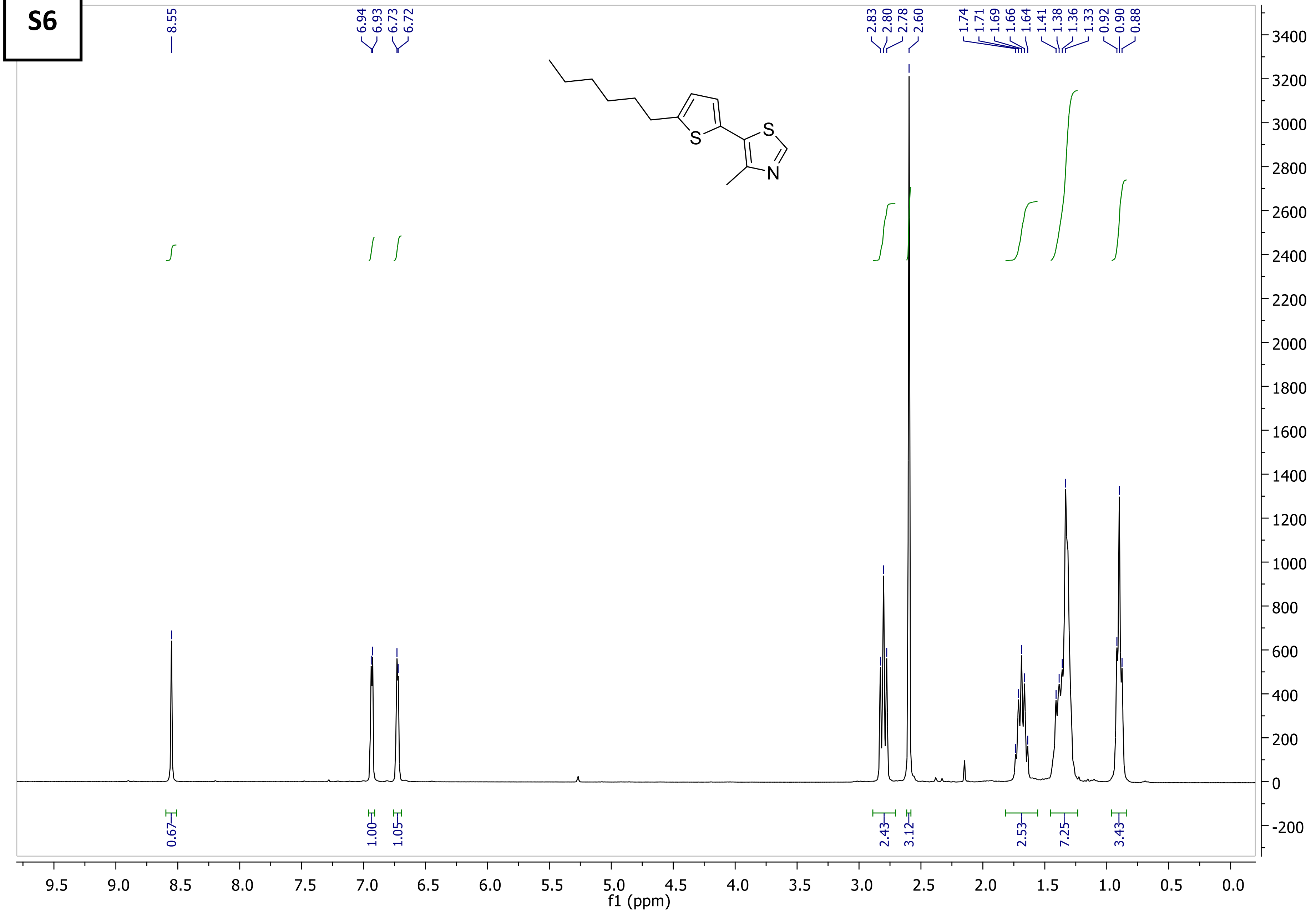

8.55
6.94
6.93
6.73
6.72
2.83
2.80
2.78
2.60
1.74
1.71
1.69
1.66
1.64
1.41
1.38
1.36
1.33
0.92
0.90
0.88
S
S
N
0.67
1.00
1.05
2.43
3.12
2.53
7.25
3.43
9.5 9.0 8.5 8.0 7.5 7.0 6.5 6.0 5.5 5.0 4.5 4.0 3.5 3.0 2.5 2.0 1.5 1.0 0.5 0.0
f1 (ppm)
3400 3200 3000 2800 2600 2400 2200 2000 1800 1600 1400 1200 1000 800 600 400 200 0 -200

S6

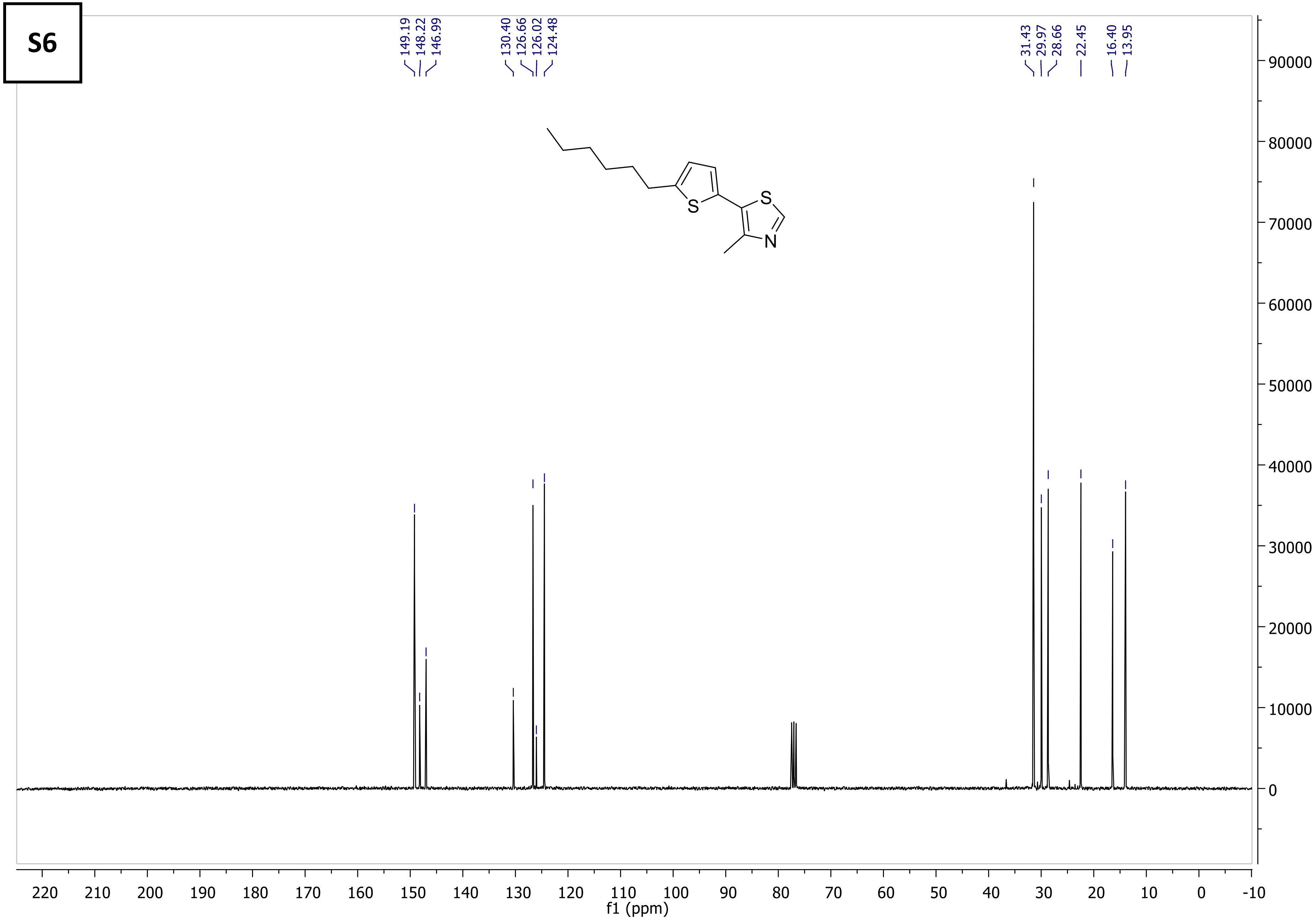
149.19
148.22
146.99
130.40
126.66
126.02
124.48
31.43
29.97
28.66
22.45
16.40
13.95
S
S
N
90000
80000
70000
60000
50000
40000
30000
20000
10000
0
220 210 200 190 180 170 160 150 140 130 120 110 100 90 80 70 60 50 40 30 20 10 0 -10
f1 (ppm)

S7

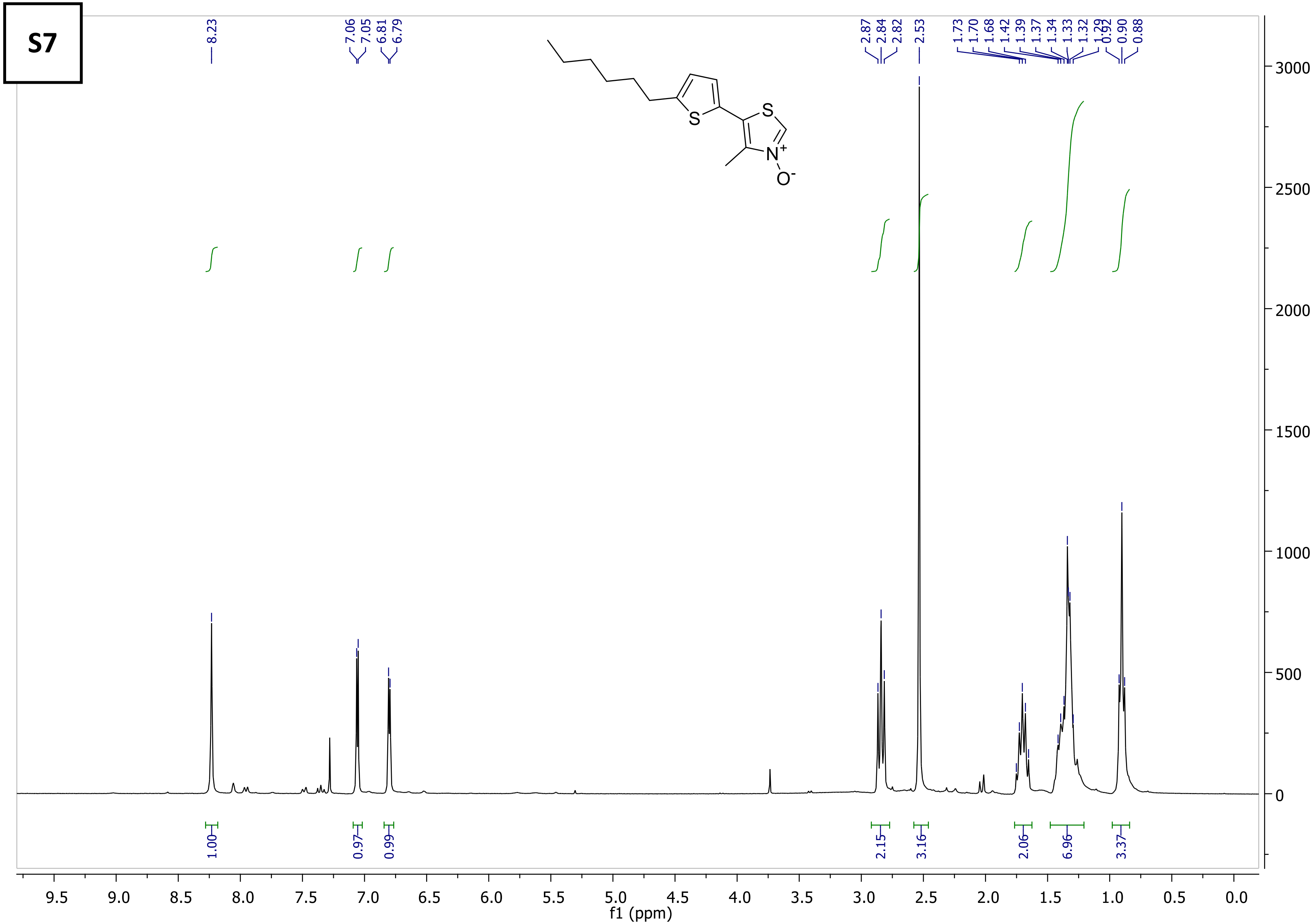
8.23
7.06
7.05
6.81
6.79
2.87
2.84
2.82
2.53
1.73
1.70
1.68
1.42
1.39
1.37
1.34
1.33
1.32
1.29
0.92
0.90
0.88
S
S
N+
O-
1.00
0.97
0.99
2.15
3.16
2.06
6.96
3.37
f1 (ppm)

S8

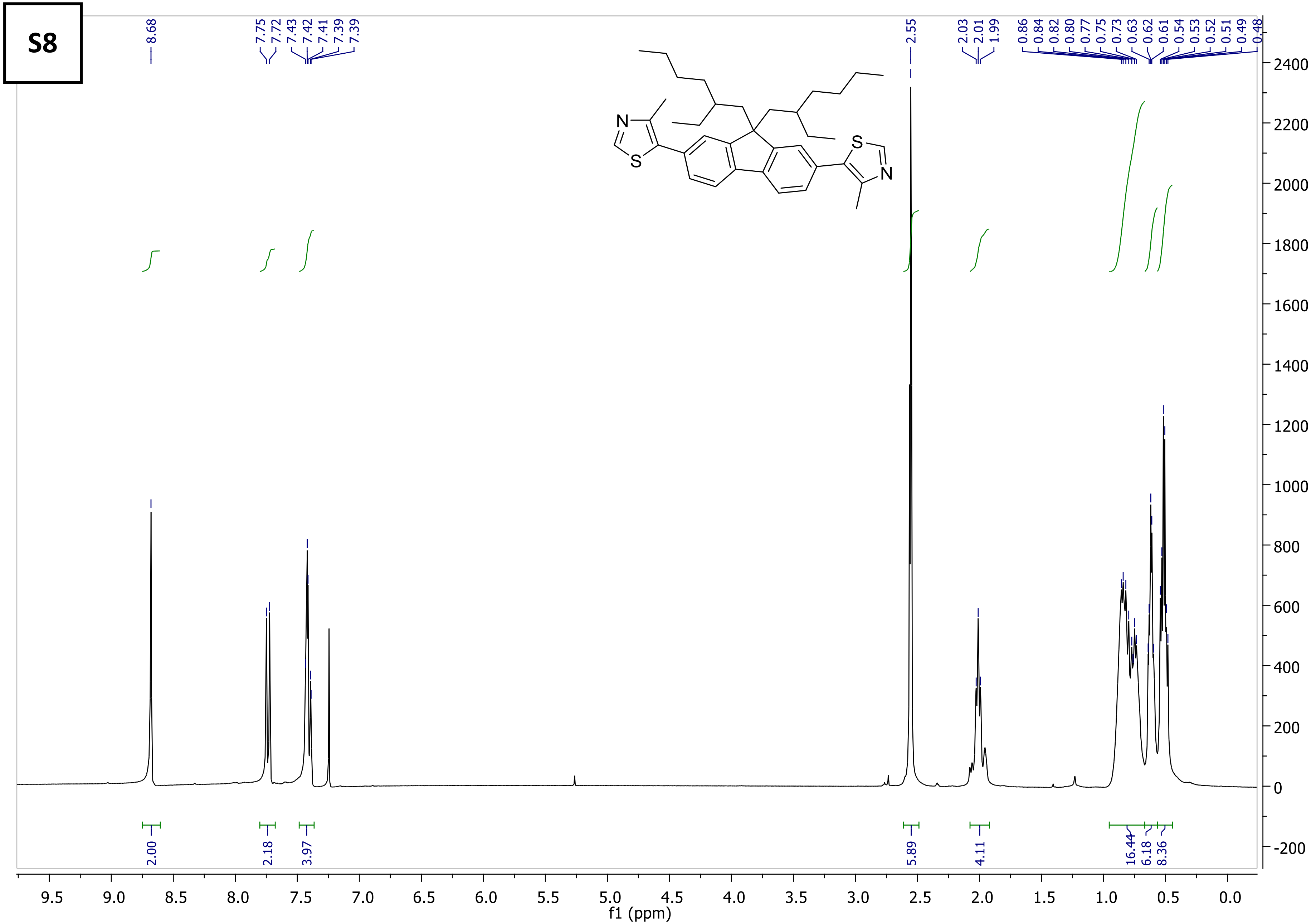
8.68
7.75
7.72
7.43
7.42
7.41
7.39
7.39
2.55
2.03
2.01
1.99
0.86
0.84
0.82
0.80
0.77
0.75
0.73
0.63
0.62
0.61
0.54
0.53
0.52
0.51
0.49
0.48
2.00
2.18
3.97
5.89
4.11
16.44
6.18
8.36
f1 (ppm)

S8

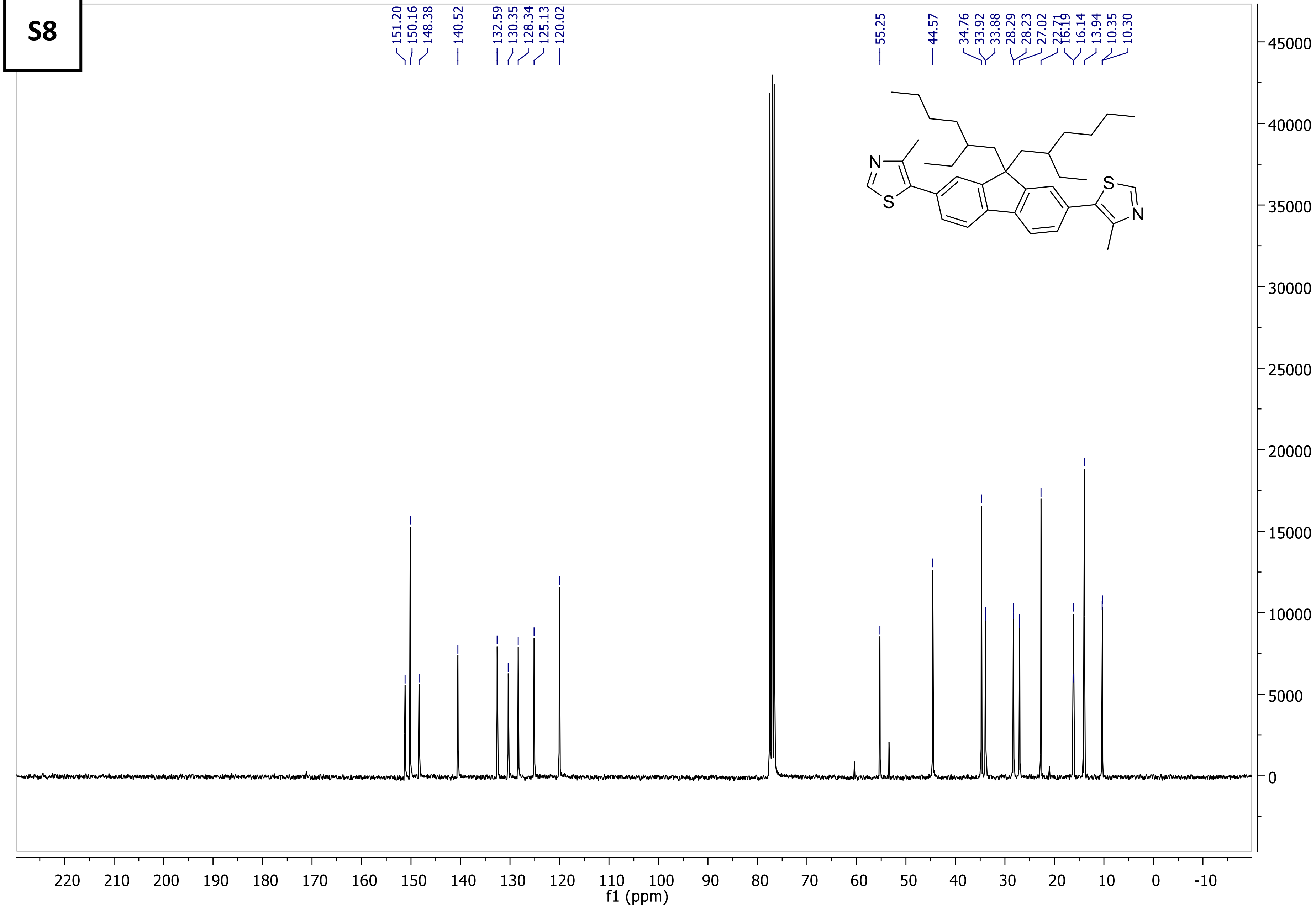
151.20
150.16
148.38
140.52
132.59
130.35
128.34
125.13
120.02
55.25
44.57
34.76
33.92
33.88
28.29
28.23
27.02
22.71
16.19
16.14
13.94
10.35
10.30
N
S
S
N
45000
40000
35000
30000
25000
20000
15000
10000
5000
0
220 210 200 190 180 170 160 150 140 130 120 110 100 90 80 70 60 50 40 30 20 10 0 -10
f1 (ppm)

S9

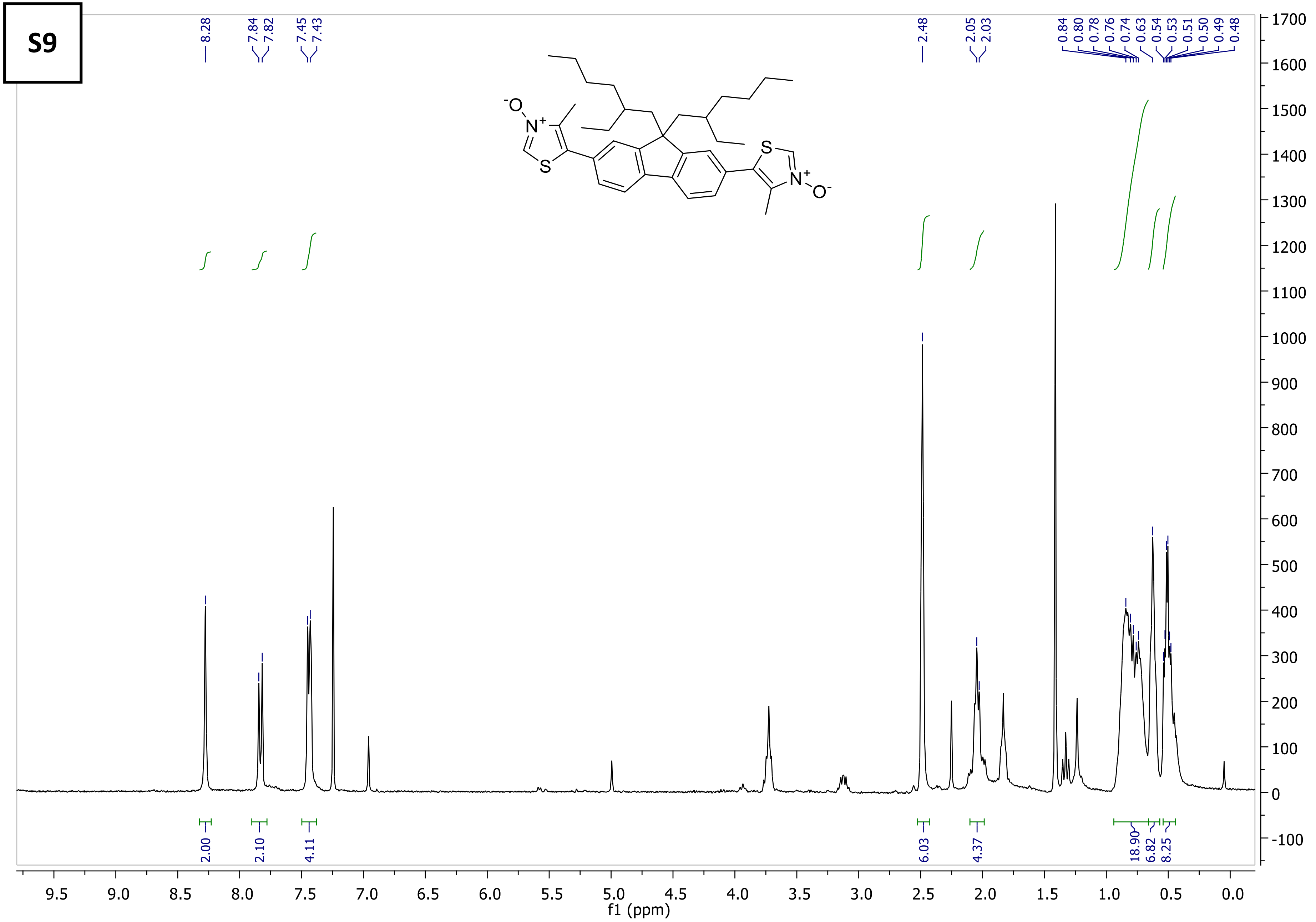
8.28
7.84
7.82
7.45
7.43
2.48
2.05
2.03
0.84
0.80
0.78
0.76
0.74
0.63
0.54
0.53
0.51
0.50
0.49
0.48
2.00
2.10
4.11
6.03
4.37
18.90
6.82
8.25
f1 (ppm)

S9

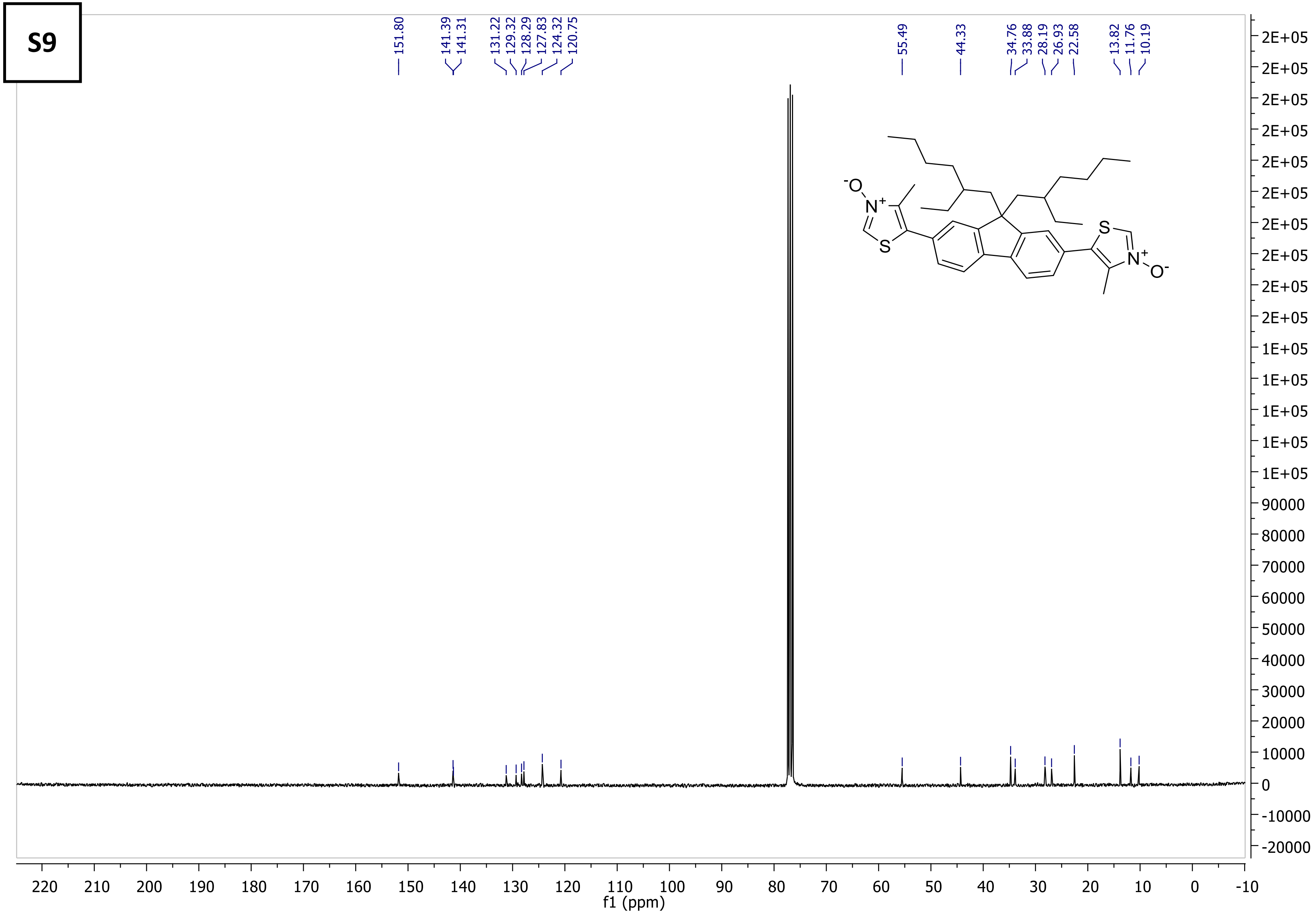
151.80
141.39
141.31
131.22
129.32
128.29
127.83
124.32
120.75
55.49
44.33
34.76
33.88
28.19
26.93
22.58
13.82
11.76
10.19
f1 (ppm)

S10

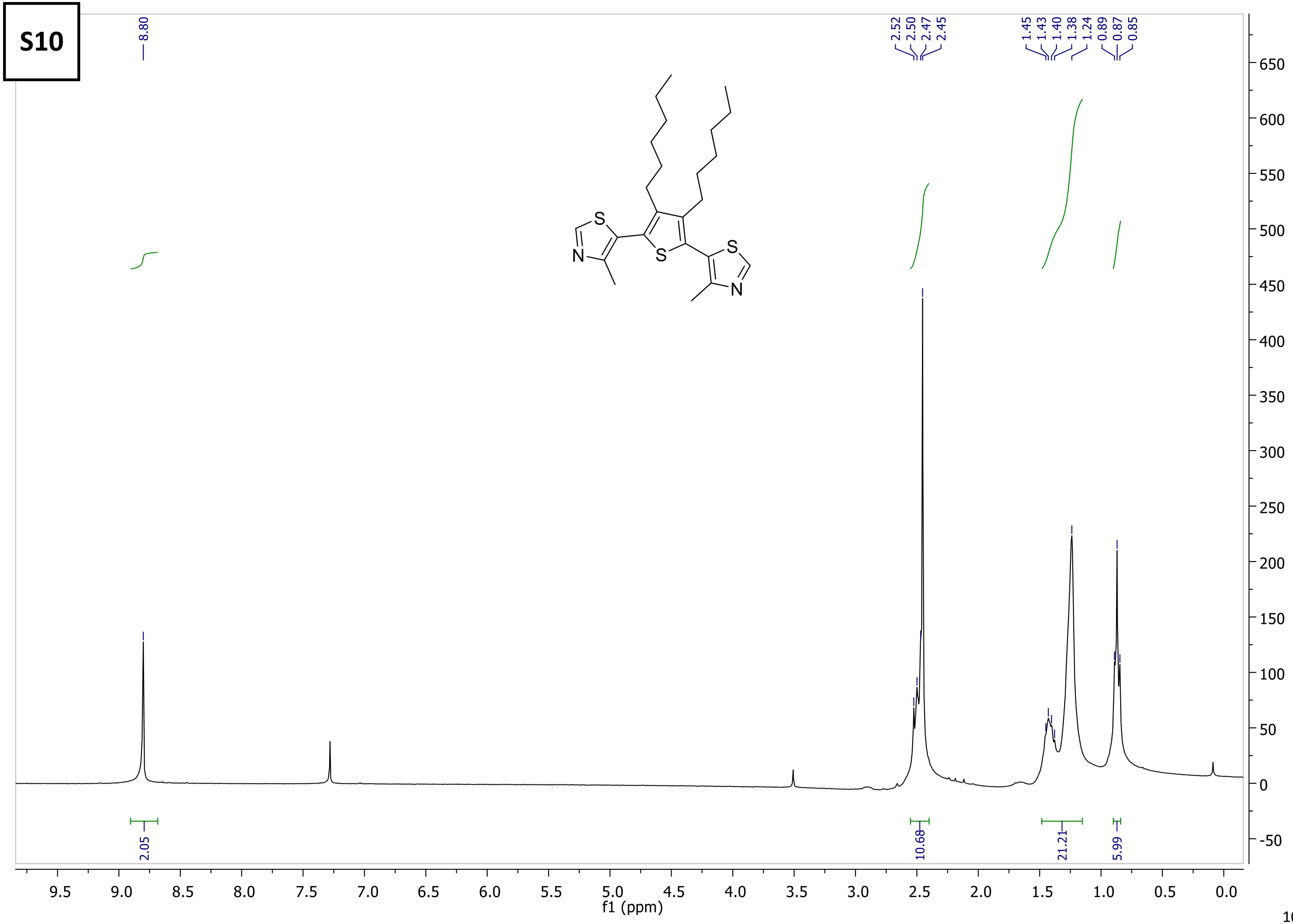

8.80
2.52
2.50
2.47
2.45
1.45
1.43
1.40
1.38
1.24
0.89
0.87
0.85
S
N
S
S
N
2.05
10.68
21.21
5.99
f1 (ppm)

S10

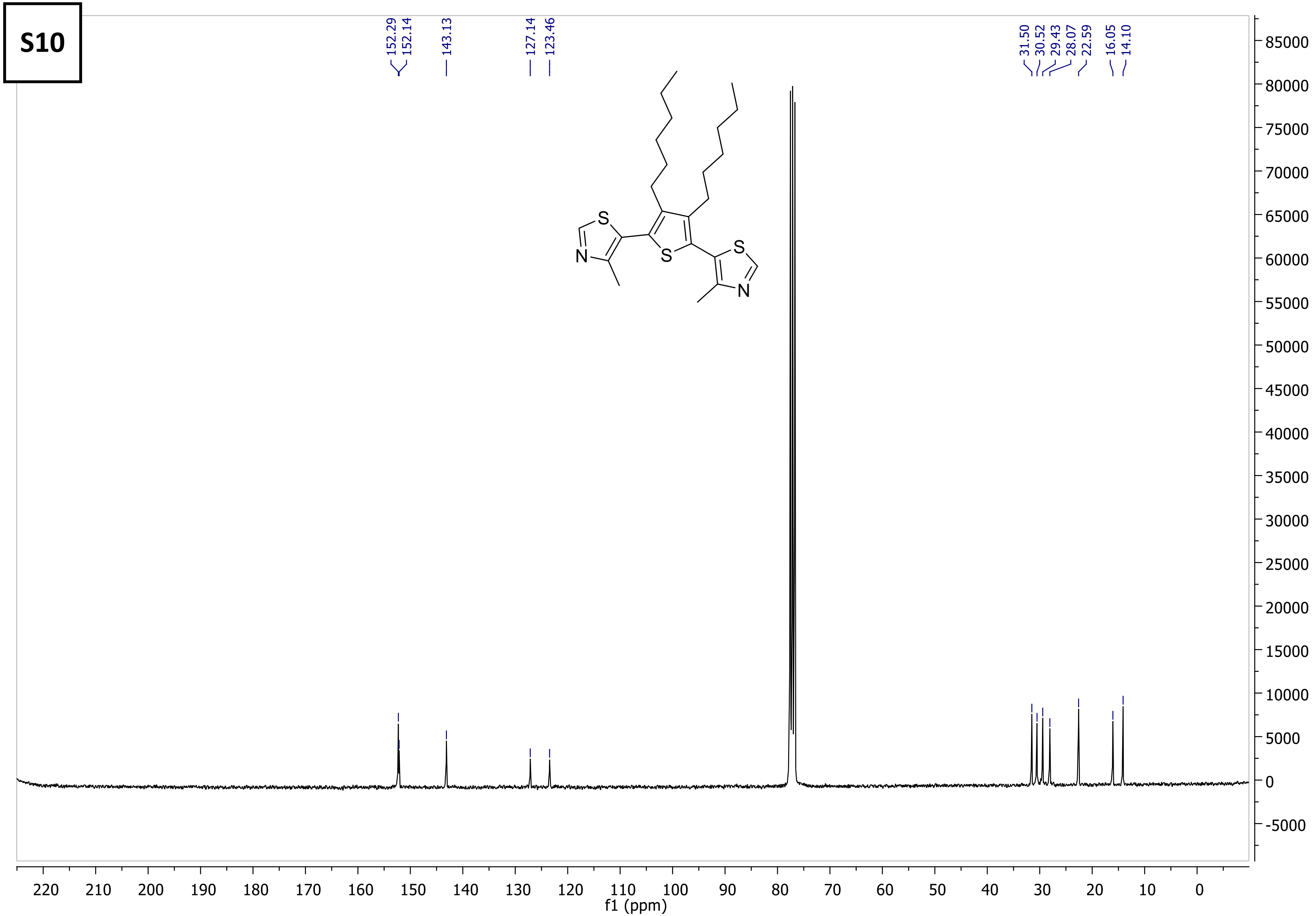
152.29
152.14
143.13
127.14
123.46
31.50
30.52
29.43
28.07
22.59
16.05
14.10
S
N
S
S
N
f1 (ppm)

S11

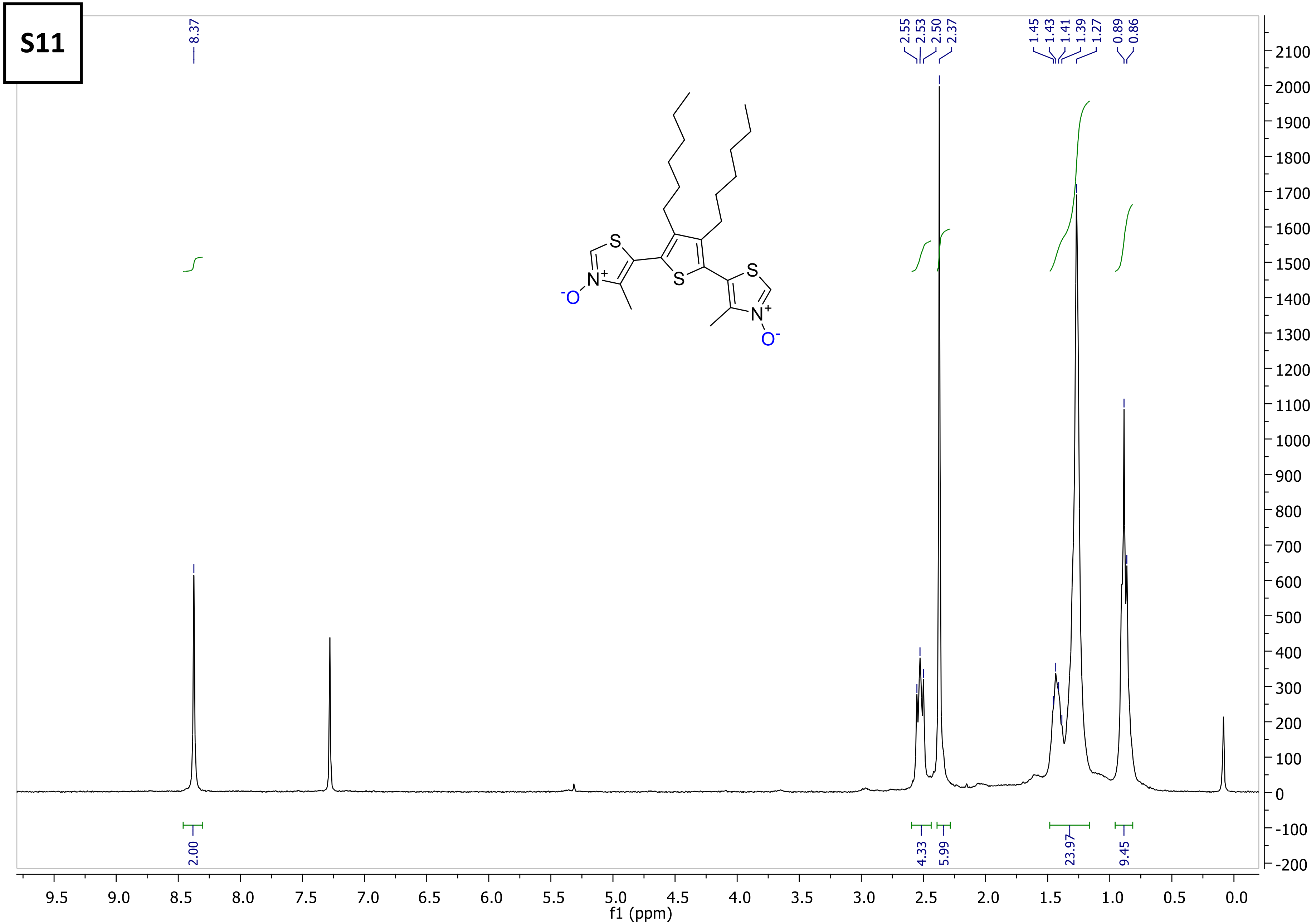
8.37
2.55
2.53
2.50
2.37
1.45
1.43
1.41
1.39
1.27
0.89
0.86
2.00
4.33
5.99
23.97
9.45
f1 (ppm)

S11

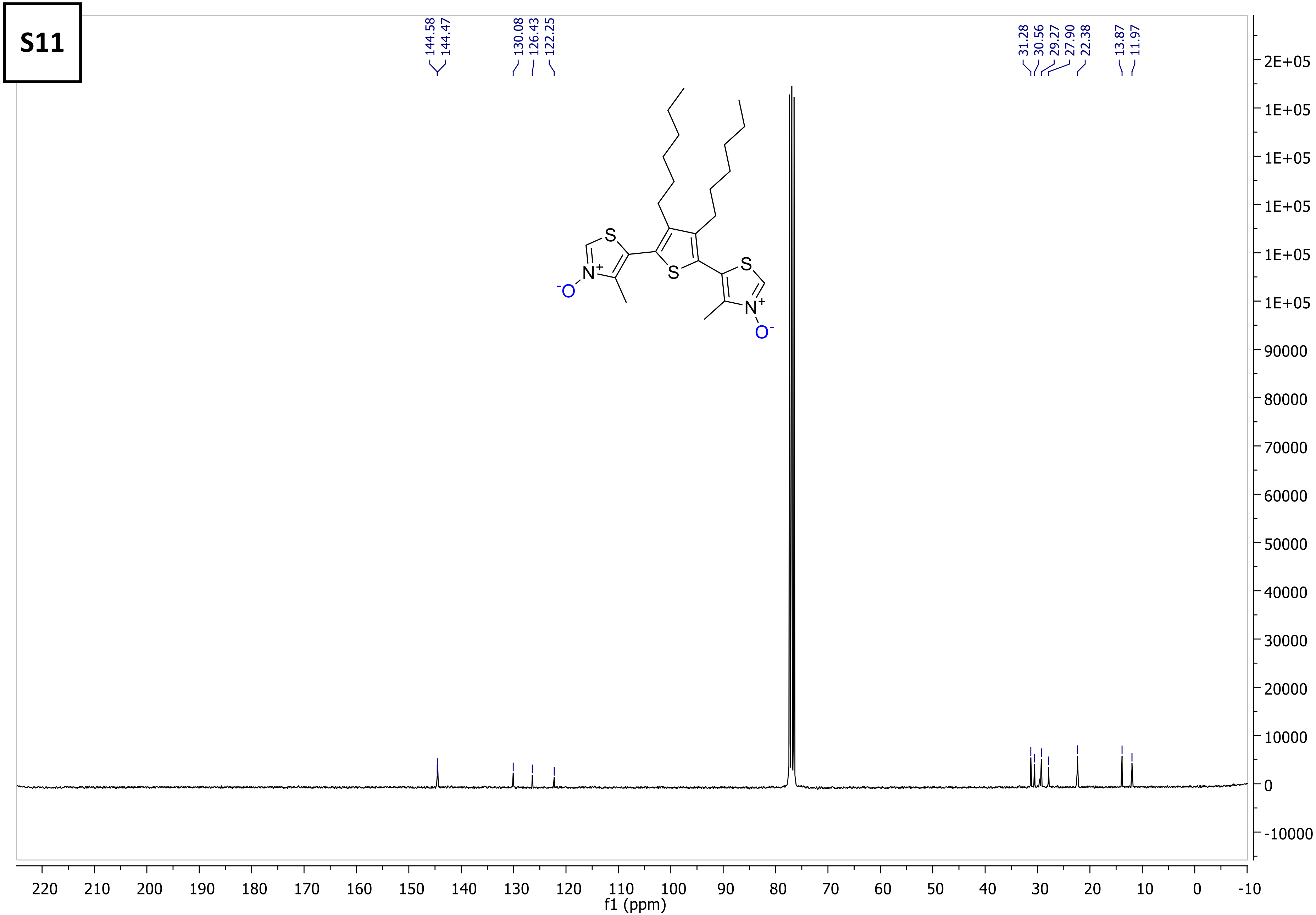

144.58
144.47
130.08
126.43
122.25
31.28
30.56
29.27
27.90
22.38
13.87
11.97
S
N+
-O
S
S
N+
O-
2E+05
1E+05
1E+05
1E+05
1E+05
1E+05
90000
80000
70000
60000
50000
40000
30000
20000
10000
0
-10000
220 210 200 190 180 170 160 150 140 130 120 110 100 90 80 70 60 50 40 30 20 10 0 -10
f1 (ppm)
